# Bis‐Squaramide‐Based [2]Rotaxane Hosts for Anion Recognition

**DOI:** 10.1002/chem.202402731

**Published:** 2024-10-29

**Authors:** Arya Arun, Andrew Docker, Paul D. Beer

**Affiliations:** ^1^ Chemistry Research Laboratory, Department of Chemistry University of Oxford Mansfield Road Oxford OX1 3TA UK

**Keywords:** Bis-squaramide, Rotaxane, Host-guest recognition, Active metal template, Anti-Hofmeister

## Abstract

The first examples of bis‐squaramide axle containing [2]rotaxanes linked *via* rigid aryl and flexible alkyl spacers synthesised using copper(I) catalysed active metal template methodology are reported. The halide and oxoanion binding properties of the [2]rotaxanes in aqueous‐organic solvent media are examined through extensive ^1^H‐NMR titration experiments to investigate the impact of integrating multiple squaramide motifs on the anion binding capabilities of the interlocked receptors. These studies reveal that the bis‐squaramide rotaxane host systems exhibit enhanced halide anion binding capabilities relative to a mono‐squaramide axle functionalised rotaxane, demonstrating a rare anti‐Hofmeister bias halide anion selectivity trend in aqueous‐organic mixtures and highlighting the efficacy of the potent solvent shielded hydrophobic interlocked binding pocket created upon mechanical bond formation. Notably, employing a rigid aryl linker between the two squaramide motifs in the axle component enables the rotaxane host to exhibit strong and selective binding of tetrahedral oxoanions. Conversely, a flexible alkyl spacer facilitates trigonal oxoanion selective recognition by the bis‐squaramide [2]rotaxane.

## Graphical abstract

Incorporating two squaramide motifs into the axle component of [2]rotaxanes dramatically enhances their anion binding affinities and selectivity properties compared to a mono‐squaramide axle containing [2]rotaxane, revealing a rare anti‐Hofmeister halide anion binding trend in aqueous‐organic solvent media. Varying the rigidity of the linker connecting the axle squaramides results in the rotaxanes displaying strong and selective binding of tetrahedral and trigonal oxoanions.

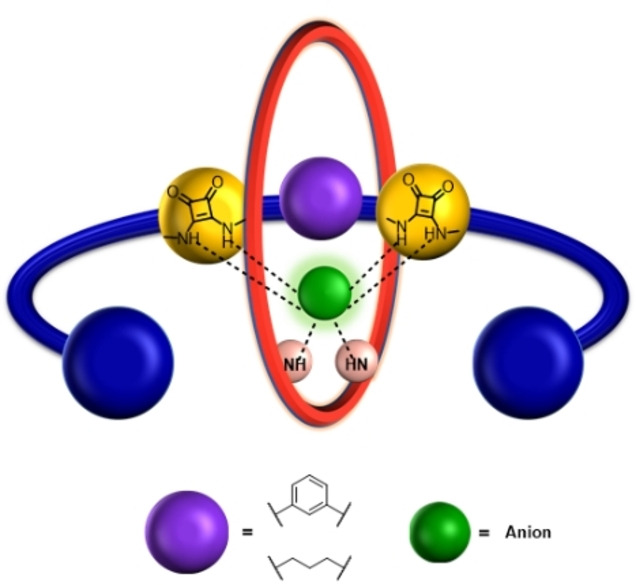

## Introduction

Anions are ubiquitous in nature and are involved across a myriad of chemical and biological processes,[^1^, ^2^, ^3^] providing strong impetus for the design of molecular host systems capable of strong and selective recognition. However, anions possess intrinsic properties such as pH‐dependence and high free energies of solvation which make their recognition a formidable task,[^4^, ^5^, ^6^, ^7^, ^8^] especially in aqueous containing media.[^9^, ^10^, ^11^] Whilst the introduction of multiple positive charges has been commonly utilised as a potential strategy to overcome the effects of anion solvation in competitive protic aqueous solvent media, this is often to the detriment of selectivity.^[12]^ Efforts directed towards the construction of host architectures capable of achieving anion recognition in aqueous media have identified mechanically interlocked molecules (MIMs) such as rotaxanes and catenanes as potent host systems.[^13^, ^14^, ^15^, ^16^, ^17^] The unique topological three dimensional encapsulating binding cavities afforded by the mechanical bond resemble biotic host systems which achieve strong and selective recognition of anionic species through the concerted action of multiple hydrogen bonding (HB) donor motifs.[^18^, ^19^, ^20^, ^21^, ^22^, ^23^, ^24^]

**Figure 1:**
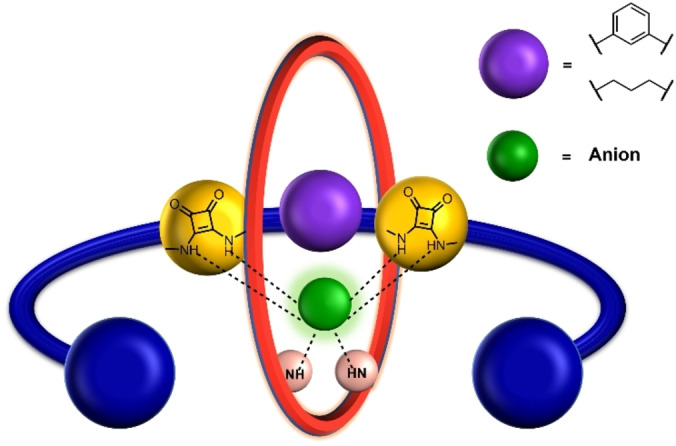
Cartoon representation of the target bis‐squaramide‐axle based [2]rotaxane host systems.

Taking inspiration from this approach we sought to integrate the prodigious anion recognition capabilities of squaramide HB donor units into interlocked [2]rotaxane host structures for the purposes of anion recognition in aqueous containing media. While there are numerous examples of squaramide‐based acyclic and macrocyclic host systems,[^25^, ^26^, ^27^, ^28^, ^29^, ^30^, ^31^, ^32^, ^33^] MIMs containing a squaramide motif are rare.[^34^, ^35^, ^36^] Herein, we report a series of unprecedented bis‐squaramide functionalised axle component containing [2]rotaxanes, wherein the two squaramide units are linked *via* an aryl or aliphatic spacer (Figure 1). Extensive ^1^H‐NMR binding investigations reveal notable enhanced anion binding affinities relative to a mono‐squaramide axle functionalised [2]rotaxane analogue, with the bis‐squaramide‐based [2]rotaxanes exhibiting rare anti‐Hofmeister bias halide anion selectivity trends in aqueous‐organic mixtures. Furthermore, the rigidity of the alkyl/aryl linker between the squaramide units markedly influences binding strength and importantly oxoanion selectivity.

## Results and Discussion

### Synthesis and Characterisation

The synthesis of the target [2]rotaxanes began with the preparation of the bis‐azide functionalised bis‐squaramide axle precursors, **4** and **5**, via modified literature procedures.[^37^, ^38^]

**Scheme 1:**
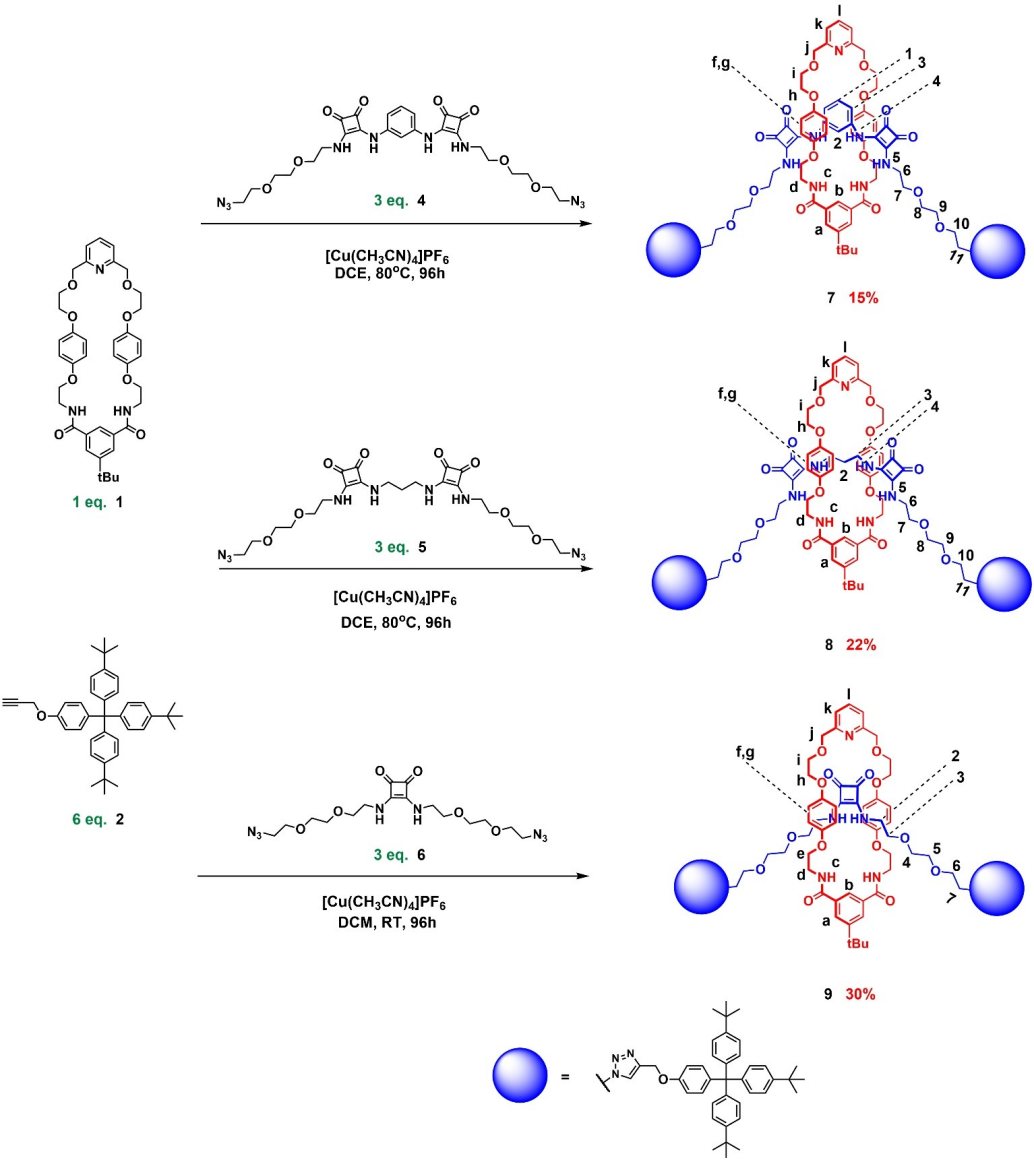
Synthesis of bis‐squaramide [2]rotaxanes **7**, **8** and mono‐squaramide [2]rotaxane **9** using active metal template methodology.

Thereafter, active metal template methodology^[39]^ was employed to synthesise the bis‐squaramide‐based [2]rotaxanes. In a typical reaction, an equimolar mixture of macrocycle **1** ^[40]^ and Cu(CH_3_CN)_4_PF_6_ in dichloroethane was combined with six and three equivalents of stopper alkyne **2** ^[41]^ and bis‐azide precursor (**4** or **5**) respectively (Scheme 1). The reaction mixture was left to stir at 80 °C for three days following which the crude mixture was subjected to an aqueous workup with EDTA/NH_4_OH. Purification by preparative TLC afforded the target [2]rotaxanes **7** and **8** in 15 % and 22 % yields respectively. Both novel [2]rotaxanes were characterised *via* ^1^H‐NMR, ^13^C‐NMR, ^1^H‐^1^H ROESY NMR and ESI‐HRMS.

**Figure 2:**
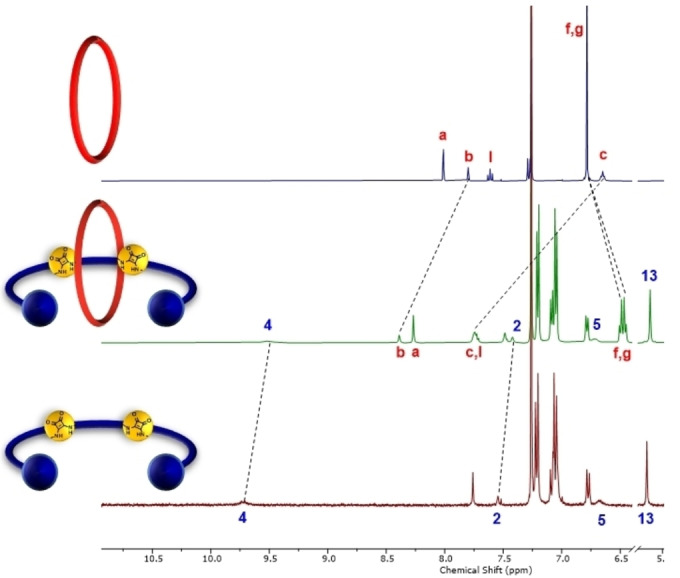
Truncated stacked ^1^H‐NMR spectra of i) macrocycle **1** ii) [2]rotaxane **7** and iii) axle component **7a** (CDCl_3_, 500 MHz, 298 K).

Evidence of [2]rotaxane formation was obtained by comparison of the ^1^H‐NMR spectra of the interlocked product, representative example [2]rotaxane **7**, and separate macrocycle and axle components in CDCl_3_ (Figure 2). Noteworthy upfield shifts and splitting of the rotaxane's hydroquinone protons H_f,g_ as a result of donor‐acceptor interactions between the axle electron deficient squaramide and electron‐rich macrocycle hydroquinone components were indicative of successful mechanical bond formation. Furthermore, the downfield shifts of the macrocycle protons H_b_ and H_c_ and upfield shifts of the axle protons H_2_ and H_4_ were attributed to shielding effects arising from the macrocycle residing in the vicinity of the two squaramide motifs, a co‐conformation presumably stabilised by extensive intercomponent HB interactions. Similar diagnostic ^1^H‐NMR shifts were observed in the case of [2]rotaxane **8** as well. Additionally, ESI‐MS analysis of the sample mixtures revealed *m/z* peaks at 2382 and 2348 Da, corresponding to the target [2]rotaxanes.

**Figure 3:**
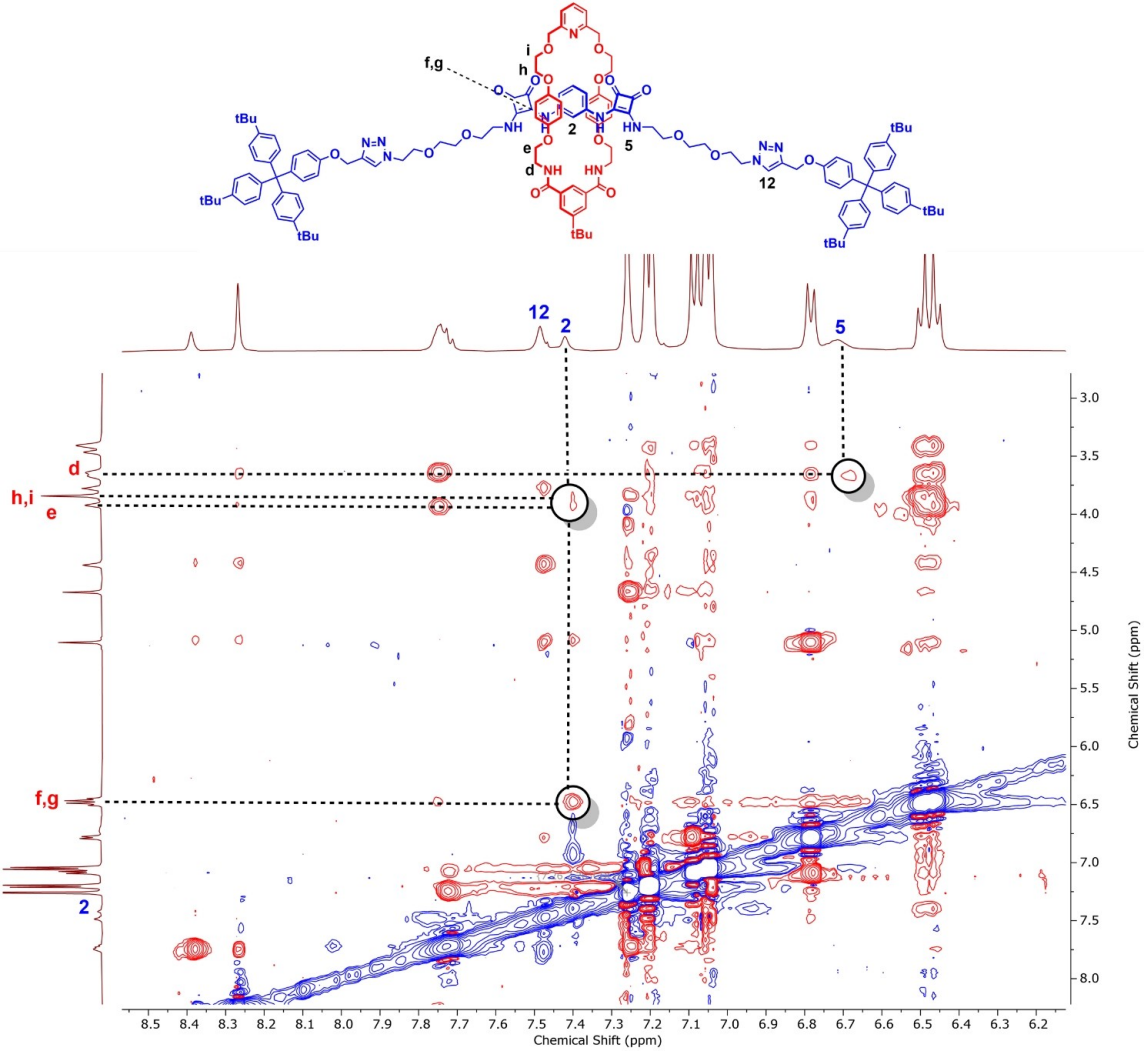
Truncated ^1^H‐^1^H ROESY NMR spectrum of [2]rotaxane **7** in CDCl_3_

A ^1^H‐^1^H ROESY NMR study revealed further evidence for mechanical bond formation. In the case of both [2]rotaxanes **7** and **8**, through‐space interactions were evinced between the axle squaramide protons and the protons of the macrocycle, while none were observed between the axle triazole and the macrocycle protons, consistent with the macrocycle preferentially residing at the respective bis‐squaramide station of the axle (Figure 3 and S18).

Having characterised the target bis‐squaramide containing [2]rotaxanes, the synthesis of previously reported mono‐squaramide [2]rotaxane **9** ^[34]^ was undertaken for the purposes of anion binding comparison with this bis‐squaramide rotaxane systems. Formerly prepared by employing a CuAAC stoppering MIM procedure of a NaBAr^F^_4_ templated [2]pseudorotaxane assembly between a bis‐azide functionalised polyether squaramide axle precursor and macrocycle, [2]rotaxane **9** was synthesised again in an improved yield of 30 % using AMT methodology (Scheme 1).

### Anion Binding Studies

The anion recognition properties of bis‐squaramide axle containing rotaxanes **7** and **8**, together with mono‐squaramide axle rotaxane **9** for comparison, were investigated *via* ^1^H‐NMR titration studies in aqueous‐acetone solvent mixtures. A variety of anions were selected including halides and a range of oxoanions to investigate the role of anion geometry on binding affinity and selectivity. In a typical experiment, aliquots of TBA salts were sequentially added to a 1 mM solution of each rotaxane in 5 : 95 D_2_O/d_6_‐acetone solvent media.

**Figure 4:**
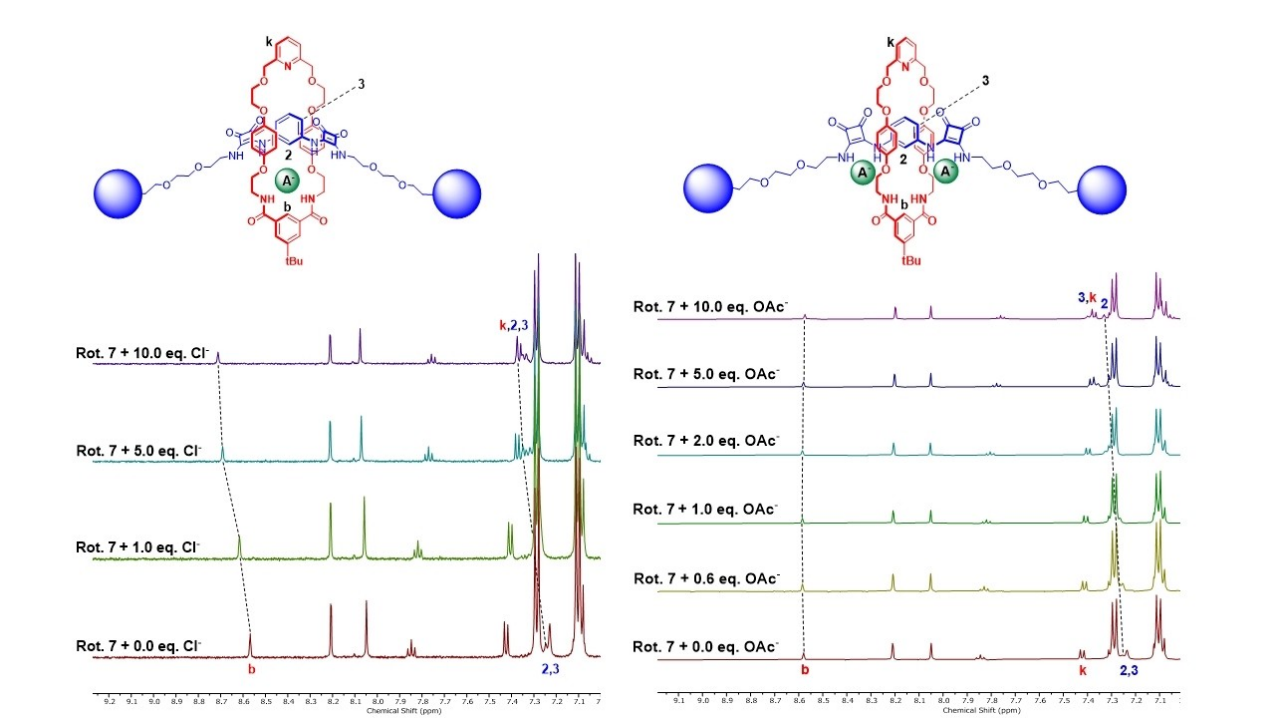
^1^H‐NMR spectra of [2]rotaxane **7** upon addition of 1, 5 and 10 equivalents of a) TBACl and b) TBAOAc (500 MHz, 298 K, 5 : 95‐D_2_O‐d_6_‐acetone). The amide resonances of the macrocycle and axle components were not observed in this protic solvent medium due to hydrogen‐deuterium exchange.

In the case of the aryl‐linked [2]rotaxane **7**, the addition of the majority of anions led to downfield shifts in the proton signals corresponding to the chemical environments adjacent to the NH HB donors, namely macrocycle proton H_b_ and axle spacer proton H_2_ indicative of binding occurring within the interlocked cavity of the [2]rotaxane (Figure 4a). Intriguingly, upon addition of oxoanions, OAc^−^, HSO_4_^−^ and H_2_PO_4_^−^ the proton signals corresponding to environments proximal to the anion binding site initially moved downfield until 0.8–1 equivalents before shifting upfield upon subsequent anion addition of up to 10 equivalents (Figure 4b) alluding to the existence of complex binding equilibria in the system.

Alternatively, in the case of alkyl‐linked bis‐squaramide [2]rotaxane **8** and mono‐squaramide [2]rotaxane **9**, upon increasing anion concentration downfield shifts in the internal benzene proton of the macrocycle H_b_ and the axle spacer proton H_2_ were observed for all anions. Additionally, notable perturbations in the proton resonances of the axle triazole motifs and neighbouring methylene protons were also seen for rotaxane **8**, suggesting their proximity to the binding cavity and potential assistance in anion recognition.

**Figure 5:**
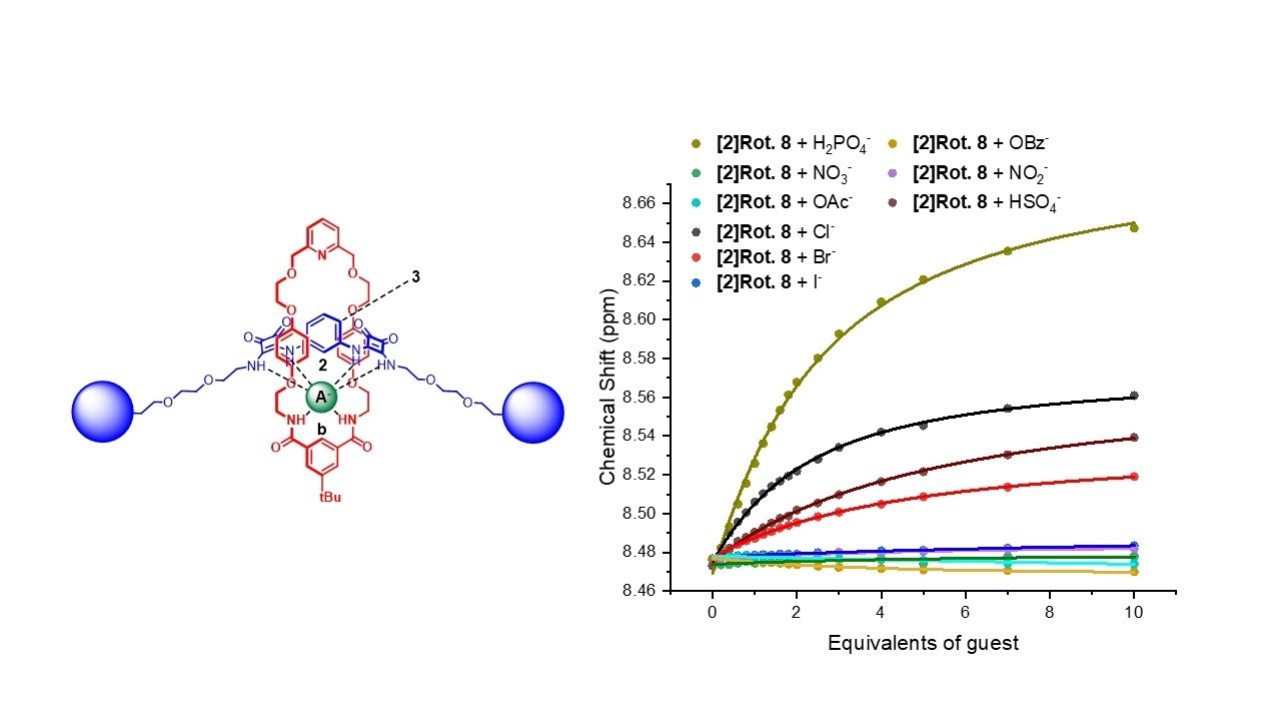
Binding isotherms of [2]rotaxane **8**, showing changes in chemical shift of internal benzene proton b upon increasing equivalents of various anions

Bindfit^[42]^ analysis of the rotaxane isotherm titration data (Figure 5, S51, S52 and S53) determined association constant values shown in Table 1, using a 1 : 1 stoichiometric host‐guest binding model for all anions, with the exception of a 1 : 2 stoichiometric host‐guest binding model for [2]rotaxane **7** and oxoanions, OAc^−^, HSO_4_^−^ and H_2_PO_4_^−^. This difference in the stoichiometric OAc^−^, HSO_4_^−^ and H_2_PO_4_ oxoanion binding modes by the bis‐squaramide rotaxanes may be attributed to the relatively more rigid aryl spacer motif between the squaramide motifs in [2]rotaxane **7** compared to the flexible propyl alkyl linking group of [2]rotaxane **8**.

**Table 1:** Anion association constants *K_a_* (M^−1^) for [2]rotaxane **7**,**8** and **9** [1.0 mM] in 95 : 5‐d_6_‐acetone‐D_2_O at 298 K.^[a]^

Anion	[2]Rot. **7**			[2]Rot. **8**		[2]Rot. **9**	
	Binding mode	*K_1_* (M^−1^)	*K_2_* (M^−1^)	Binding mode	*K_1_* (M^−1^)	Binding mode	*K_1_* (M^−1^)
Cl^−^	1 : 1	574	–	1 : 1	621	1 : 1	90
Br^−^	1 : 1	225	–	1 : 1	226	1 : 1	34
I^−^	1 : 1	35	–	1 : 1	58	1 : 1	–^[b]^
NO_3_^−^	1 : 1	42	–	1 : 1	130	1 : 1	–^[b]^
NO_2_^−^	1 : 1	124	–	1 : 1	160	1 : 1	–^[b]^
OBz^−^	1 : 1	701	–	1 : 1	916	1 : 1	46
OAc^−^	1 : 2	3279 (10)	232	1 : 1	668	1 : 1	317
HSO_4_^−^	1 : 2	5533 (23)	1383	1 : 1	224	1 : 1	223
H_2_PO_4_^−^	1 : 2	3627	906	1 : 1	451	1 : 1	38

[a] *K_a_* values calculated using Bindfit^[42]^ software using a 1 : 1 and 1 : 2 host‐guest binding models by monitoring internal benzene proton H_b_. All anions added as their TBA salts. Error percentages less than 10 % unless specified. [b] No binding.

Analysis of the binding data in Table 1 reveals a considerable amplification in anion binding affinities across the board for the bis‐squaramide hosts relative to the mono‐squaramide [2]rotaxane **9**, highlighting the advantages of incorporating potent multi‐squaramide HB donor binding sites into MIM host design. Amongst the halides, both bis‐squaramide rotaxane receptors exhibit selectivity for chloride, with a 6–7‐fold enhancement observed in the case of chloride and bromide association constant values relative to the mono‐squaramide axle containing rotaxane **9**. Furthermore, the halide binding data is also indicative of a notable anti‐Hofmeister trend (Cl^−^>Br^−^>I^−^) in the aqueous containing organic solvent mixture, attributable to the effective hydrophobic interlocked host cavity created upon mechanical bond formation.

In the case of the oxoanions, receptor **7** exhibits much stronger binding of tetrahedral shaped anions namely HSO_4_^−^ and H_2_PO_4_^−^, displaying remarkable 25‐fold and 95‐fold enhancements in their *K_1_* binding constant magnitudes in comparison to rotaxane **9**. An oxoanion selectivity trend of HSO_4_^−^>H_2_PO_4_^−^>OAc^−^>OBz^−^>NO_3_^−^>NO_2_^−^ is observed, indicative of augmented MIM host‐guest complementarity for tetrahedral anion guest species. By contrast rotaxane **8** exhibits a modest preference for the trigonally shaped basic OBz^−^/OAc^−^ oxoanions. Notably, NO_3_^−^ and NO_2_^−^ are relatively weakly bound by both bis‐squaramide rotaxanes.

## Conclusions

In an effort to enhance anion recognition capability, two novel bis‐squaramide functionalised axle containing [2]rotaxanes were designed and constructed using active metal template mechanical bond synthetic methodology. Extensive ^1^H‐NMR anion titration studies in aqueous‐organic mixtures demonstrated the introduction of a second squaramide HB donor motif into the respective rotaxane's axle component resulted in significant amplification in halide and oxoanion anion binding affinity as compared to a mono‐squaramide axle functionalised [2]rotaxane host analogue. In the case of halides, the bis‐squaramide rotaxane host systems displayed an anti‐Hofmeister bias halide anion selectivity for chloride, a rare phenomenon brought into effect by a combination of the MIM hydrophobic cavity and strong HB donor binding capabilities of the bis‐squaramide axle components. The contrasting rigidity of the alkyl/aryl linker between the two squaramide motifs of the axle component of the respective [2]rotaxane, notably influences oxoanion binding strength and selectivity. The aryl spacer functionalised bis‐squaramide rotaxane (**7**) selectively bound tetrahedral anions such as H_2_PO_4_^−^ and HSO_4_^−^, while the incorporation of flexible propyl alkyl linker in bis‐squaramide rotaxane (**8**) resulted in a preference for trigonally shaped basic oxoanions OBz^−^ and OAc^−^. These observations serve to highlight the advantages of integrating multiple potent HB donor squaramide motifs into future MIM host structural design to achieve enhanced anion binding strength and influence selectivity.

## Conflict of Interests

The authors declare no conflict of interest.

1

## Supporting information

As a service to our authors and readers, this journal provides supporting information supplied by the authors. Such materials are peer reviewed and may be re‐organized for online delivery, but are not copy‐edited or typeset. Technical support issues arising from supporting information (other than missing files) should be addressed to the authors.

Supporting Information

## Data Availability

The data that support the findings of this study are available in the supplementary material of this article.

## References

[1] P. A. Gale , Chem. Commun. 2011, 47, 82–86. 10.1039/c0cc00656d 20520917

[2] N. Busschaert , C. Caltagirone , W. Van Rossom , P. A. Gale , Chem. Rev. 2015, 115, 8038–8155. 25996028 10.1021/acs.chemrev.5b00099

[3] M. Teresa Albelda , J. C. Frías , E. García-España , H.-J. Schneider , Chem. Soc. Rev. 2012, 41, 3859–3877. 22441360 10.1039/c2cs35008d

[4] R. Shannon , Acta. Crystallogr. 1976, 32, 751–767.

[5] K. H. Stern , E. S. Amis , Chem. Rev. 1959, 59, 1–64.

[6] J. W. Steed, J. L. Atwood, *Supramolecular Chemistry*, John Wiley & Sons **2022**.

[7] A. E. Hargrove , S. Nieto , T. Zhang , J. L. Sessler , E. V. Anslyn , Chem. Rev. 2011, 111, 6603–6782. 21910402 10.1021/cr100242s PMC3212652

[8] P. D. Beer , P. A. Gale , Angew. Chem. Int. Ed. 2001, 40, 486–516. 11180358

[9] M. J. Langton , C. J. Serpell , P. D. Beer , Angew. Chem. Int. Ed. 2016, 55, 1974–1987. 10.1002/anie.201506589 PMC4755225 26612067

[10] M. G. Cacace , E. M. Landau , J. J. Ramsden , Q. Rev. Biophys. 1997, 30, 241–277. 9394422 10.1017/s0033583597003363

[11] S. Kubik , Chem. Soc. Rev. 2010, 39, 3648–3663. 20617241 10.1039/b926166b

[12] H.-J. Schneider , A. K. Yatsimirsky , Chem. Soc. Rev. 2008, 37, 263–277. 18197343 10.1039/b612543n

[13] M. J. Langton , S. W. Robinson , I. Marques , V. Félix , P. D. Beer , Nat. Chem. 2014, 6, 1039–1043. 25411880 10.1038/nchem.2111

[14] A. Docker , Y. C. Tse , H. M. Tay , A. J. Taylor , Z. Zhang , P. D. Beer , Angew. Chem. Int. Ed. 2022, 61, e202214523. 10.1002/anie.202214523 PMC10100147 36264711

[15] L. M. Hancock , E. Marchi , P. Ceroni , P. D. Beer , Chem. Eur. J. 2012, 18, 11277–11283. 22847976 10.1002/chem.201201422

[16] R. C. Knighton , S. Dapin , P. D. Beer , Chem. Eur. J. 2020, 26, 5288–5296. 32130744 10.1002/chem.202000661 PMC7216984

[17] M. J. Langton , O. A. Blackburn , T. Lang , S. Faulkner , P. D. Beer , Angew. Chem. Int. Ed. 2014, 53, 11463–11466. 10.1002/anie.201405131 PMC4497609 24989322

[18] H. Luecke , F. A. Quiocho , Nat. 1990, 347, 402–406. 10.1038/347402a0 2215649

[19] J. W. Pflugrath , F. A. Quiocho , Nat. 1985, 314, 257–260. 10.1038/314257a0 3885043

[20] A. Borissov , I. Marques , J. Y. C. Lim , V. Félix , M. D. Smith , P. D. Beer , J. Am. Chem. Soc. 2019, 141, 4119–4129. 30730716 10.1021/jacs.9b00148

[21] T. Bunchuay , A. Docker , A. J. Martinez-Martinez , P. D. Beer , Angew. Chem. 2019, 131, 13961–13965. 10.1002/anie.201907625 31291498

[22] R. Pereira Orenha , S. S. Pereira Furtado , A. Muñoz-Castro , M. Jeomar Piotrowski , G. Finoto Caramori , R. L. Tame Parreira , Chem. Eur. J. 2023, 29, e202203905. 36847391 10.1002/chem.202203905

[23] J. P. Byrne , S. Blasco , A. B. Aletti , G. Hessman , T. Gunnlaugsson , Angew. Chem. Int. Ed. 2016, 55, 8938–8943. 10.1002/anie.201603213 27295556

[24] R. J. Goodwin , A. Docker , H. I. MacDermott-Opeskin , H. M. Aitken , M. L. O'Mara , P. D. Beer , N. G. White , Chem. Eur. J. 2022, 28, e202200389. 35293643 10.1002/chem.202200389 PMC9321576

[25] V. Amendola , L. Fabbrizzi , L. Mosca , F.-P. Schmidtchen , Chem. Eur. J. 2011, 17, 5972–5981. 21472802 10.1002/chem.201003411

[26] R. B. P. Elmes , K. K. Y. Yuen , K. A. Jolliffe , Chem. Eur. J. 2014, 20, 7373–7380. 24828677 10.1002/chem.201400292

[27] C. Jin , M. Zhang , C. Deng , Y. Guan , J. Gong , D. Zhu , Y. Pan , J. Jiang , L. Wang , Tetrahedron Lett. 2013, 54, 796–801.

[28] L. Fan , T. Xu , J. Feng , Z. Ji , L. Li , X. Shi , C. Tian , Y. Qin , Electroanalysis 2020, 32, 805–811.

[29] R. P. Orenha , V. B. da Silva , G. F. Caramori , F. S. de Souza Schneider , M. J. Piotrowski , J. Contreras-Garcia , C. Cardenas , M. B. Gonçalves , F. Mendizabal , R. L. T. Parreira , New. J. Chem. 2020, 44, 17831–17839.

[30] A. A. Abogunrin , S. A. Healy , O. Fenelon , R. B. P. Elmes , Chem. 2022, 4, 1288–1299.

[31] J. D. E. Lane , K. A. Jolliffe , Org. Biomol. Chem. 2023, 21, 3226–3234. 36988418 10.1039/d3ob00069a

[32] L. K. Kumawat , A. A. Abogunrin , M. Kickham , J. Pardeshi , O. Fenelon , M. Schroeder , R. B. P. Elmes , Front. Chem. 2019, 7, 354. 31192187 10.3389/fchem.2019.00354 PMC6540876

[33] G. Picci , R. Montis , V. Lippolis , C. Caltagirone , Chem. Soc. Rev. 2024, 53, 3952–3975. 38465875 10.1039/d3cs01165h

[34] A. Arun , A. Docker , H. Min Tay , P. D. Beer , Chem. Eur. J. 2023, 29, e202301446. 37300836 10.1002/chem.202301446 PMC10946609

[35] J. Beswick , V. Blanco , G. De Bo , D. A. Leigh , U. Lewandowska , B. Lewandowski , K. Mishiro , Chem. Sci. 2015, 6, 140–143. 28553462 10.1039/c4sc03279a PMC5424444

[36] R. L. Spicer , C. C. Shearman , N. H. Evans , Chem. Eur. J. 2023, 29, e202203502. 36602422 10.1002/chem.202203502

[37] R. Ian Storer , C. Aciro , L. H. Jones , Chem. Soc. Rev. 2011, 40, 2330–2346. 21399835 10.1039/c0cs00200c

[38] A. Rostami , A. Colin , X. Y. Li , M. G. Chudzinski , A. J. Lough , M. S. Taylor , J. Org. Chem. 2010, 75, 3983–3992. 20486682 10.1021/jo100104g

[39] J. Berná , J. D. Crowley , S. M. Goldup , K. D. Hänni , A.-L. Lee , D. A. Leigh , Angew. Chem. Int. Ed. 2007, 46, 5709–5713. 10.1002/anie.200701678 17594702

[40] M. R. Sambrook , P. D. Beer , J. A. Wisner , R. L. Paul , A. R. Cowley , F. Szemes , M. G. B. Drew , J. Am. Chem. Soc. 2005, 127, 2292–2302. 15713109 10.1021/ja046278z

[41] V. Aucagne , K. D. Hänni , D. A. Leigh , P. J. Lusby , D. B. Walker , J. Am. Chem. Soc. 2006, 128, 2186–2187. 16478152 10.1021/ja056903f

[42] P. Thordarson , Chem. Soc. Rev. 2011, 40, 1305–1323. 21125111 10.1039/c0cs00062k

[43] E. N. W. Howe , M. Bhadbhade , P. Thordarson , J. Am. Chem. Soc. 2014, 136, 7505–7516. 24824365 10.1021/ja503383e

