# Supplementary material for: Bis‐Squaramide‐Based [2]Rotaxane Hosts for Anion Recognition
Source: Chemistry. 2024 Oct 29;30(69):e202402731. doi: 10.1002/chem.202402731 (PMC11632403; doi:10.1002/chem.202402731)
Supplement: Supplementary file 1 — Supporting Information [file CHEM-30-e202402731-s001.pdf]

Chemistry–A European Journal

Supporting Information

Bis-Squaramide-Based [2]Rotaxane Hosts for Anion Recognition

Arya Arun, Andrew Docker, and Paul D. Beer*

Table of Contents

Synthesis and characterisation 2
Materials and methods 2
General procedure 1: Synthesis of bis-squaramide based azide axle precursors 4 and 5 2
General procedure 2: Synthesis of mono-squaramide based azide axle precursor 6 8
General procedure 3: Synthesis of [2]rotaxanes using active metal template methodology 10
^{1}H-NMR binding studies 26
General procedure 26
^{1}H-NMR anion binding titration studies with [2]rotaxane 7 26
^{1}H-NMR anion binding titration studies with [2]rotaxane 8 31
^{1}H-NMR anion binding titration studies with [2]rotaxane 9 36
Anion binding isotherms 39
Determination of binding models in anion titrations 41
References 45

Synthesis and characterisation

Materials and methods

All solvents and reagents were purchased from commercial suppliers and used as received unless otherwise stated. Dry solvents were obtained by purging with nitrogen and then passing through an MBraun MPSP-800 column. H_2O was de-ionized and micro filtered using a Milli-Q ® Millipore machine. Column chromatography was carried out on Merck® silica gel 60 under a positive pressure of nitrogen. Routine NMR spectra were recorded on either a Bruker AVIII 400 or a Bruker AVIII 500 spectrometer with ^{1}H-NMR titrations recorded on a Bruker AVIII 500 spectrometer. TBA salts were stored in a vacuum desiccator containing phosphorus pentoxide prior to use. Where mixtures of solvents were used, ratios are reported by volume. Chemical shifts are quoted in parts per million relative to the residual solvent peak. Mass spectra were recorded on a Bruker µTOF spectrometer. Triethylamine was distilled from and stored over potassium hydroxide. Macrocycle (**1**),[40] stopper alkyne (**2**)[41], tri(ethyleneglycol)-based azide amine (**3i**),[34] were prepared according to previous literature reports.

General procedure 1: Synthesis of bis-squaramide based azide axle precursors 4 and 5

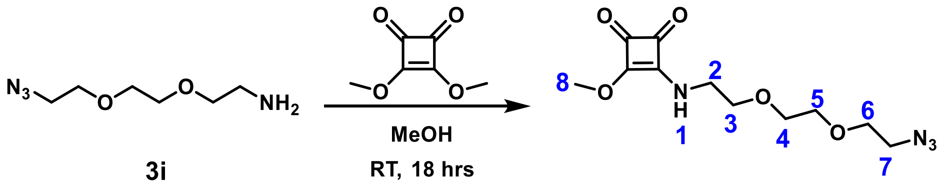

Squaramide tri(ethylene glycol)-based mono-azide precursor (3). To a solution of squarate ester (100 mg, 0.704 mmol) in methanol (1 mL), a solution of tri(ethylene glycol) functionalised amine **3i** (147.10 mg, 0.084 mmol) and triethylamine (1 mL) dissolved in methanol (1.5 mL) was added dropwise under a N_2 atmosphere. The resulting mixture was left to stir at room temperature overnight. After removing the solvent under vacuum, purification by column chromatography (eluent: CH_3OH/CH_2Cl_2 2:98 *v/v*) afforded the target squaramide as a yellow oil (211 mgs,94%).

^{1}H NMR (600 MHz, $CDCl_3$) δ = 6.33 (s, 1H, H_1), 4.39 (s, 3H, H_8), 3.72 – 3.63 (m, 8H, $H_{2\text{-}5}$) 3.64 – 3.57 (m, 2H, H_6), 3.42 (t, J = 4.9 Hz, 2H, H_7).

^{13}C NMR (151 MHz, $CDCl_3$) δ = 183.73, 172.56, 77.37, 77.16, 76.95, 70.86, 70.71, 70.32, 70.27, 69.93, 60.59, 50.86, 50.81.

HRMS (ESI +ve) m/z: 307.1013 ($[M+Na]^+$, $C_{11}H_{16}N_4O_5Na$ requires 307.1004).

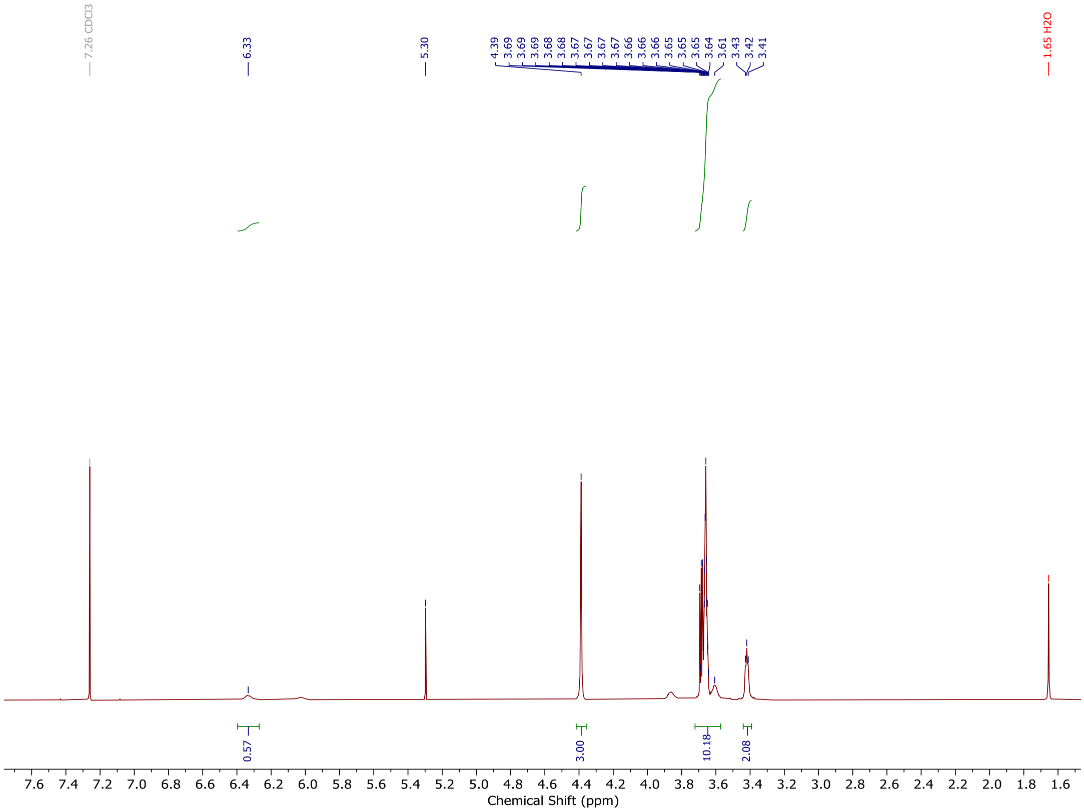

*Figure S1. ^{1}H-NMR spectrum of TEG-based mono-azide axle precursor **3** (500 MHz, $CDCl_3$, 298 K)*

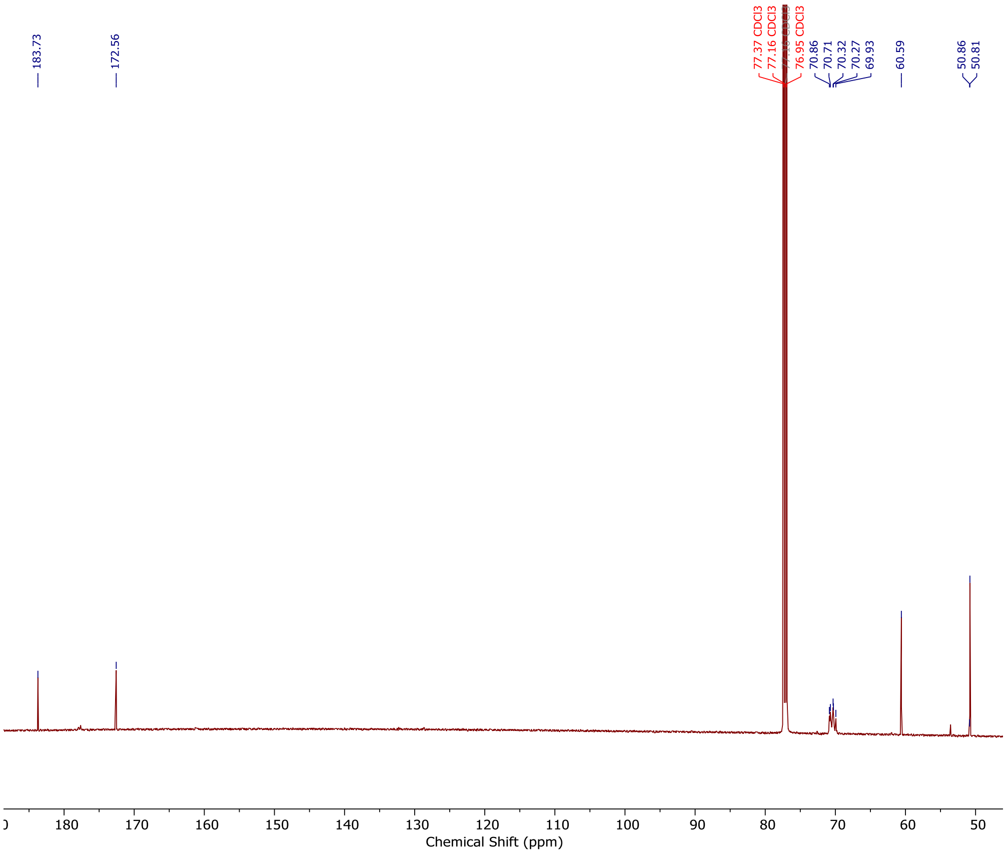

*Figure S2. ^{13}C-NMR spectrum of TEG-based mono-azide axle precursor **3** (500 MHz, $CDCl_3$, 298 K)*

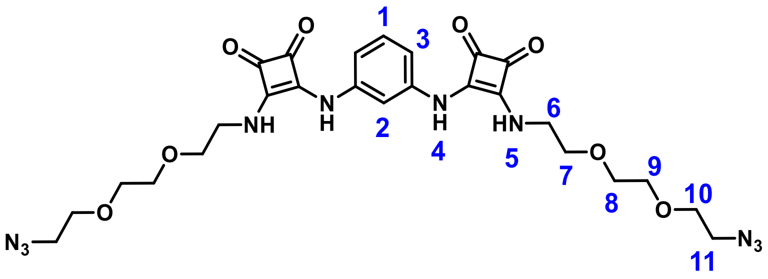

Bis-squaramide aryl spacer-based bis-azide (4). Azide **3** (660 mg, 2.32 mmol), diaminobenzene (119.56 mg, 1.11 mmol) and zinc triflate (40.19, 10 mol%) were dissolved in a solution of toluene/DMF 19:1 (11 mL) under a N_2 atmosphere at room temperature. The resulting mixture was left to stir at 105°C overnight. After removing the solvent under vacuum, purification by column chromatography (eluent: CH_3OH/CH_2Cl_2 4:96 *v/v*) afforded the target bis-squaramide azide precursor **4** as a yellow solid (600 mgs, 89%).

^{1}H NMR (600 MHz, DMSO) δ = 9.74 (s, 2H, H_4), 7.88 (s, 2H, H_5), 7.48 (s, 1H, H_2), 7.28 (t, *J* = 8.1 Hz, 1H, H_1), 7.15 (d, *J* = 8.0 Hz, 2H, H_3), 3.78 (q, *J* = 5.1 Hz, 4H, H_6), 3.64 – 3.58 (m, 16H, H_{7-10}), 3.38 (t, *J* = 4.9 Hz, 4H, H_{11}).

^{13}C NMR (151 MHz, $CDCl_3$) δ = 184.14, 180.19, 169.27, 163.44, 139.98, 130.26, 112.35, 107.41, 69.98, 69.69, 69.59, 69.23, 49.96, 43.57.

HRMS (ESI +ve) m/z: 635.2277 ($[M+Na]^+$, $C_{26}H_{32}N_{10}O_8Na$ requires 635.2297).

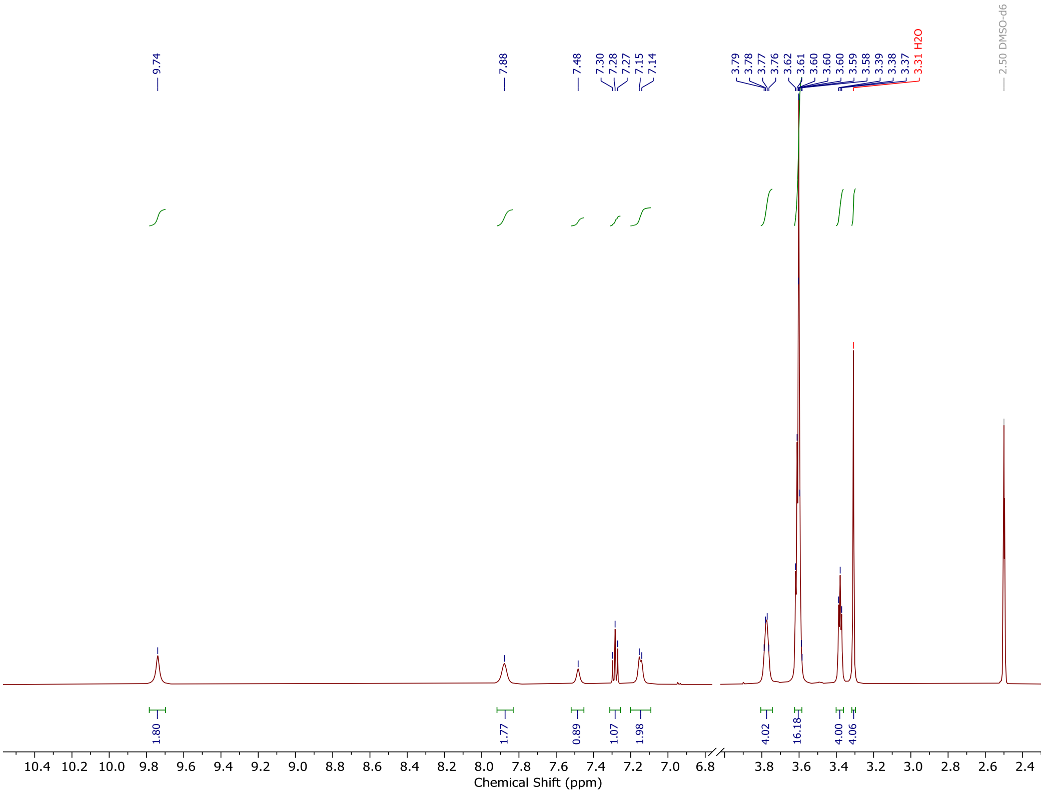

*Figure S3. ^{1}H-NMR spectrum of bis-squaramide aryl spacer-based axle precursor **4** (500 MHz, $CDCl_3$, 298 K)*

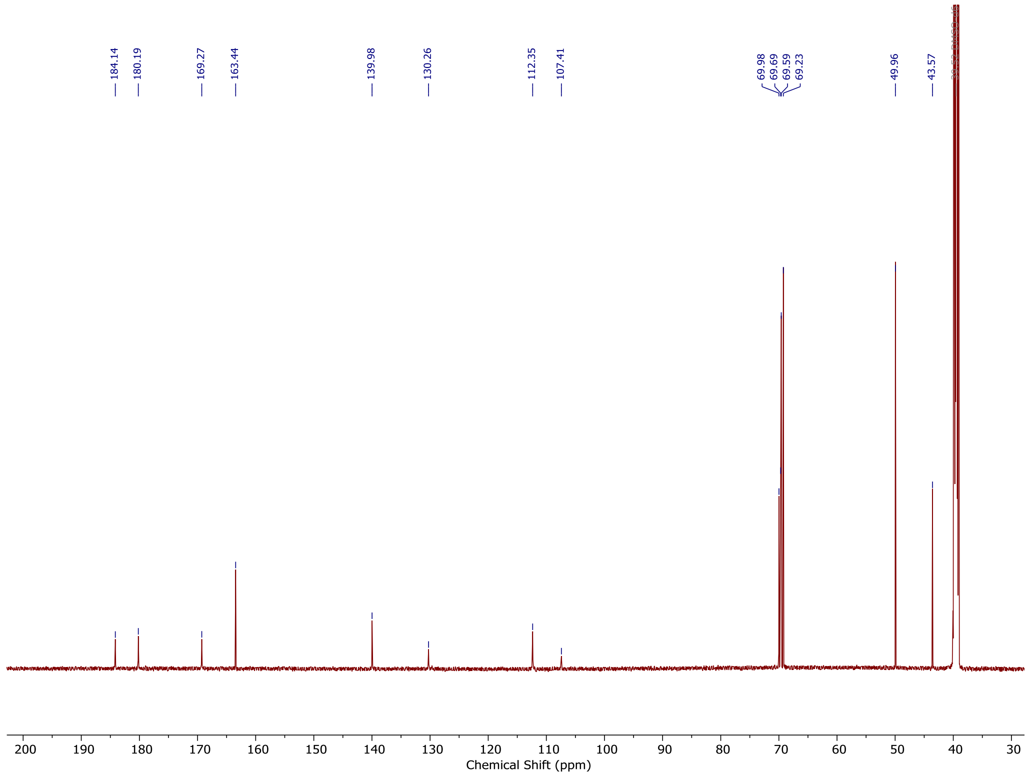

*Figure S4. ^{13}C-NMR spectrum of bis-squaramide aryl spacer-based axle precursor **4** (500 MHz, $CDCl_3$, 298 K)*

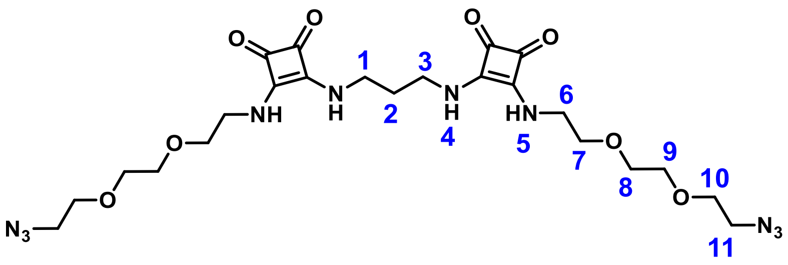

Bis-squaramide alkyl spacer-based bis-azide (5). To a solution of azide **3** (200 mg, 0.704 mmol) in methanol (2 mL), a solution of 1,3 diaminopropane (25 mg, 0.084 mmol) and triethylamine (0.188 mL) dissolved in methanol (2.3 mL) was added dropwise under a N_2 atmosphere. The resulting mixture was left to stir at room temperature overnight. After removing the solvent under vacuum, purification by column chromatography (eluent: CH_3OH/CH_2Cl_2 7:93 *v/v*) afforded the target bis-squaramide azide precursor **5** as a white solid (130 mgs, 67%).

^{1}H NMR (600 MHz, DMSO) δ = 7.45 (s, 4H, $H_{4,5}$), 3.66 (s, 4H, H_6), 3.62 – 3.52 (m, 20H, $H_{1,3,7\text{-}10}$), 3.41 – 3.36 (m, 4H, H_{11}), 1.80 (p, J = 6.9 Hz, 2H, H_2).

^{13}C NMR (151 MHz, DMSO) δ = 182.47, 167.70, 70.17, 69.66, 69.58, 69.22, 49.96, 43.14, 40.62, 40.05, 32.39.

HRMS (ESI +ve) m/z: 601.2435 ([M+Na]$^+$, $C_{23}H_{24}$ $N_{10}O_8Na$ requires 601.2453).

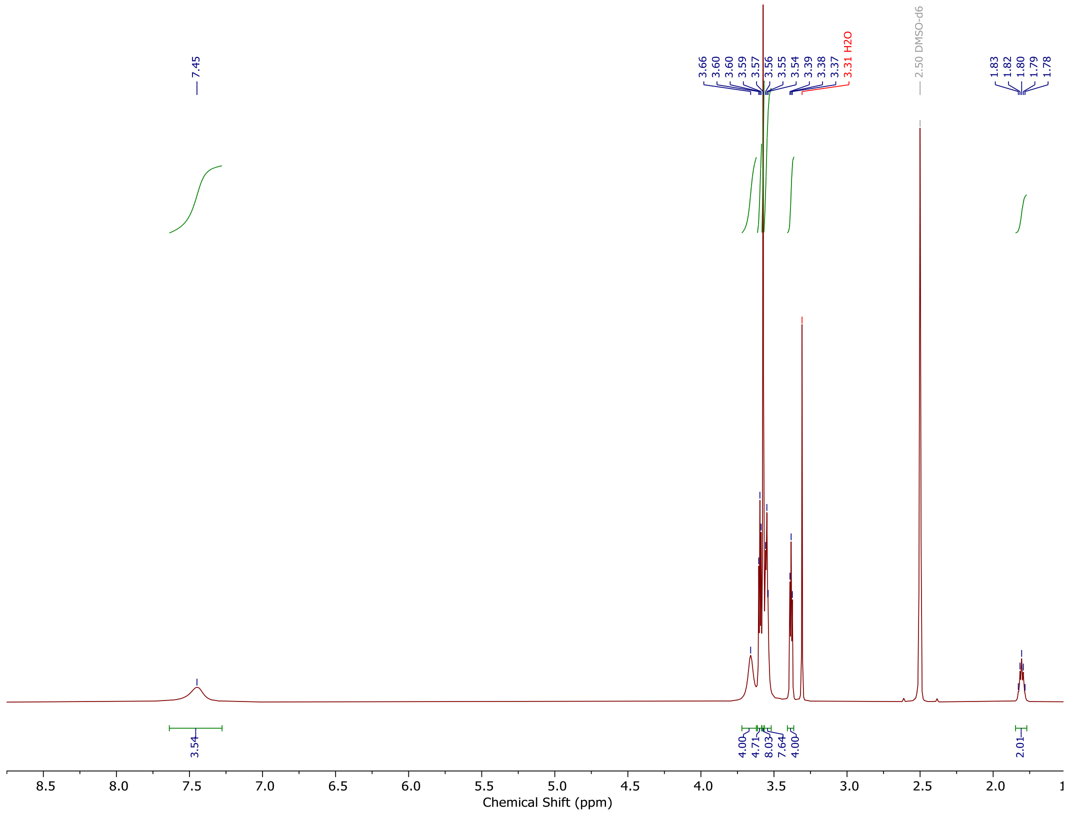

*Figure S5. ^{1}H-NMR spectrum of bis-squaramide alkyl spacer-based axle precursor **5** (500 MHz, $CDCl_3$, 298 K)*

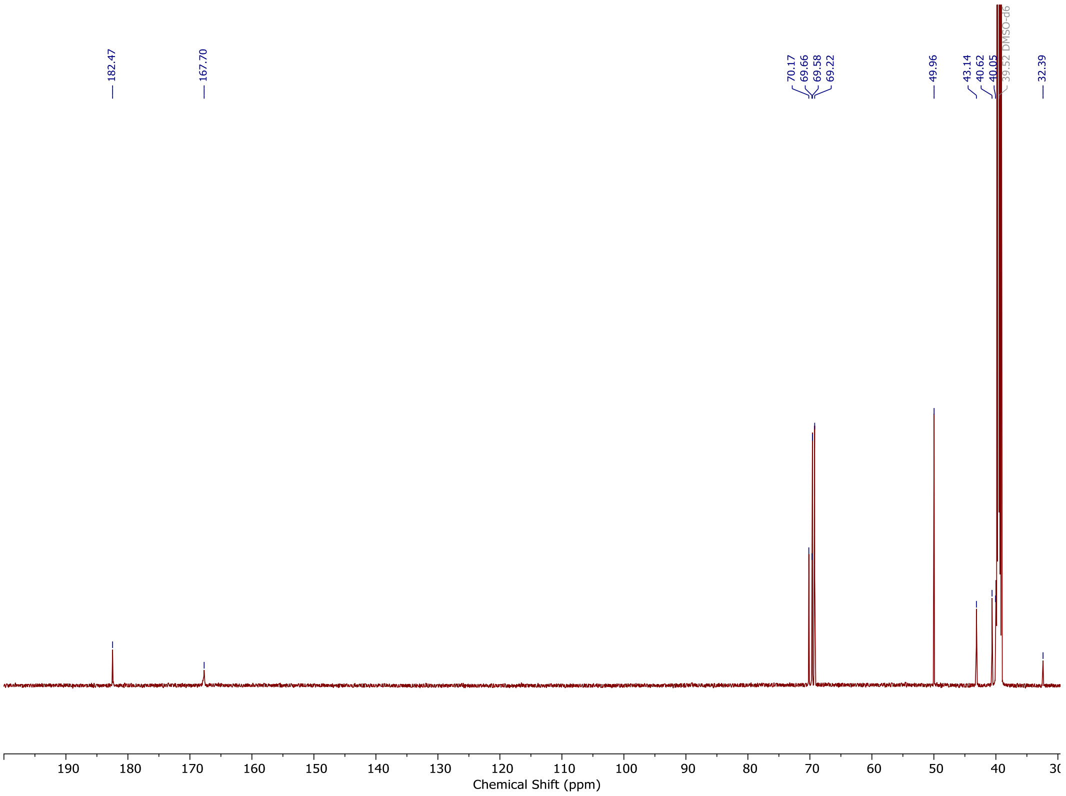

*Figure S6. ^{13}C-NMR spectrum of bis-squaramide alkyl spacer-based axle precursor **5** (500 MHz, $CDCl_3$, 298 K)*

General procedure 2: Synthesis of mono-squaramide based azide axle precursor 6

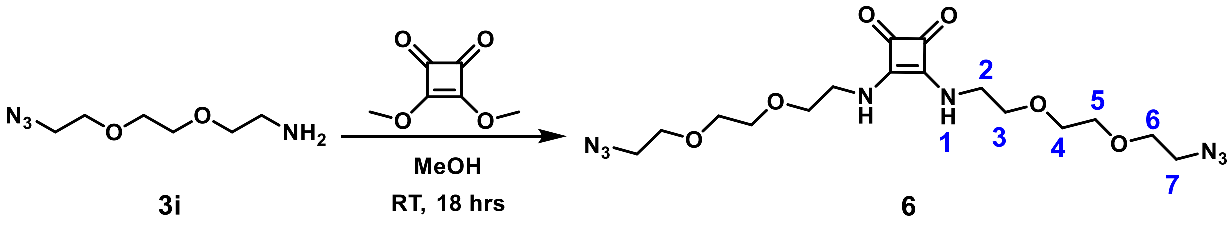

Squaramide tri(ethylene glycol)-based bis-azide axle precursor (6) To a solution of squarate ester (75 mg, 0.53 mmol) in methanol (1 mL), a solution of tri(ethylene glycol) functionalised amine **3i** (193.07 mg, 1.1083 mmol)) dissolved in methanol (1 mL) was added dropwise under a N_2 atmosphere. The resulting mixture was left to stir at room temperature overnight. After removing the solvent under vacuum, purification by column chromatography (eluent: CH_3OH/CH_2Cl_2 3:97 *v/v*) afforded the target squaramide as a yellow oil (211 mgs,94%).

^{1}H NMR (600 MHz, $CDCl_3$) δ = 6.42 (t, J = 6.1 Hz, 2H, H_1), 3.83 (q, J = 5.3 Hz, 4H, H_2), 3.67 (q, J = 5.5 Hz, 16H, H_{3-6}), 3.40 (t, J = 4.9 Hz, 4H, H_7).

^{13}C NMR (151 MHz, CDCl3) δ = 183.4, 168.1, 77.2, 77.0, 76.8, 70.6, 70.5, 70.5, 70.2, 70.1, 70.0, 69.9, 50.7, 44.0, 40.9, 39.3.

HRMS (ESI +ve) m/z: 427.2041 ($[M+H]^+$, $C_{16}H_{26}N_8O_6$ requires 427.2048).

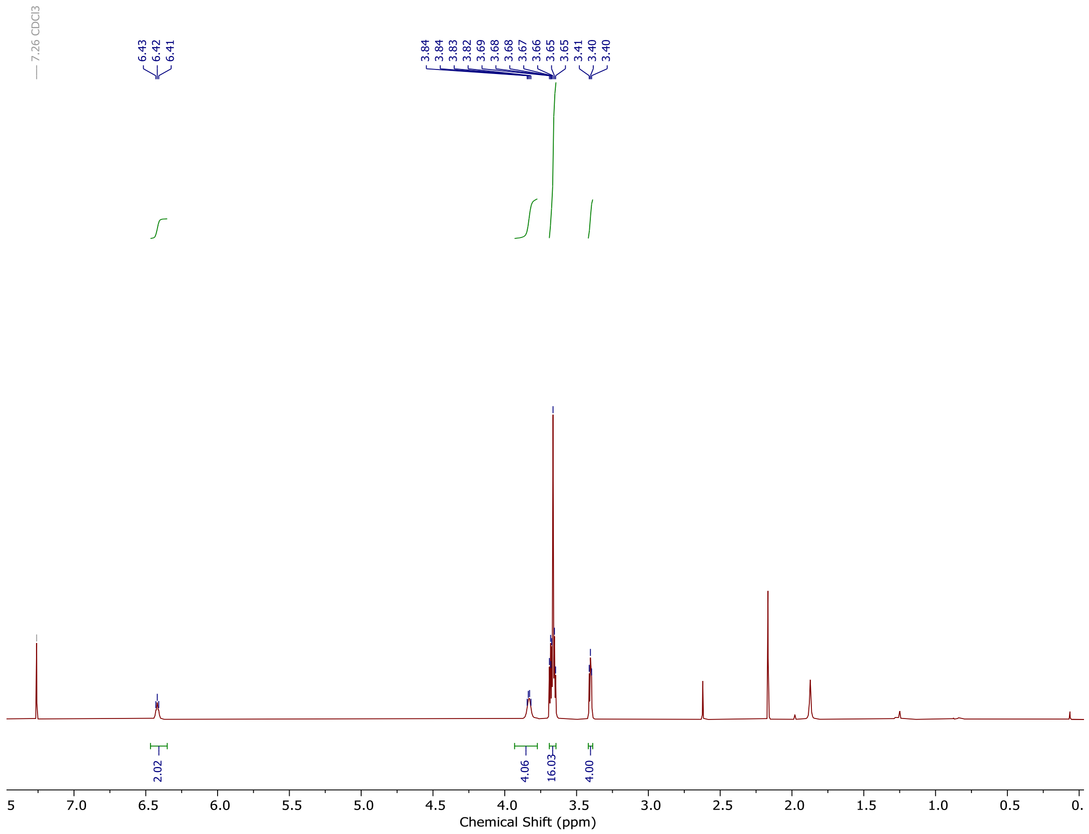

*Figure S7. ^{1}H-NMR spectrum of TEG-based bis-azide axle precursor **6** (500 MHz, $CDCl_3$, 298 K)*

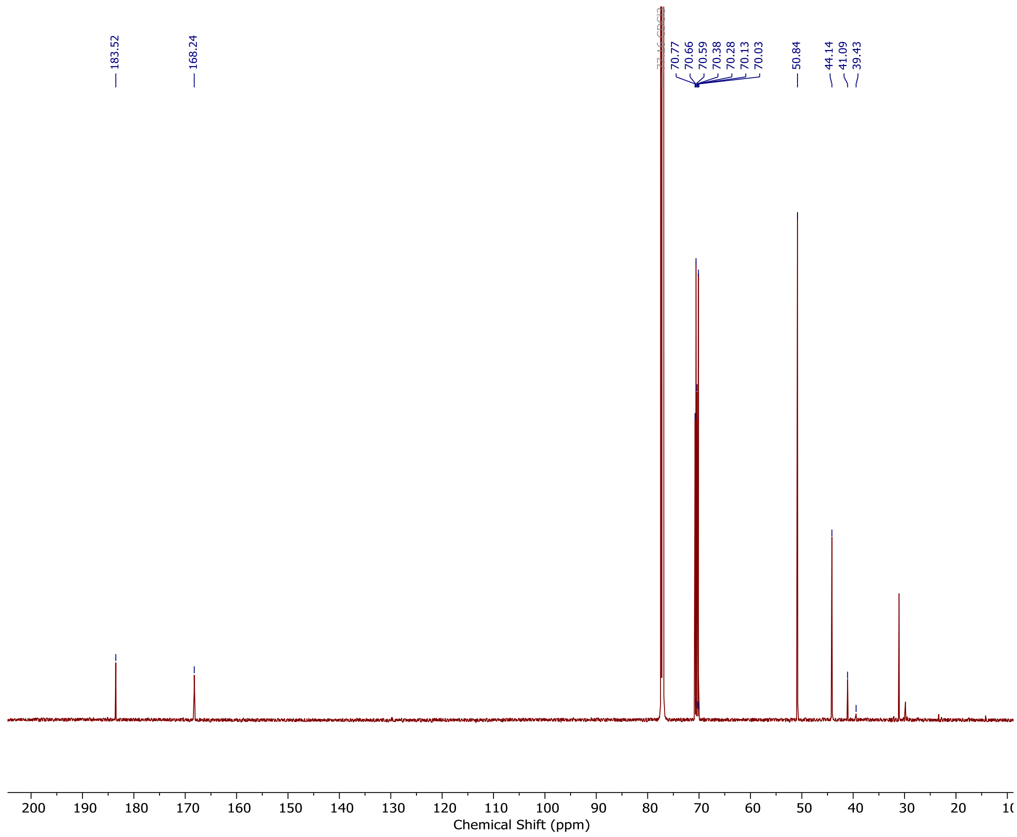

*Figure S8. ^{13}C-NMR spectrum of TEG-based bis-azide axle precursor **6** (500 MHz, $CDCl_3$, 298 K)*

General procedure 3: Synthesis of [2]rotaxanes using active metal template methodology

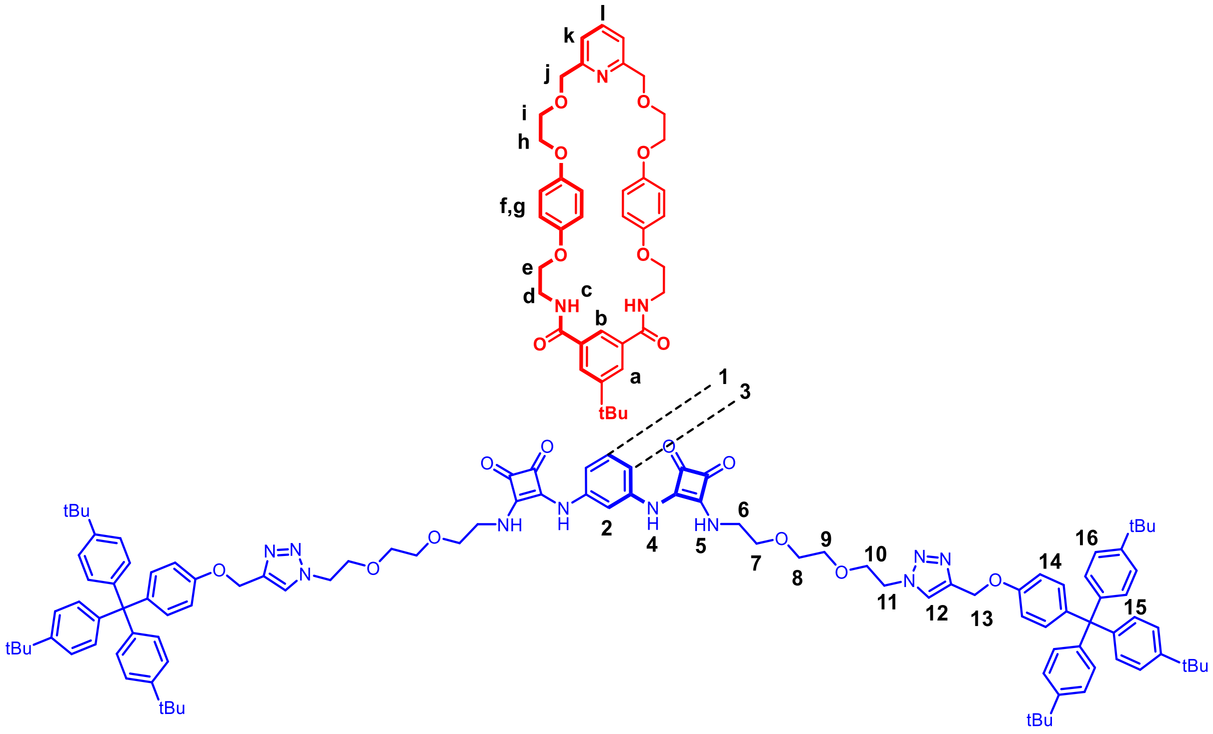

[2]rotaxane (7). Macrocycle **1** (13.68 mg, 0.02 mmol) and $[Cu(CH_3CN)_4]PF_6$ (7.46mg, 0.02 mmol) were dissolved in dry, degassed dichloroethane (1.0 mL) and stirred for 30 minutes at room temperature. A solution of bis-azide **4** (36.76 mg, 0.06 mmol) and terphenyl stopper alkyne **2** (65.16 mg, 0.12 mmol) in dichloroethane (1.0 mL) was subsequently added dropwise to the Cu-complexed macrocycle solution. The reaction mixture was stirred at 80°C for 72 hours, following which it was cooled to room temperature and diluted with CH_2Cl_2 (40 mL). The organic layer was washed with EDTA/NH_4OH (2 × 25 mL) and H_2O (2 × 25 mL), dried over $MgSO_4$, filtered and concentrated under vacuum. The crude was purified by preparative TLC in 60:35:5 CH_2Cl_2/EtOAc/MeOH to afford [2]rotaxane **7** as a white solid (5.3 mg, 15%).

^{1}H NMR (400 MHz, $CDCl_3$) δ = 9.52 (s, 2H, H_5), 8.38 (s, 1H, H_b), 8.27 (s, 2H, H_a), 7.73 (dd, J = 14.7, 7.1 Hz, 3H, $H_{c,l}$), 7.48 (s, 2H, H_{12}), 7.40 (s, 1H, H_1), 7.26 (s, 2H, H_3), 7.20 (d, J = 8.4 Hz, 12H, H_{16}), 7.07 (dd, J = 18.7, 8.7 Hz, 19H, $H_{k,15,2}$), 6.79 (d, J = 8.4 Hz, 4H, H_{14}), 6.69 (s, 2H, H_4), 6.52 – 6.43 (m, 8H, $H_{f,g}$), 5.11 (s, 4H, H_{13}), 4.67 (s, 4H, H_j), 4.43 (d, J = 5.4 Hz, 4H, H_{11}), 3.93 (s, 4H, H_e), 3.85 (s, 8H, $H_{h,7}$), 3.77 (t, J = 5.1 Hz, 4H, H_{10}), 3.65 (d, J = 14.2 Hz, 8H, $H_{d,9}$), 3.46 (s, 4H, H_i), 3.40 (s, 8H, $H_{8,6}$), 1.38 (s, 9H, H_{mactBu}), 1.28 (s, 54H, H_{axtBu}).

^{13}C NMR (151 MHz, Acetone) δ 181.55, 167.61, 158.62, 157.65, 153.76, 152.68, 149.35, 145.40, 144.36, 141.21, 140.76, 139.53, 135.51, 133.64, 133.02, 132.18, 131.61, 131.04, 129.80, 128.94, 125.55, 125.24, 123.30, 122.95, 116.04, 115.85, 114.50, 113.80, 74.31, 71.30, 71.03, 70.97, 70.57, 70.13, 68.49, 68.17, 67.51, 64.09, 62.46, 55.65, 50.95, 45.05, 44.92, 40.86, 40.73, 39.85, 35.74, 35.03, 31.83, 30.49, 30.37, 30.24, 30.11, 29.98, 29.85, 29.73, 29.60.

HRMS (ESI +ve) m/z: 2381.2749 ($[M+H]^+$, $C_{145}H_{169}N_{13}O_{18}$ requires 2381.2781).

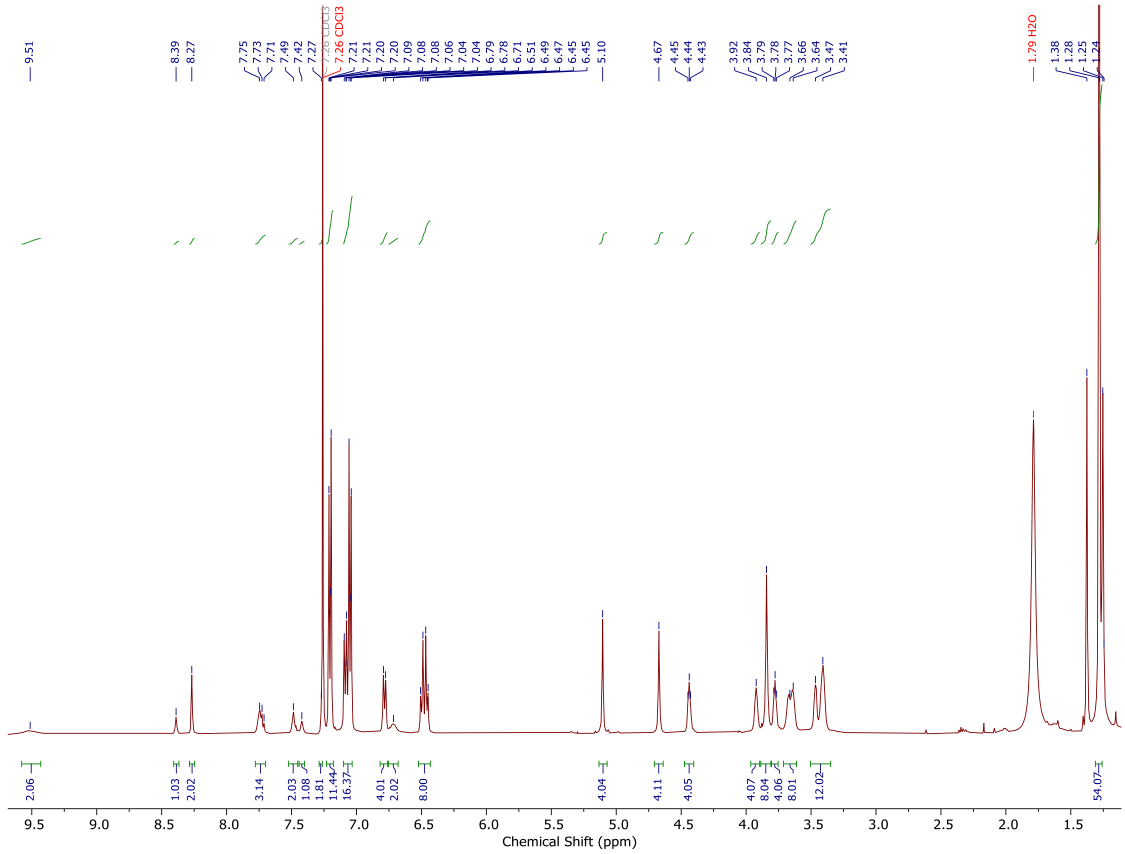

*Figure S9. ^{1}H-NMR spectrum of [2]rotaxane **7** (500 MHz, $CDCl_3$, 298 K)*

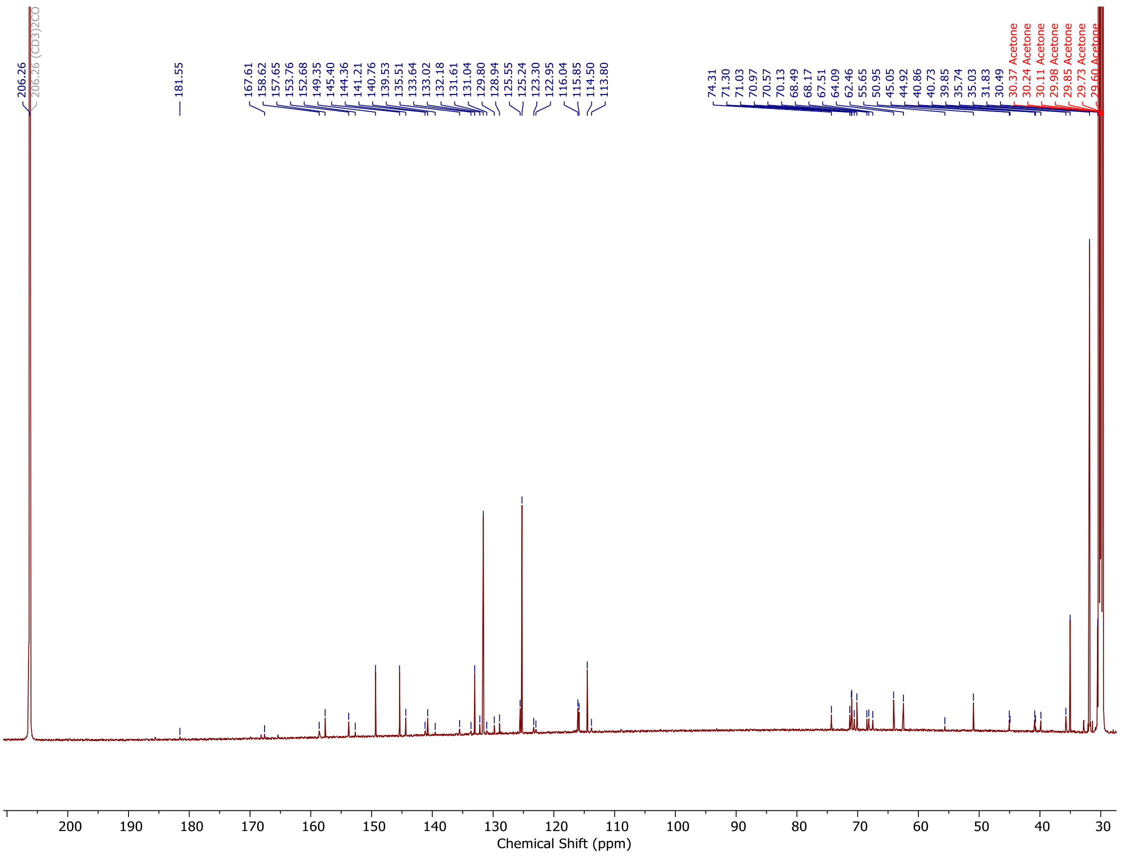

*Figure S10. ^{13}C-NMR spectrum of [2]rotaxane **7** (600 MHz, $CDCl_3$, 298 K)*

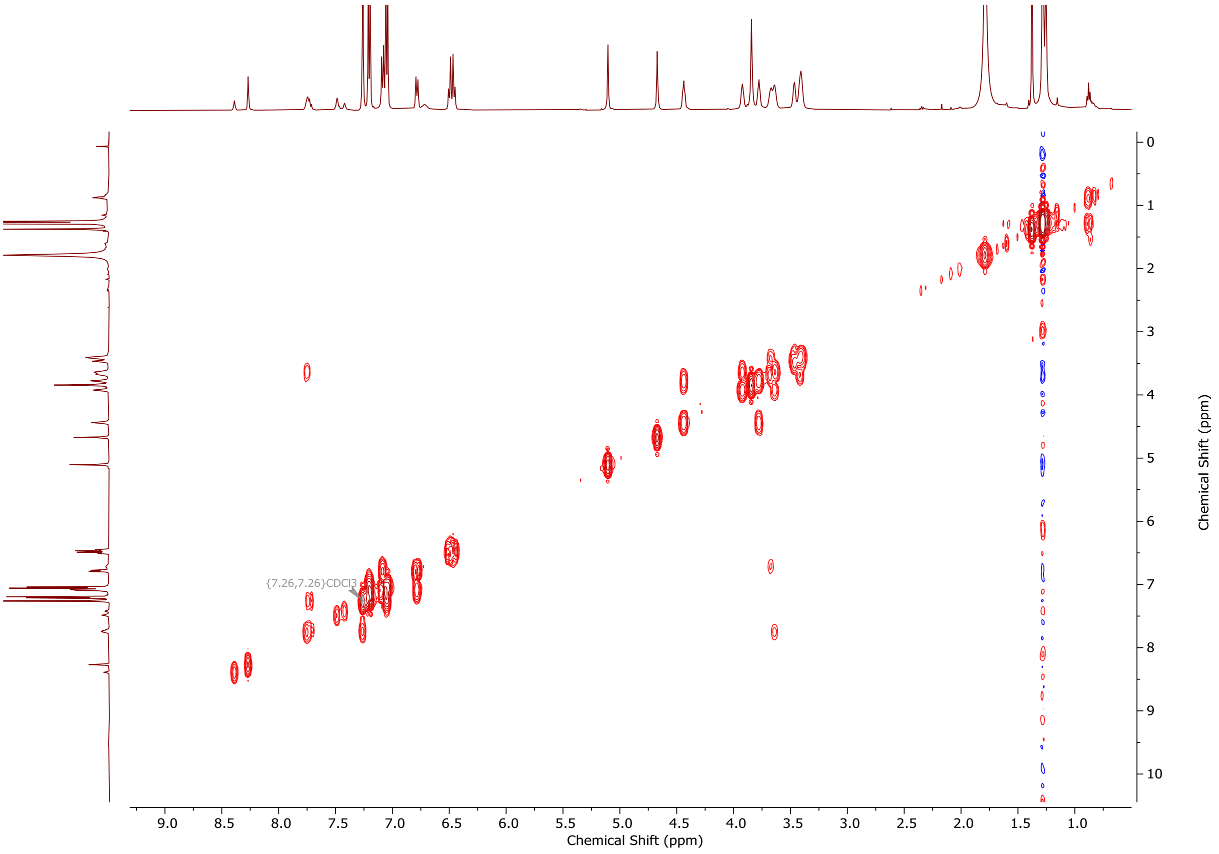

*Figure S11. ^1H-^1H COSY NMR spectrum of [2]rotaxane **7** (500 MHz, $CDCl_3$, 298 K)*

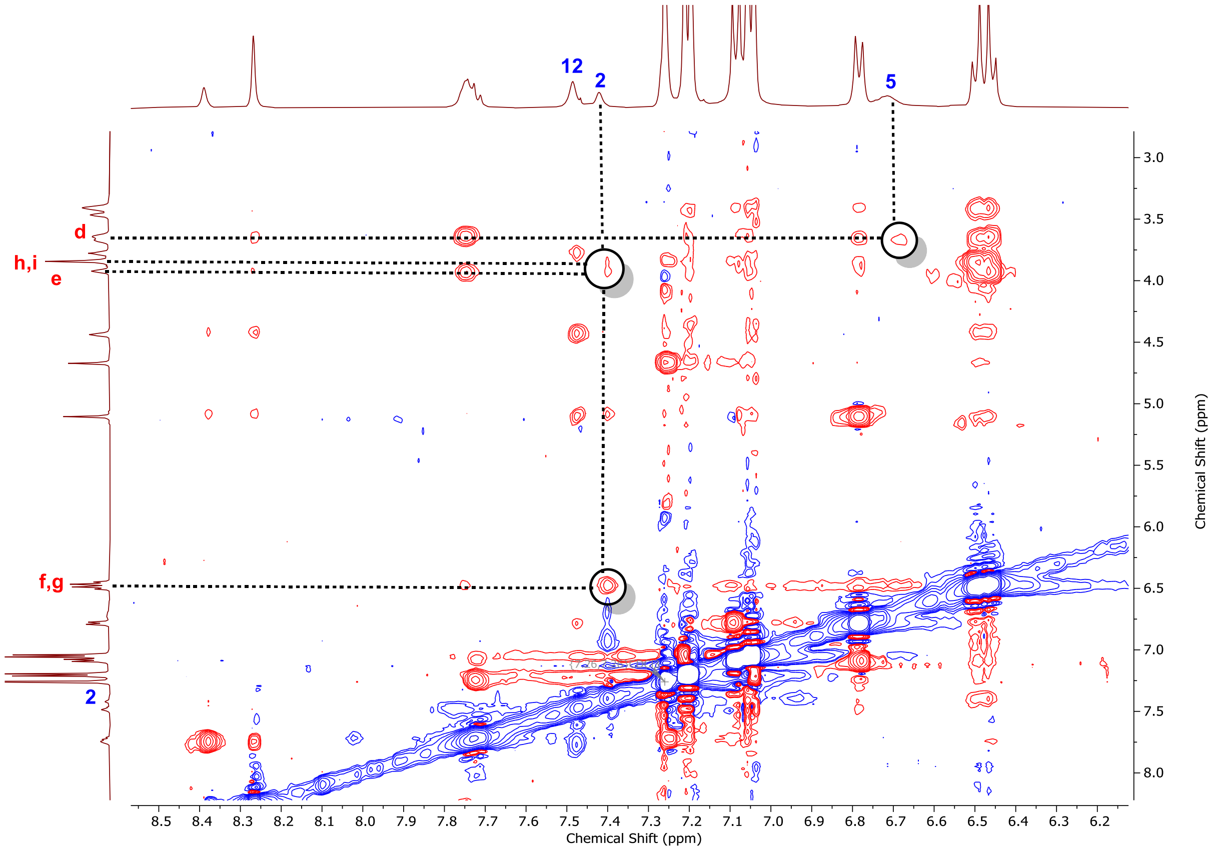

*Figure S12. ^{1}H-^{1}H ROESY NMR spectrum of [2]rotaxane **7** (500 MHz, $CDCl_3$, 298 K)*

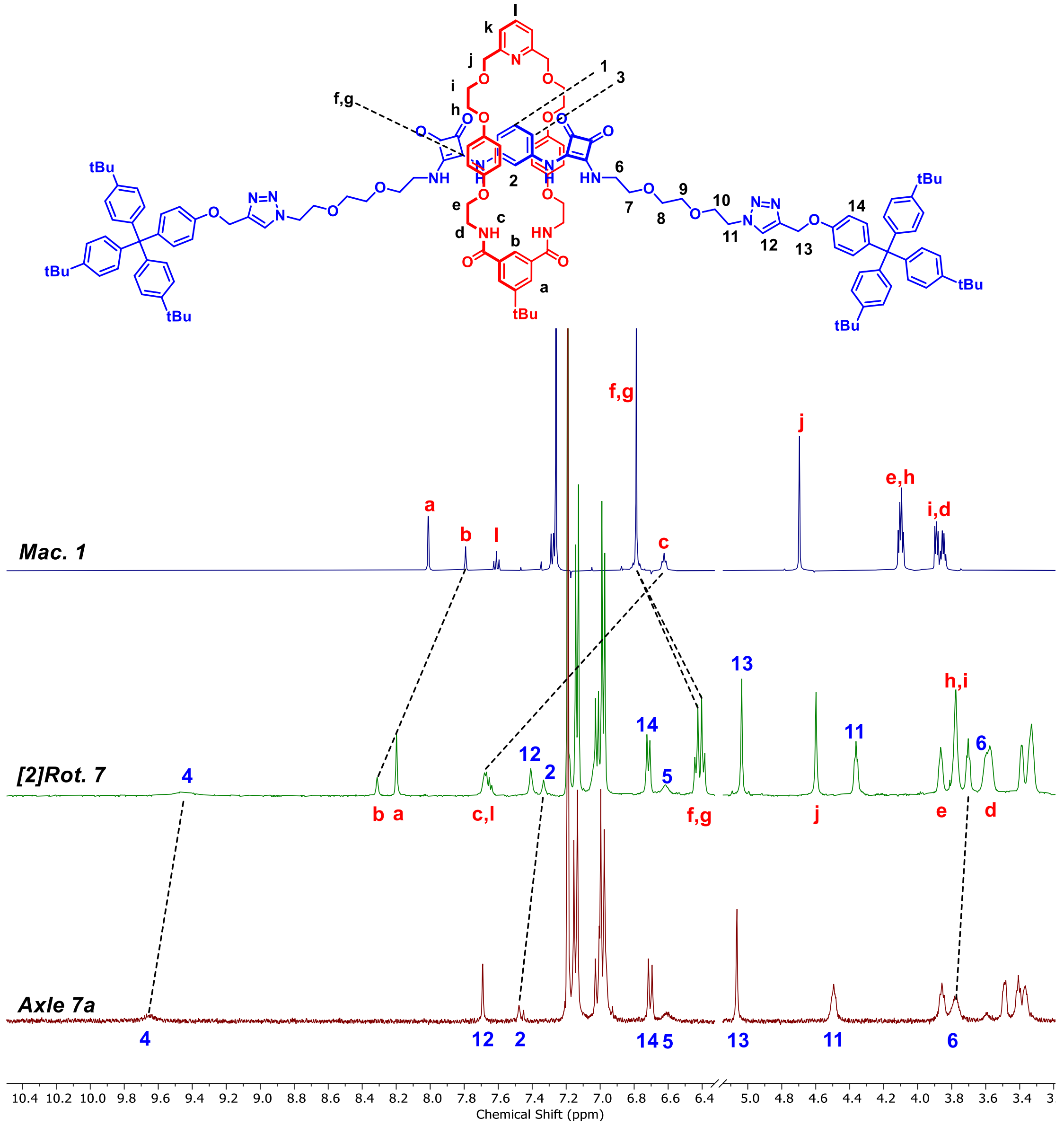

*Figure S13. Stacked ^{1}H NMR spectra of Macrocycle **1** (top) and [2]rotaxane **7** (middle) and axle **7a** (bottom) (500 MHz, $CDCl_3$, 298 K).*

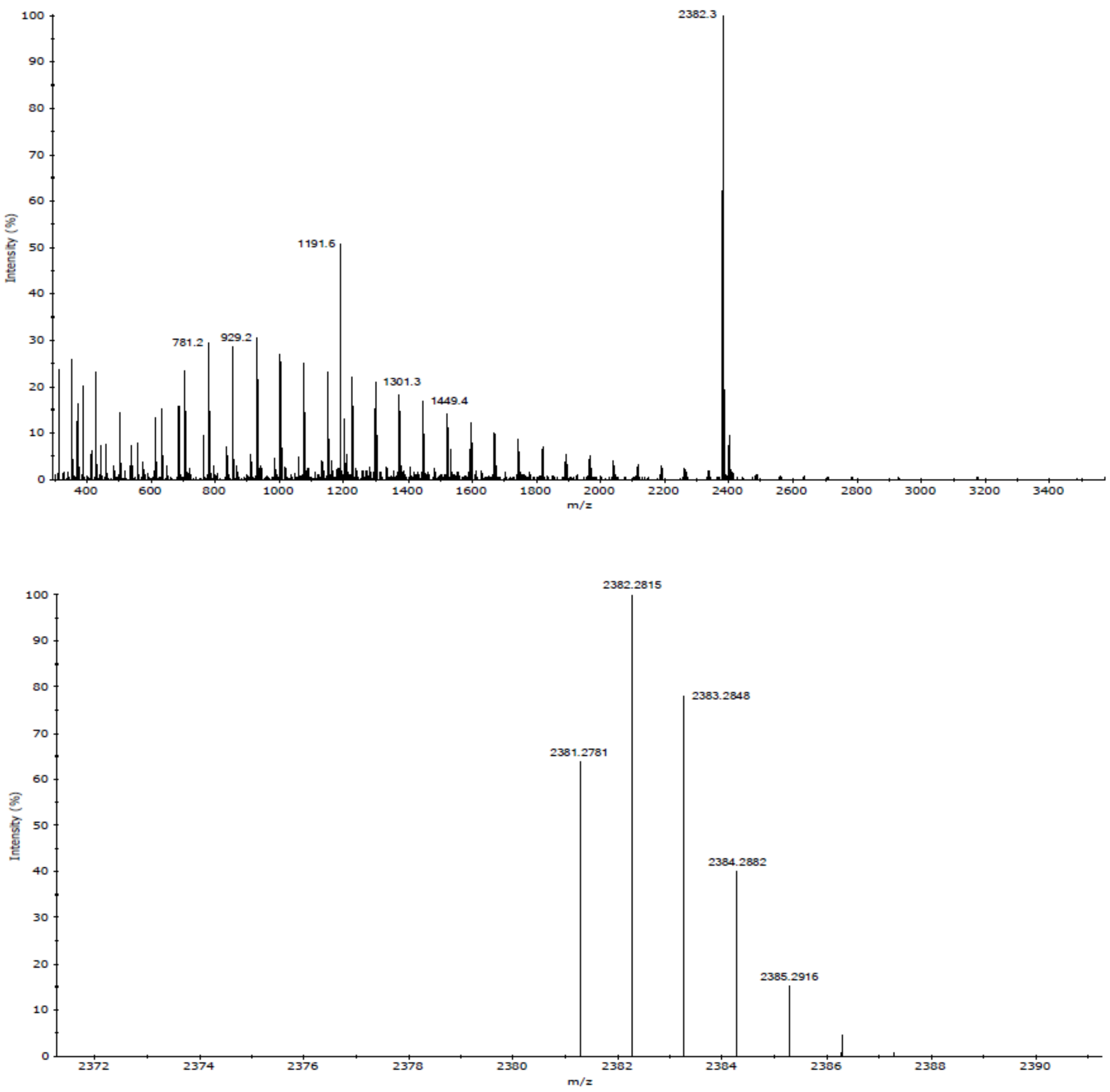

*Figure S14. High-resolution mass spectrum (ESI +ve) of **[2]rotaxane 7** (top: experimental; bottom: expanded theoretical).*

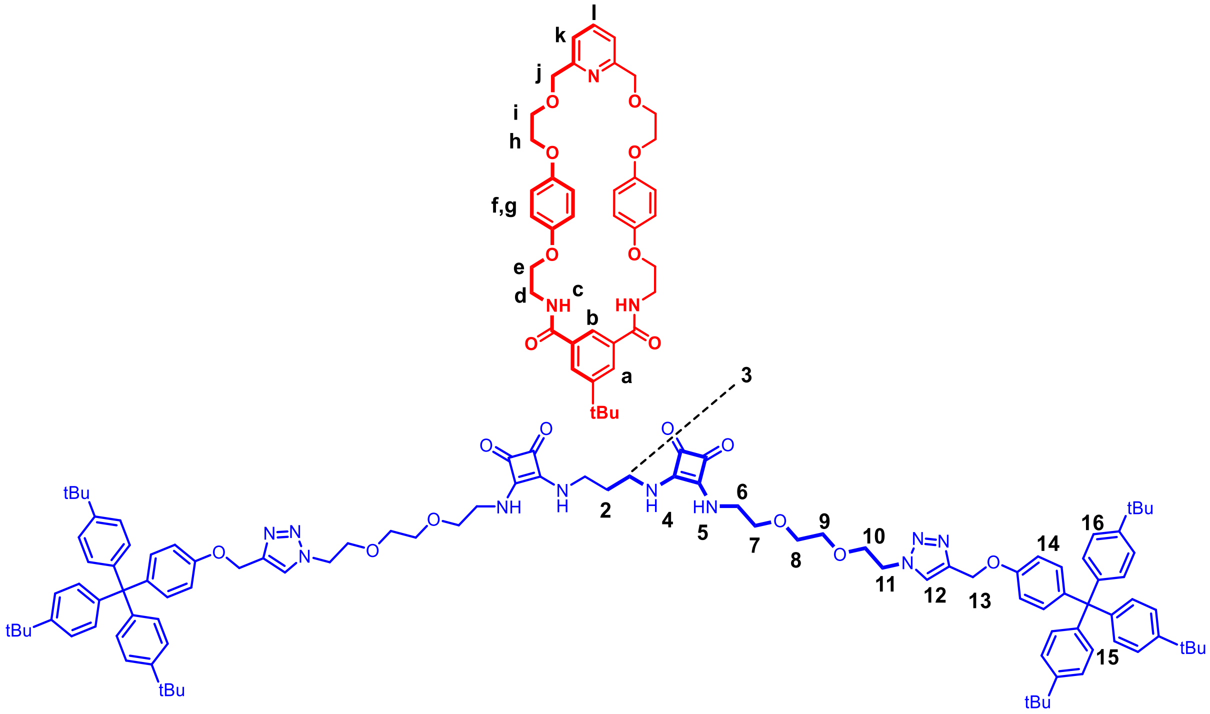

[2]rotaxane (8). Macrocycle **1** (13.50 mg, 0.019 mmol) and $[Cu(CH_3CN)_4]PF_6$ (7.86mg, 0.019 mmol) were dissolved in dry, degassed dichloroethane (1.0 mL) and stirred for 30 minutes at room temperature. A solution of bis-azide **5** (34.27 mg, 0.06 mmol) and terphenyl stopper alkyne **2** (64.30 mg, 0.118 mmol) in dichloroethane (1.0 mL) was subsequently added dropwise to the Cu-complexed macrocycle solution. The reaction mixture was stirred at 80°C for 72 hours, following which it was cooled to room temperature and diluted with CH_2Cl_2 (40 mL). The organic layer was washed with EDTA/NH_4OH (2 × 25 mL) and H_2O (2 × 25 mL), dried over $MgSO_4$, filtered and concentrated under vacuum. The crude was purified by preparative TLC in 70:30:6 CH_2Cl_2/EtOAc/MeOH to afford [2]rotaxane **8** as a white solid (5.6 mg, 12%).

^{1}H NMR (500 MHz, $CDCl_3$) δ = 8.36 (s, 1H, H_b), 8.21 (d, J = 1.4 Hz, 2H, H_a), 7.85 (s, 2H, H_c), 7.69 (t, J = 7.9 Hz, 1H, H_l), 7.65 (s, 2H, H_{12}), 7.26 (s, 2H, H_k), 7.21 (d, J = 8.6 Hz, 12H, H_{16}), 7.14 – 7.02 (m, 16H, H_{15}), 6.88 (s, 2H, H_4), 6.85 – 6.78 (m, 4H, H_{14}), 6.71 – 6.55 (m, 8H, $H_{f,g}$), 5.08 (s, 4H H_{13}), 4.67 (s, 4H, H_j), 4.44 (t, J = 5.0 Hz, 4H, H_{11}), 4.03 (p, J = 4.1 Hz, 8H, $H_{e,h}$), 3.90 (d, J = 4.4 Hz, 4H, H_i), 3.78 (t, J = 5.0 Hz, 4H, H_{10}), 3.68 (s, 8H, $H_{d,7}$), 3.50 – 3.43 (m, 12H, $H_{6,8,9}$), 3.32 (s, 4H, $H_{1,3}$), 1.57 (s, 2H, H_2), 1.34 (s, 9H, H_{mactBu}), 1.29 (s, 54H, H_{axtBu}).

^{13}C NMR (151 MHz, Acetone) δ 184.28, 168.16, 167.73, 158.70, 157.67, 154.01, 153.88, 152.71, 149.34, 145.42, 144.35, 140.72, 138.60, 135.65, 133.65, 133.00, 132.18, 131.61, 129.80, 128.73, 125.55, 125.25, 123.59, 121.91, 116.41, 116.39, 114.50, 74.54, 71.48, 71.07, 71.03, 70.53, 70.17, 68.74, 68.50, 67.68, 64.09, 62.46, 50.93, 44.76, 44.64, 41.74, 40.64, 40.51, 39.85, 30.52, 30.49, 30.37, 30.24, 30.12, 29.99, 29.86, 29.73, 29.60.

HRMS (ESI +ve) m/z: 2349.2976 ($[M+H]^+$, $C_{142}H_{171}N_{13}O_{18}$ requires 2349.3005).

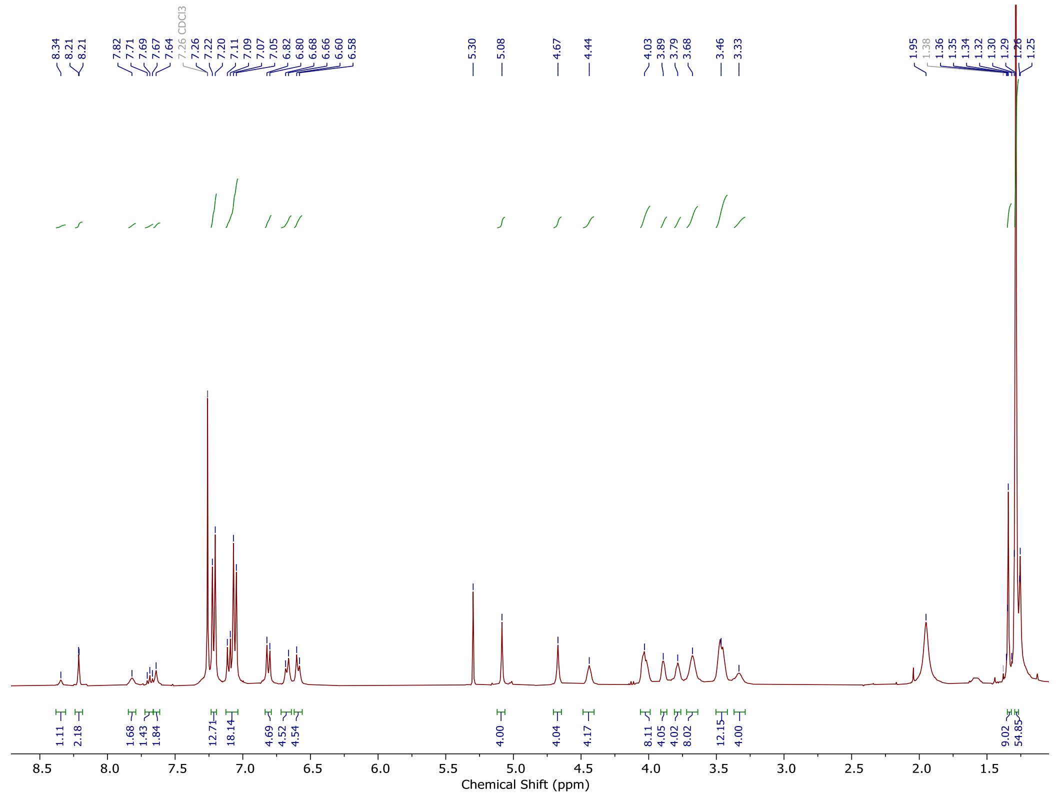

*Figure S15. ^{1}H-NMR spectrum of [2]rotaxane **8** (500 MHz, $CDCl_3$, 298 K)*

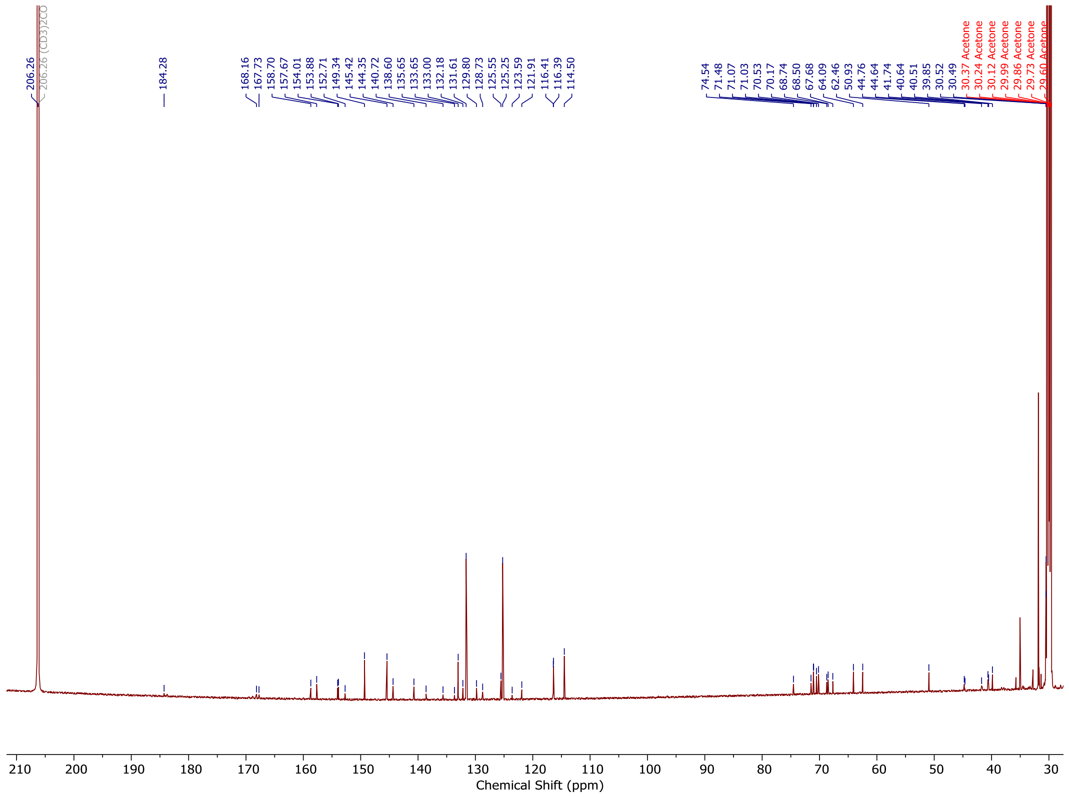

*Figure S16. ^{13}C-NMR spectrum of [2]rotaxane **8** (600 MHz, $(CD_3)_2CO$, 298 K)*

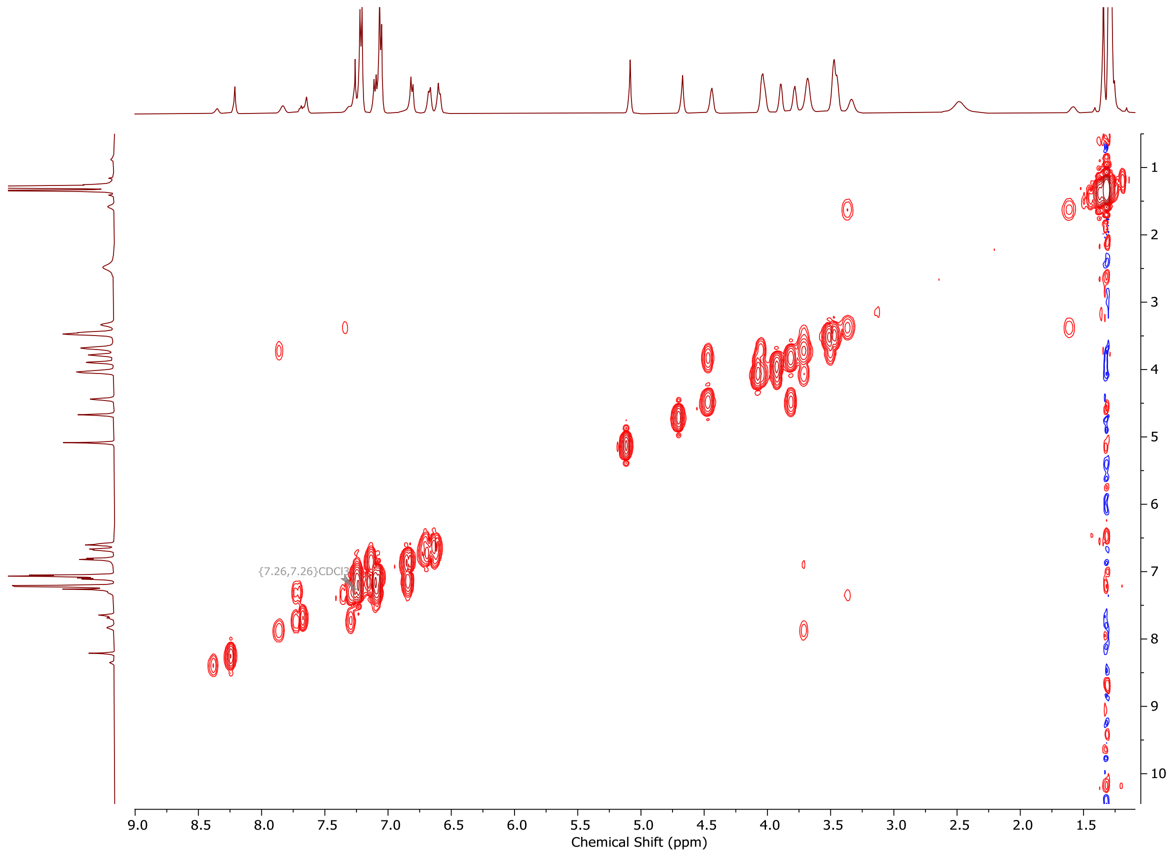

*Figure S17. ^{1}H-^{1}H COSY NMR spectrum of [2]rotaxane **8** (500 MHz, $CDCl_3$, 298 K)*

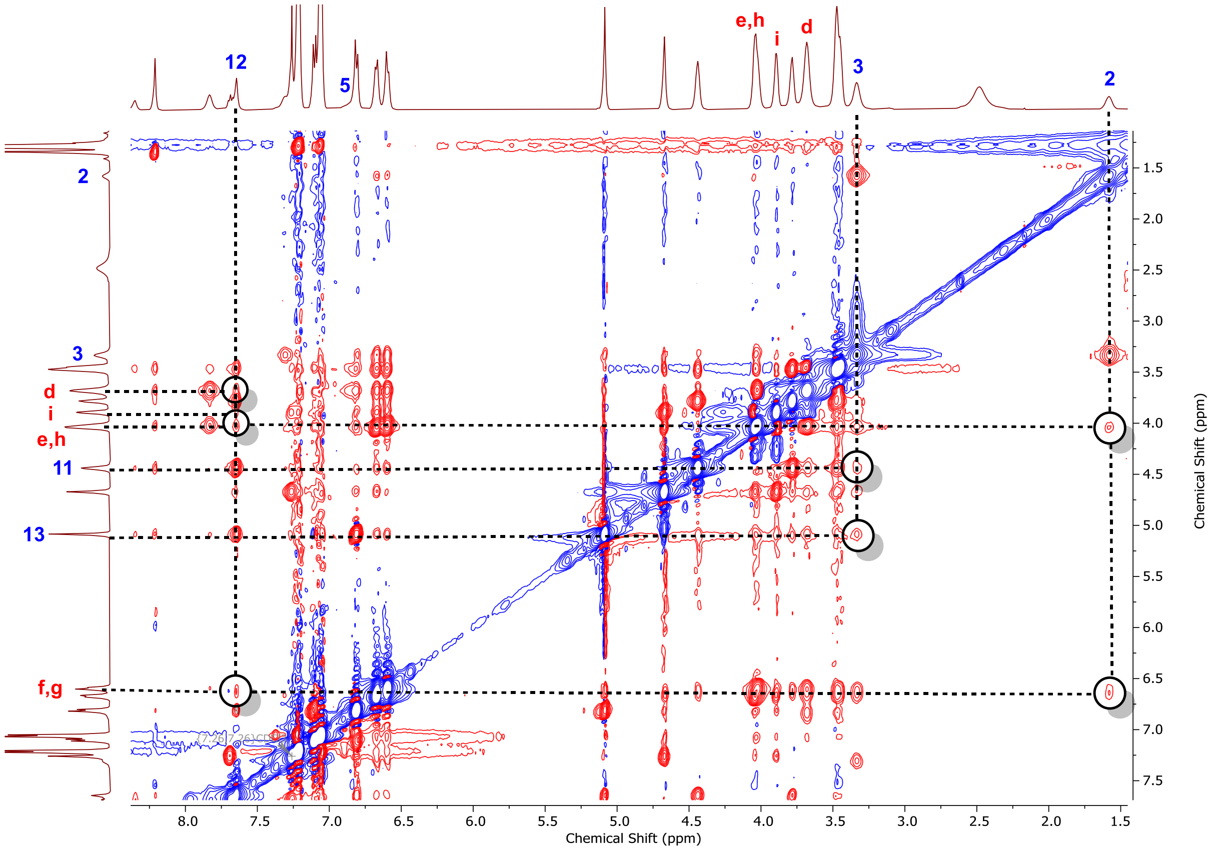

*Figure S18. ¹H-¹H ROESY NMR spectrum of [2]rotaxane **8** (500 MHz, $CDCl_3$, 298 K)*

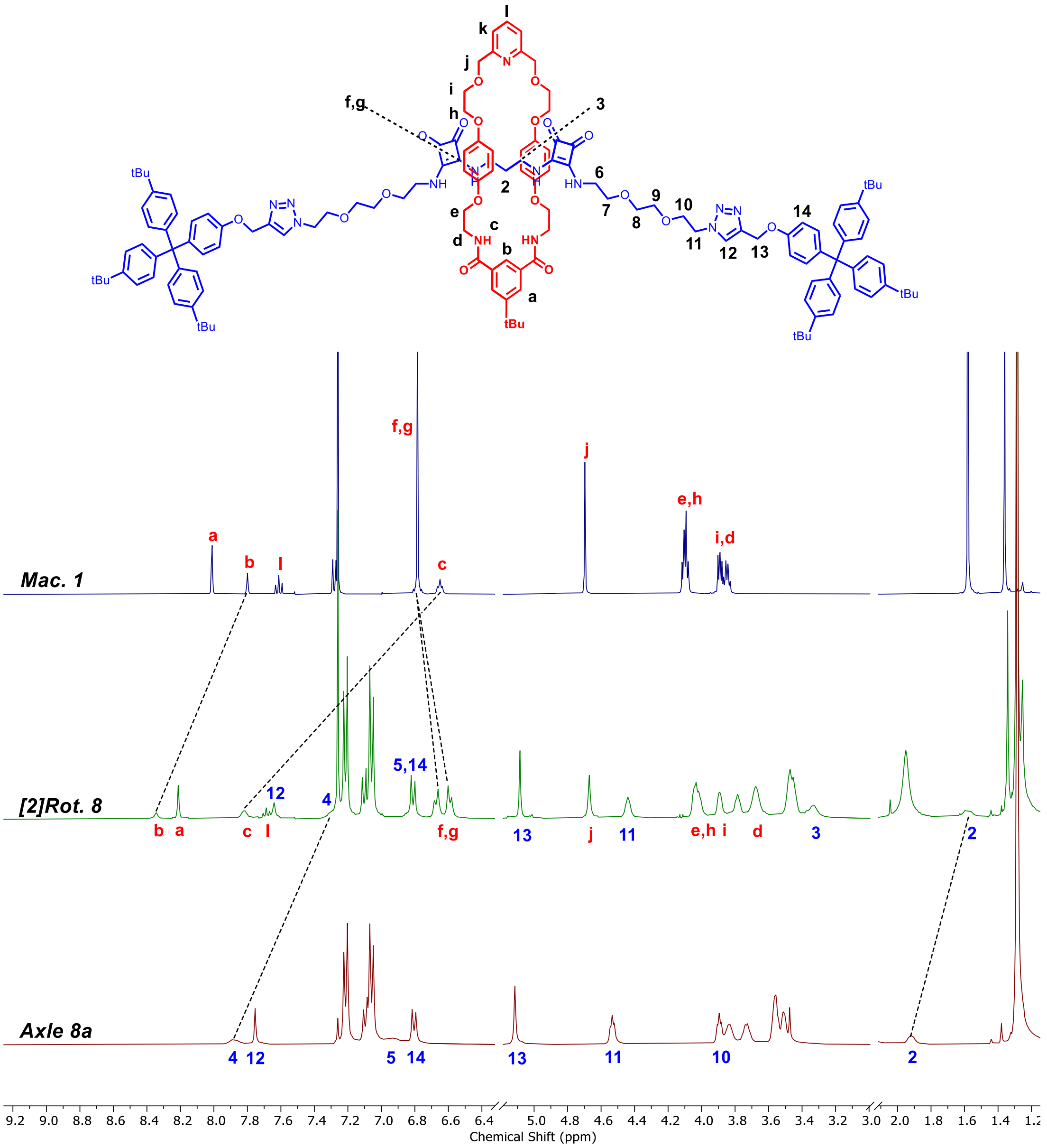

*Figure S19. Stacked ¹H NMR spectra of Macrocycle **1** (top) and [2]rotaxane **8** (middle) and axle **8a** (bottom) (500 MHz, $CDCl_3$, 298 K).*

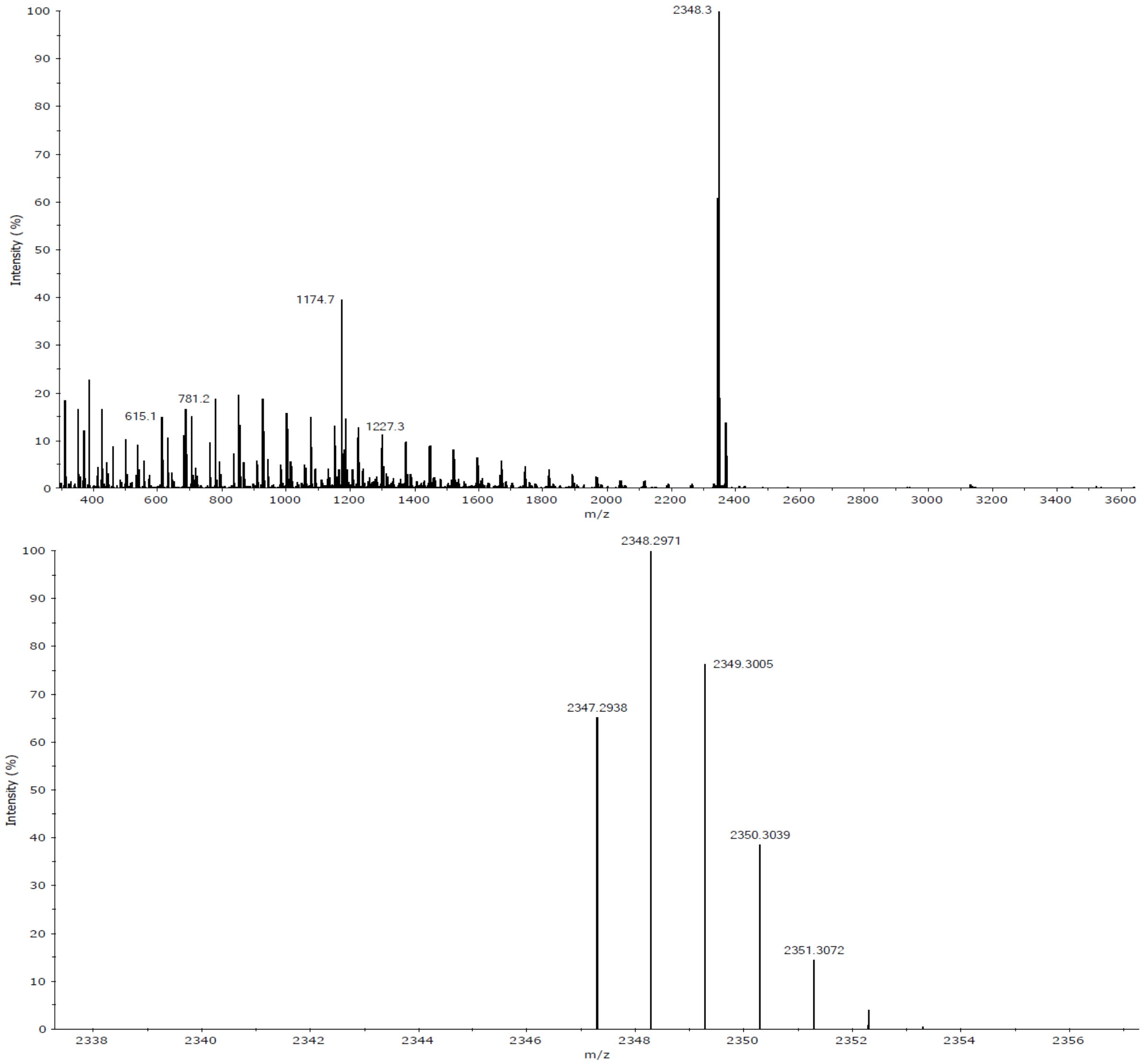

*Figure S20. High-resolution mass spectrum (ESI +ve) of [2]rotaxane **8** (top: experimental; bottom: expanded theoretical).*

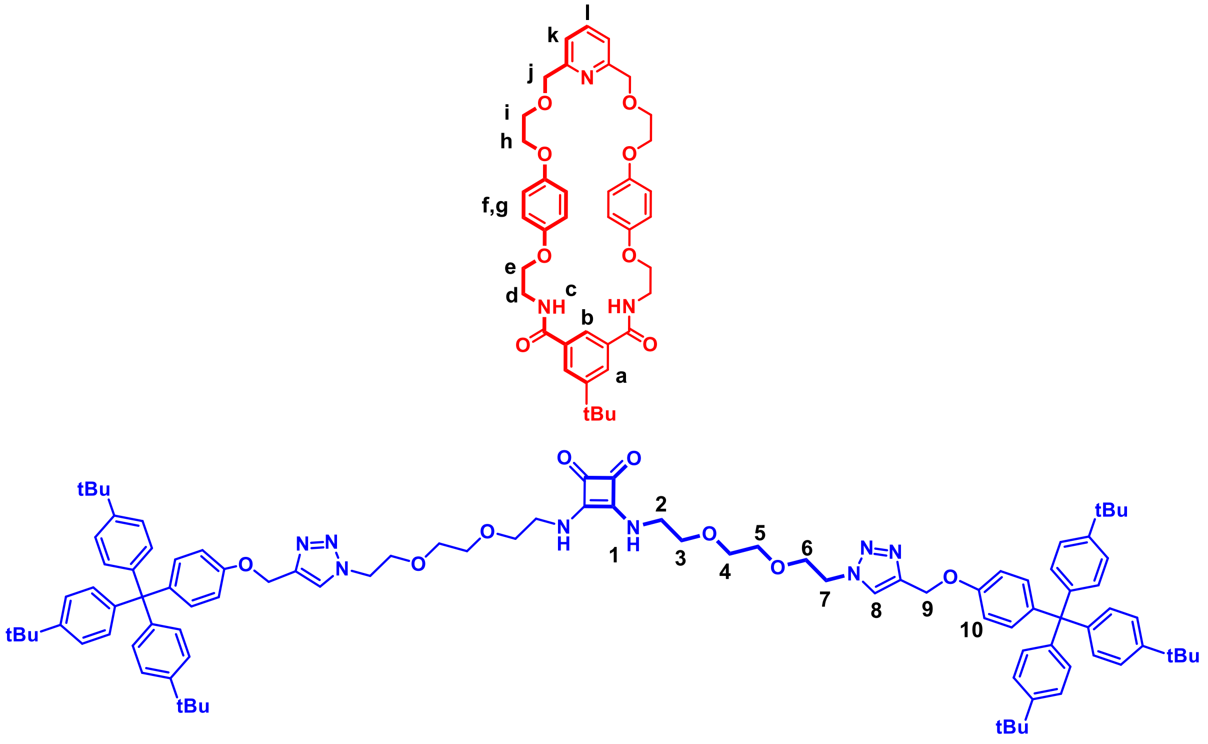

[2]rotaxane (9). Macrocycle **1** (17.00 mg, 0.025 mmol) and [Cu(CH_3CN)$_4$]PF_6 (9.27mg, 0.025 mmol) were dissolved in dry, degassed dichloromethane (1.2 mL) and stirred for 30 minutes at room temperature. A solution of bis-azide **6** (31.81 mg, 0.075 mmol) and terphenyl stopper alkyne **2** (80.97 mg, 0.15 mmol) in dichloromethane (1.2 mL) was added dropwise to the Cu-complexed macrocycle solution. The reaction mixture was stirred at room temperature for 72 hours, following which it was diluted with CH_2Cl_2 (40 mL). The organic layer was washed with EDTA/NH_4OH (2 × 25 mL) and H_2O (2 × 25 mL), dried over $MgSO_4$, filtered and concentrated under vacuum. The crude was purified by preparative TLC in 70:25:5 CH_2Cl_2/EtOAc/MeOH to afford [2]rotaxane **9** as a white solid (16.37 mg, 30%).

^{1}H NMR (500 MHz, $CDCl_3$) δ = 8.35, 8.24, 7.84, 7.67, 7.66, 7.63, 7.23, 7.21, 7.11, 7.09, 7.08, 7.05, 6.99, 6.84, 6.82, 6.71, 6.69, 6.66, 6.64, 5.10, 4.64, 4.38, 4.09, 4.02, 3.87, 3.74, 3.73, 3.69, 3.48, 3.44, 3.40, 3.38, 1.36, 1.29.

^{13}C NMR (151 MHz, $CDCl_3$) δ = 182.9, 167.8, 167.7, 157.1, 156.3, 152.9, 152.3, 152.3, 148.5, 144.2, 144.2, 140.4, 138.1, 133.9, 132.5, 131.0, 130.8, 130.6, 128.9, 124.4, 124.2, 124.0, 123.9, 121.8, 121.4, 115.5, 115.5, 113.3, 77.4, 77.2, 76.9, 73.9, 70.7, 70.4, 70.3, 70.0, 69.4, 68.3, 66.6, 63.2, 62.0, 50.3, 43.8, 39.6, 35.2, 34.4, 31.5, 31.4, 29.8, 23.3, 22.8, 14.3, 14.3, 1.2.

HRMS (ESI +ve) m/z: 2218.2129 ([M+Na]$^+$, $C_{135}H_{163}N_{11}O_{16}$ requires 2218.2205).

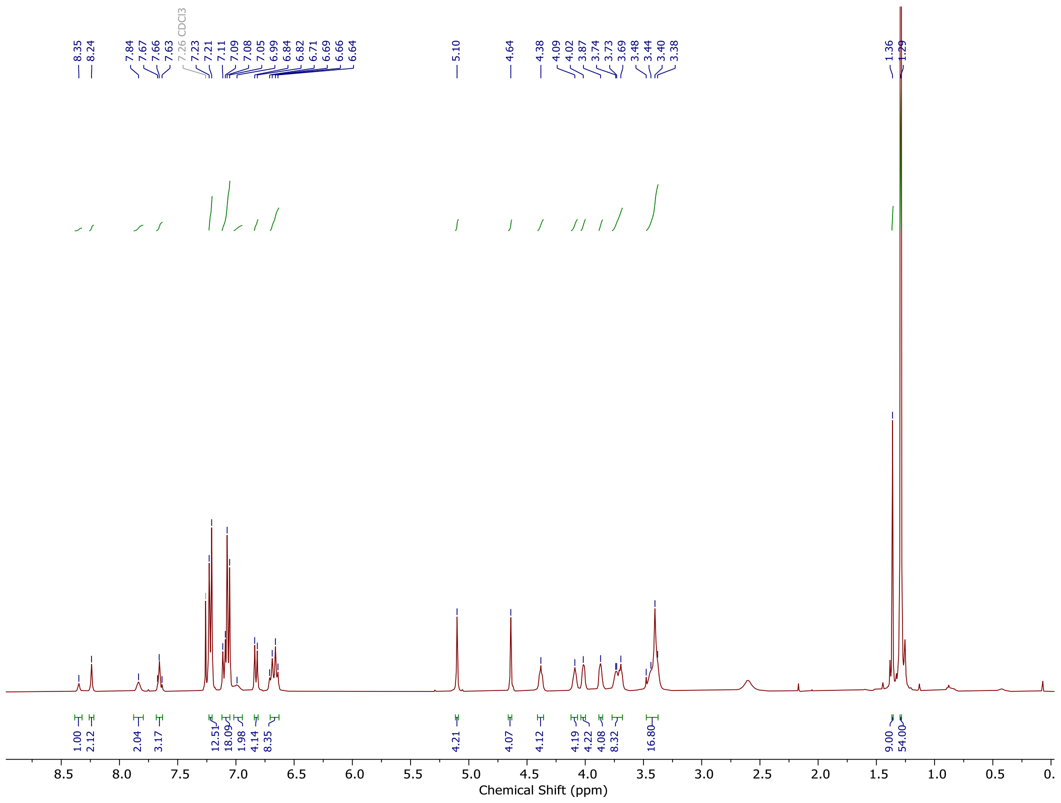

*Figure S21. ¹H-NMR spectrum of [2]rotaxane **9** (500 MHz, $CDCl_3$, 298 K)*

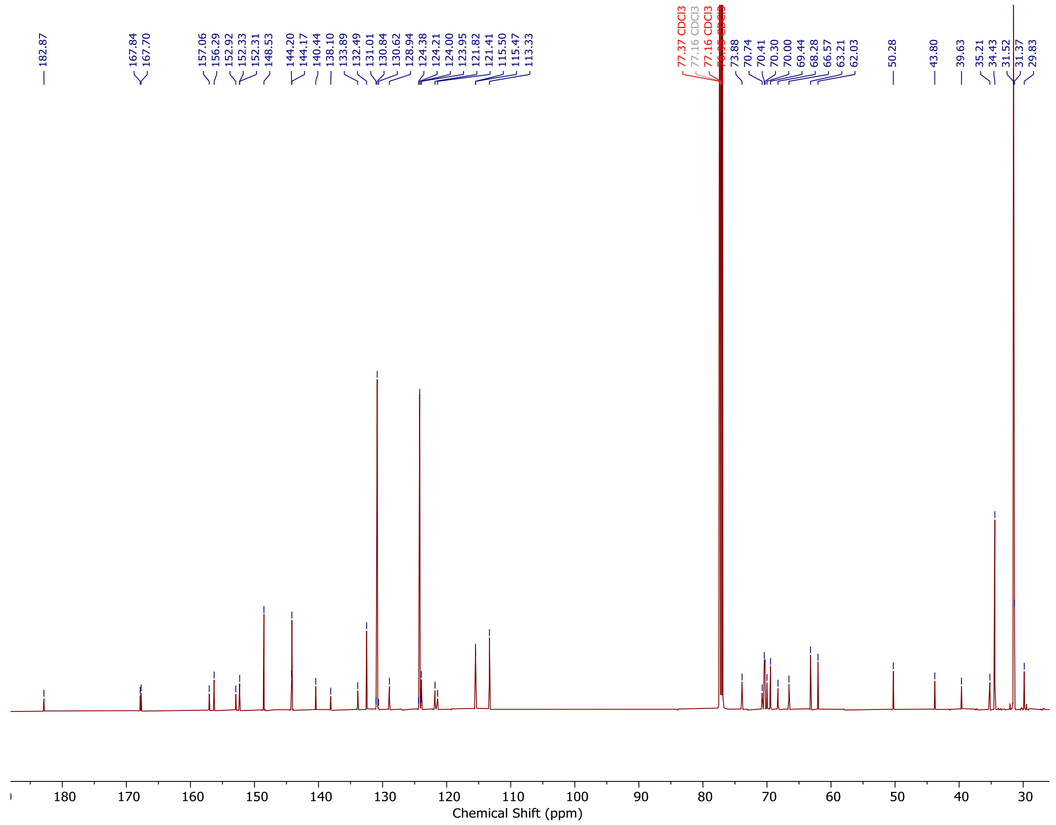

*Figure S22. ¹³C-NMR spectrum of [2]rotaxane **9** (600 MHz, $CDCl_3$, 298 K)*

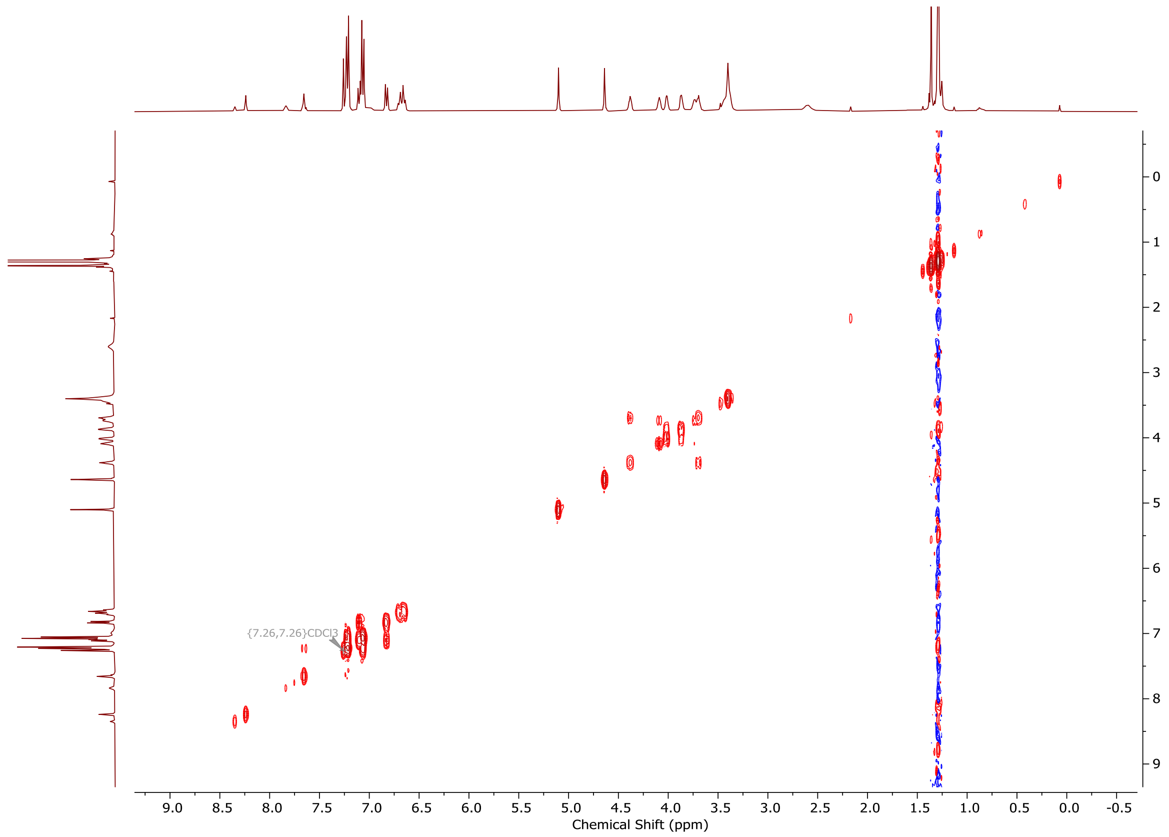

Figure S23. ^{1}H-^{1}H COSY NMR spectrum of [2]rotaxane **9** (500 MHz, $CDCl_3$, 298 K)

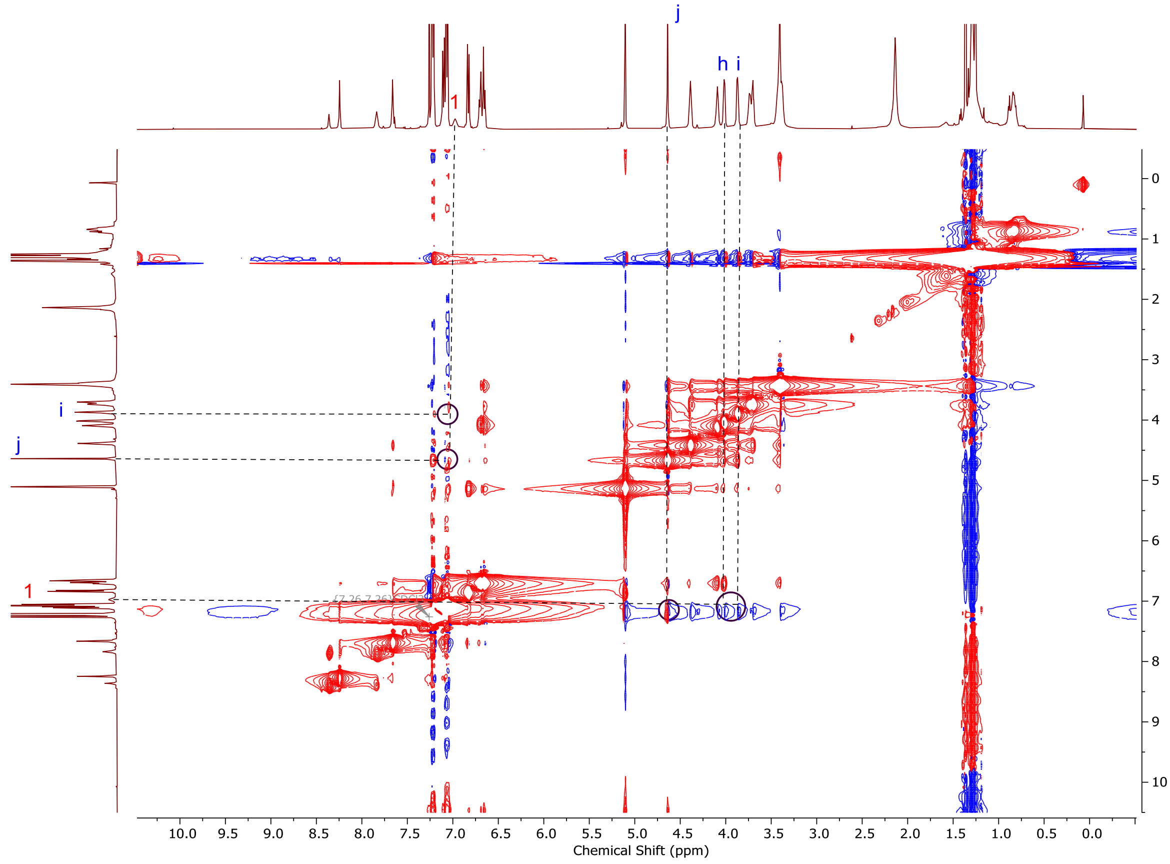

Figure S24. ^{1}H-^{1}H ROESY NMR spectrum of [2]rotaxane **9** (500 MHz, $CDCl_3$, 298 K)

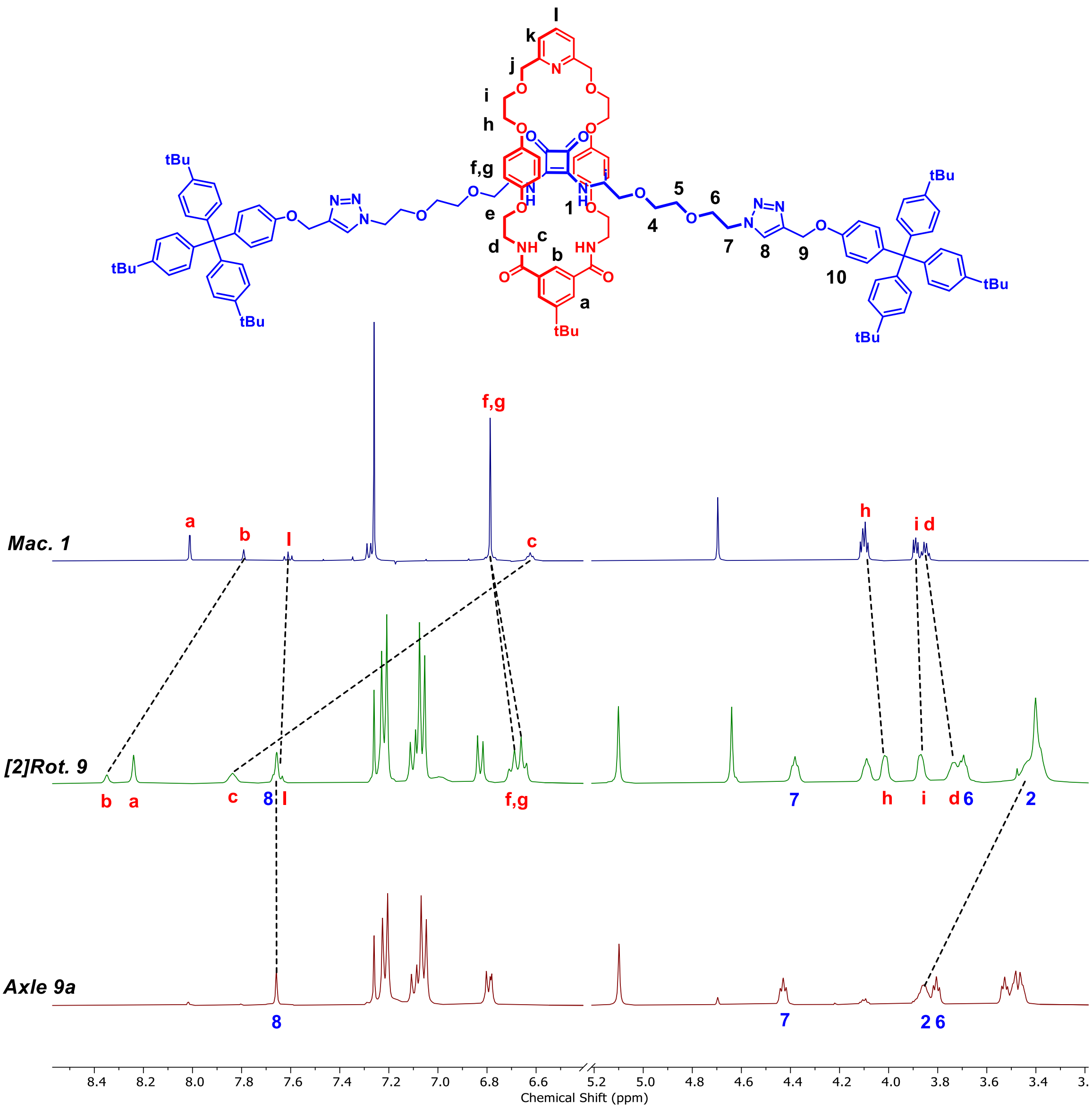

*Figure S25. Stacked ^{1}H-NMR spectra of Macrocycle **1** (top) and [2]rotaxane **9** (middle) and axle **9a** (bottom) (500 MHz, $CDCl_3$, 298 K).*

Expanded Spectrum RT 0.18, NL 73172296, Peak [1], Target Mass 2217.2172

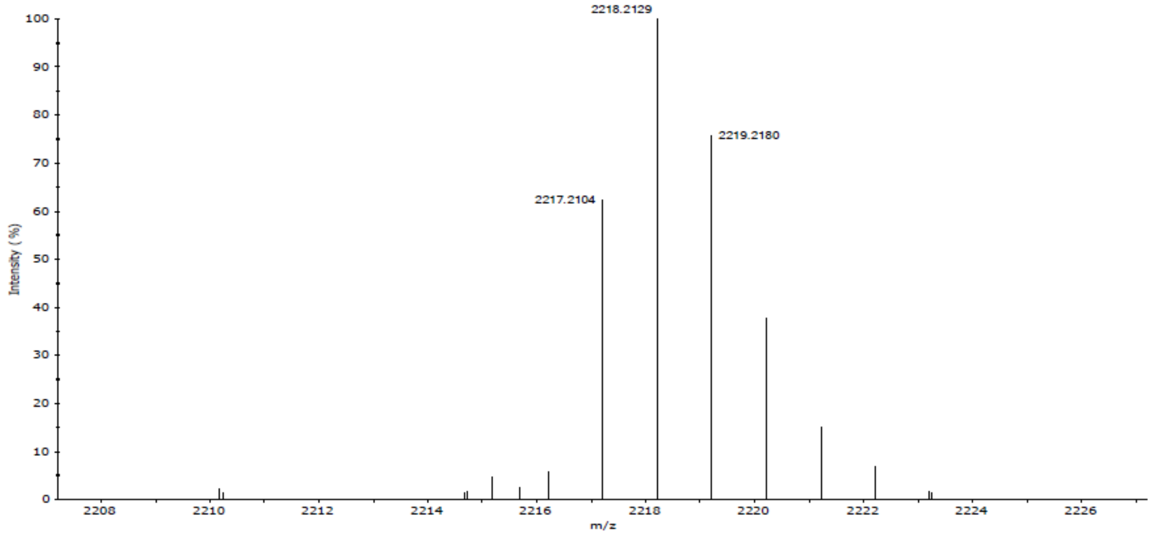

Theoretical Spectrum for C135H163N11O16Na, Minimum Abundance 0.01%

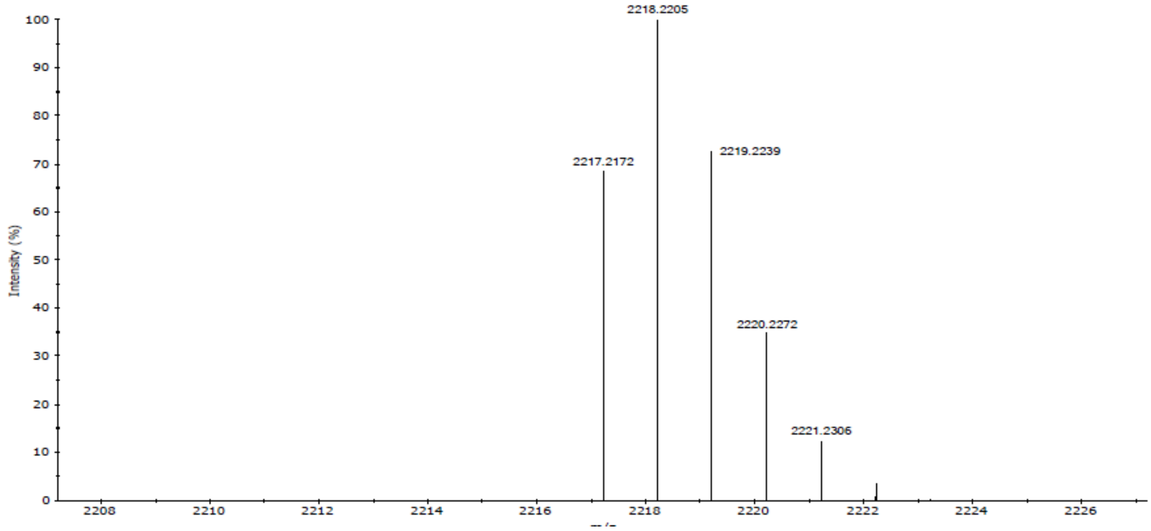

*Figure S26. High-resolution mass spectrum (ESI +ve) of **[2]rotaxane 9** (top: expanded experimental; bottom: theoretical).*

^{1}H-NMR binding studies

General procedure

All ^{1}H NMR titration experiments were performed on a Bruker AVIII 500 MHz spectrometer at 298 K. In a typical anion titration, a 1.0 mM solution of the neutral receptor was prepared in 95:5 $(CD_3)_2CO/D_2O$. A 50 mM solution of TBAX (X = Cl, Br, I, OAc, OBz, NO_3, NO_2, H_2PO_4, HSO_4) was added in aliquots to the solution containing the receptor, where 1.0 equivalent of the salt added corresponds to 10.0 µL of the salt solution. 17 spectra were recorded, corresponding to 0.0, 0.2, 0.4, 0.6, 0.8, 1.0, 1.2, 1.4, 1.6, 1.8, 2.0, 2.5, 3.0, 4.0, 5.0, 7.0, 10.0 equivalents of the added guest ion. The binding of anions to all receptors were found to be fast on the NMR timescale. For all anion titrations, the chemical shift of the internal benzene proton H_b, spacer protons H_2 were monitored and used for subsequent fitting. The values of the observed chemical shift(s) and concentration of guest at each titration point were entered into the Bindfit[41] software alongside initial estimates of the binding constants and limiting chemical shifts. These parameters were refined using nonlinear least-squares analyses to obtain the best fit between empirical and calculated chemical shifts based on five host-guest binding models (1:1, 1:2 Full, 1:2 Non-cooperative, 1:2 Additive and 1:2 Non-statistical) The input parameters were varied until convergence of the best fit values of the binding constants was attained.

^{1}H-NMR anion binding titration studies with [2]rotaxane 7

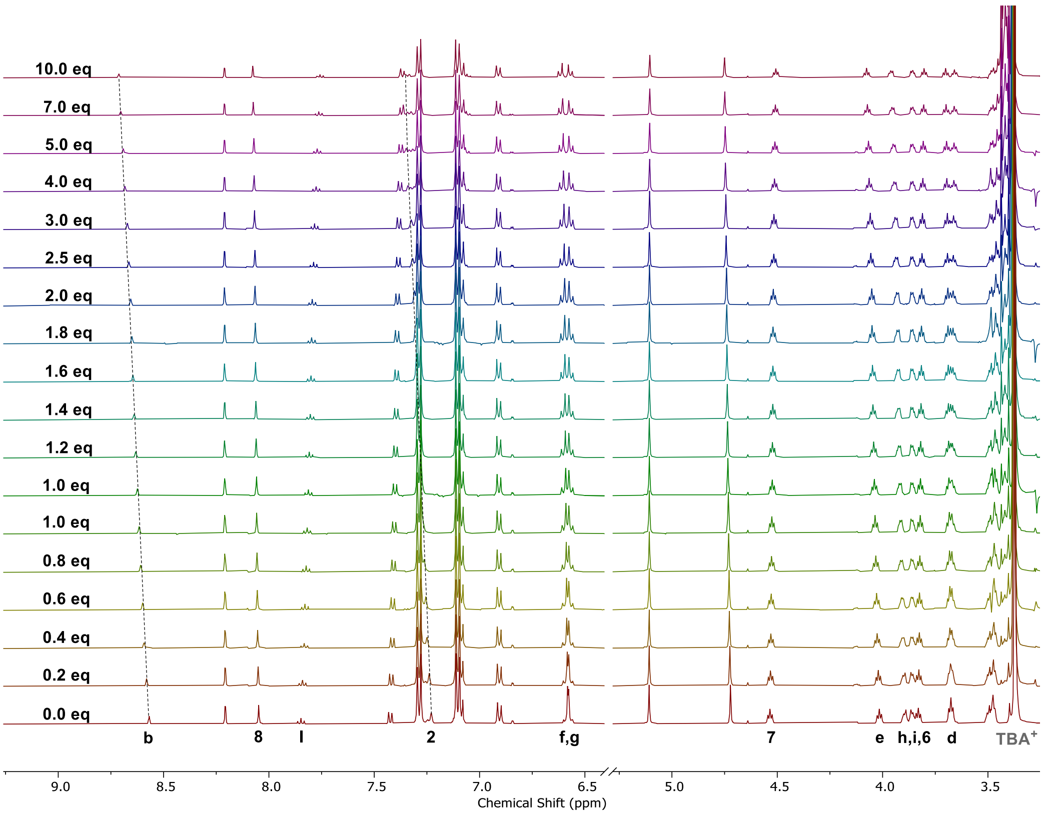

*Figure S27. Truncated ^{1}H NMR titration spectra of [2]rotaxane **7** upon progressive addition of 10 equivalents TBACl (500 MHz, 298 K, 95:5 $(CD_3)_2CO/D_2O$, [Receptor] = 1.0 mM).*

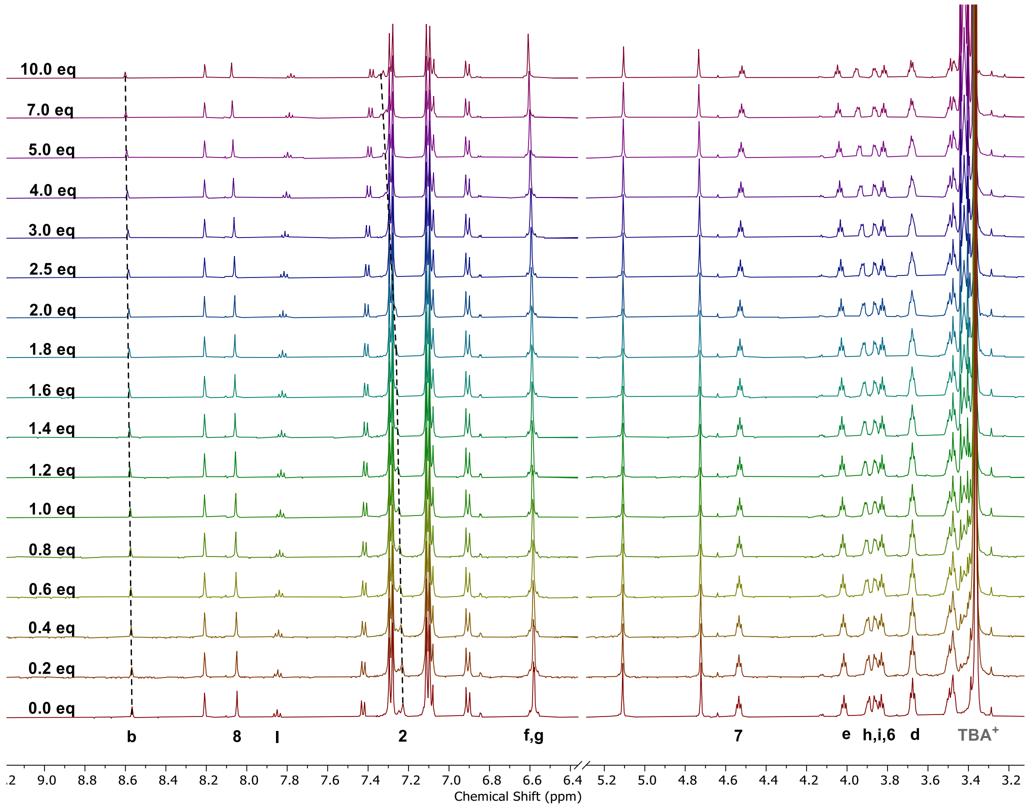

*Figure S28. Truncated ^{1}H NMR titration spectra of [2]rotaxane **7** upon progressive addition of 10 equivalents TBABr (500 MHz, 298 K, 95:5 $(CD_3)_2CO/D_2O$, [Receptor] = 1.0 mM).*

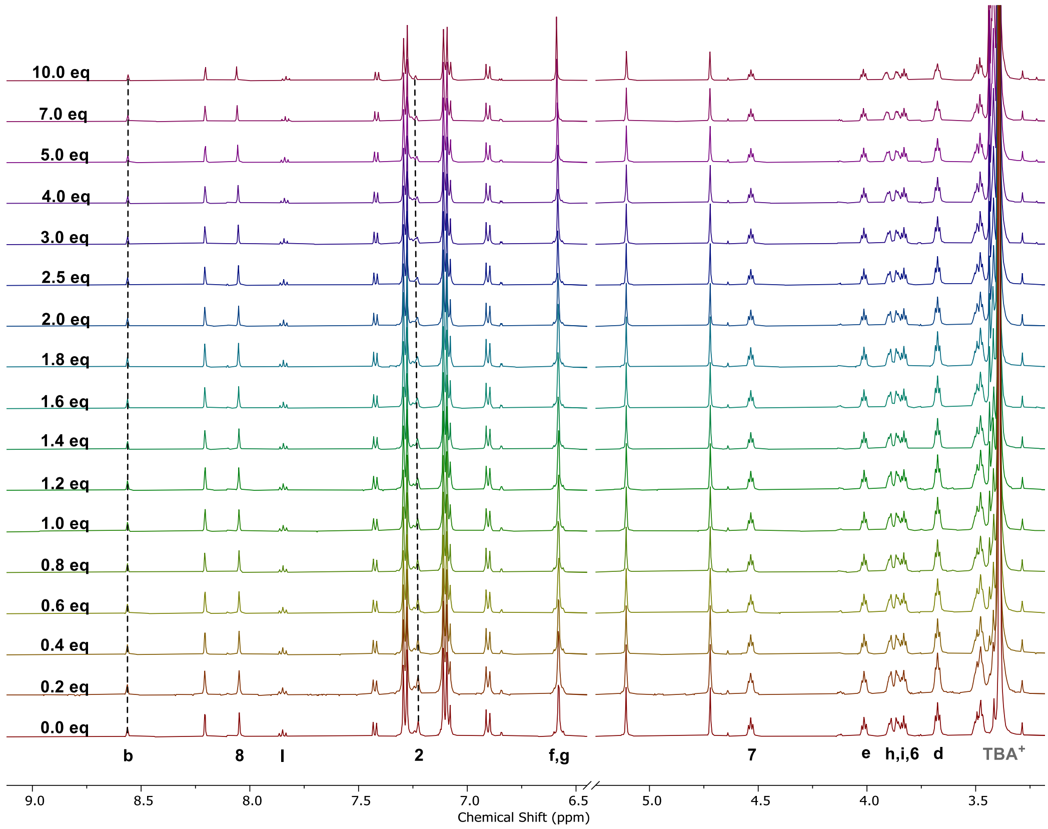

*Figure S29. Truncated ^{1}H NMR titration spectra of [2]rotaxane **7** upon progressive addition of 10 equivalents TBAI (500 MHz, 298 K, 95:5 $(CD_3)_2CO/D_2O$, [Receptor] = 1.0 mM).*

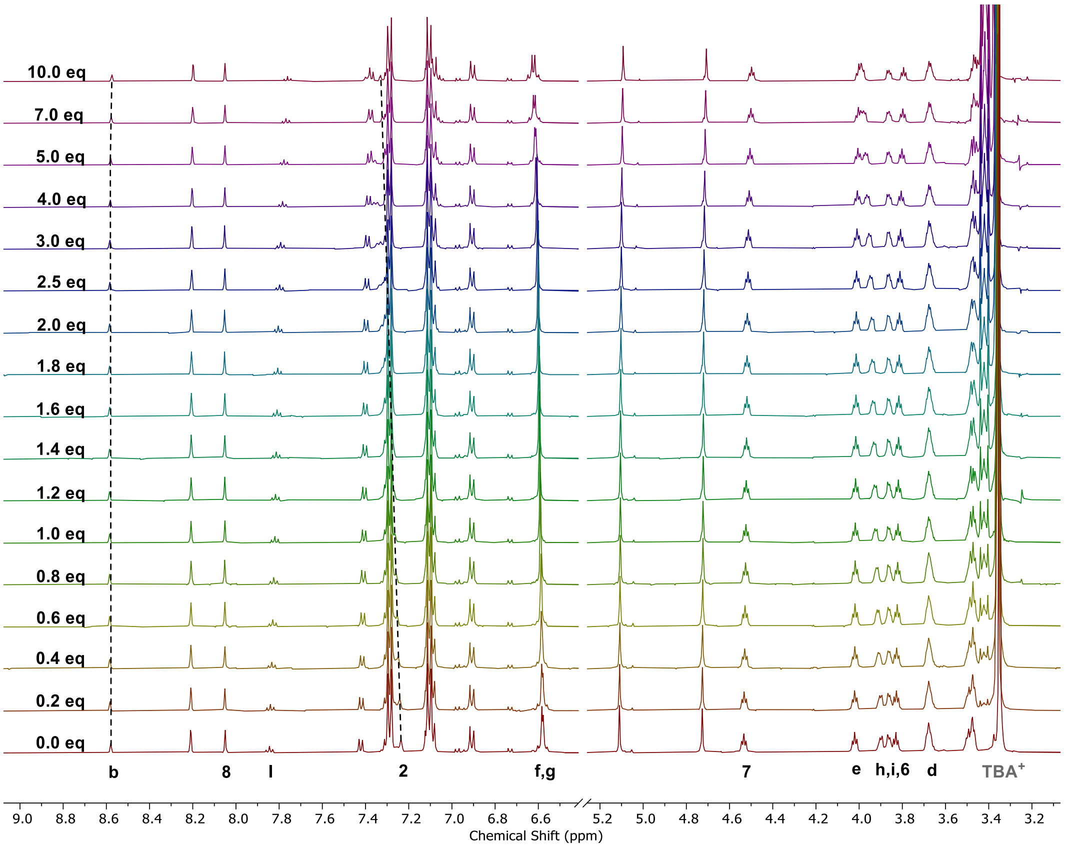

*Figure S30. Truncated ¹H NMR titration spectra of [2]rotaxane **7** upon progressive addition of 10 equivalents TBAOAc (500 MHz, 298 K, 95:5 $(CD_3)_2CO/D_2O$, [Receptor] = 1.0 mM).*

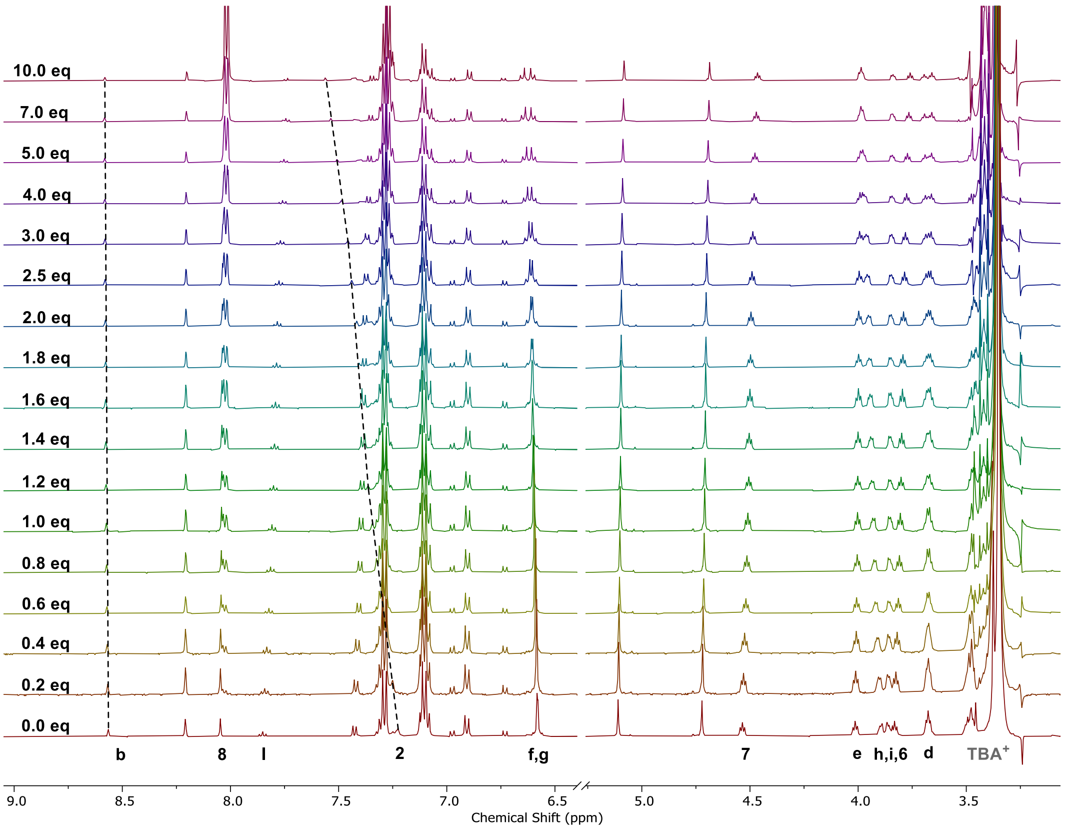

*Figure S31. Truncated ^{1}H NMR titration spectra of [2]rotaxane **7** upon progressive addition of 10 equivalents TBAOBz (500 MHz, 298 K, 95:5 $(CD_3)_2CO/D_2O$, [Receptor] = 1.0 mM).*

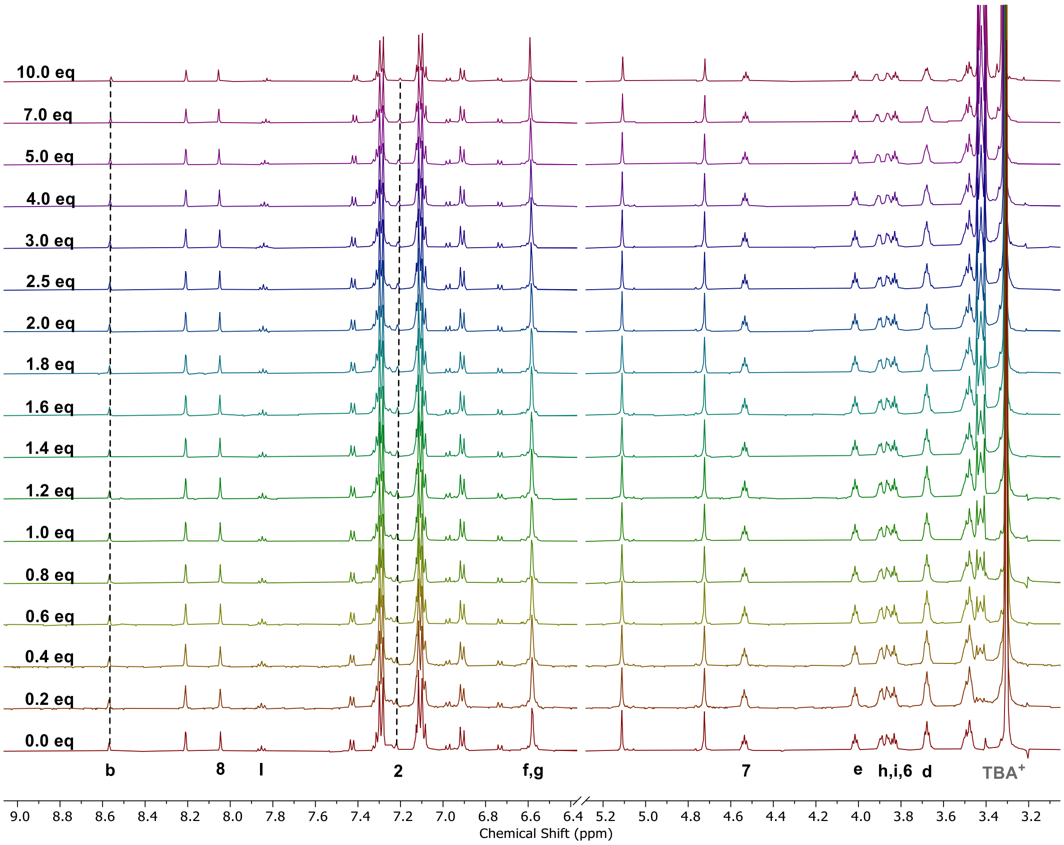

*Figure S32. Truncated ^{1}H NMR titration spectra of [2]rotaxane **7** upon progressive addition of 10 equivalents $TBANO_3$ (500 MHz, 298 K, 95:5 $(CD_3)_2CO/D_2O$, [Receptor] = 1.0 mM).*

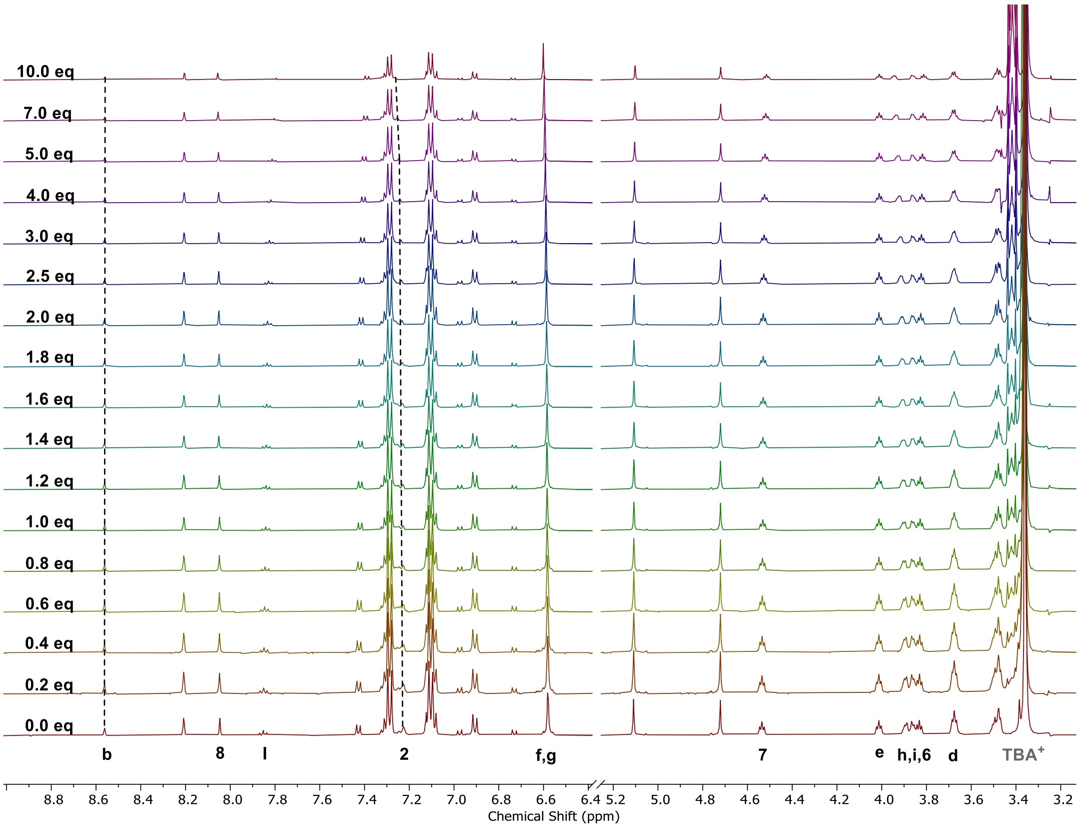

*Figure S33. Truncated ¹H NMR titration spectra of [2]rotaxane **7** upon progressive addition of 10 equivalents TBANO$_2$ (500 MHz, 298 K, 95:5 (CD$_3$)$_2$CO/D$_2$O, [Receptor] = 1.0 mM).*

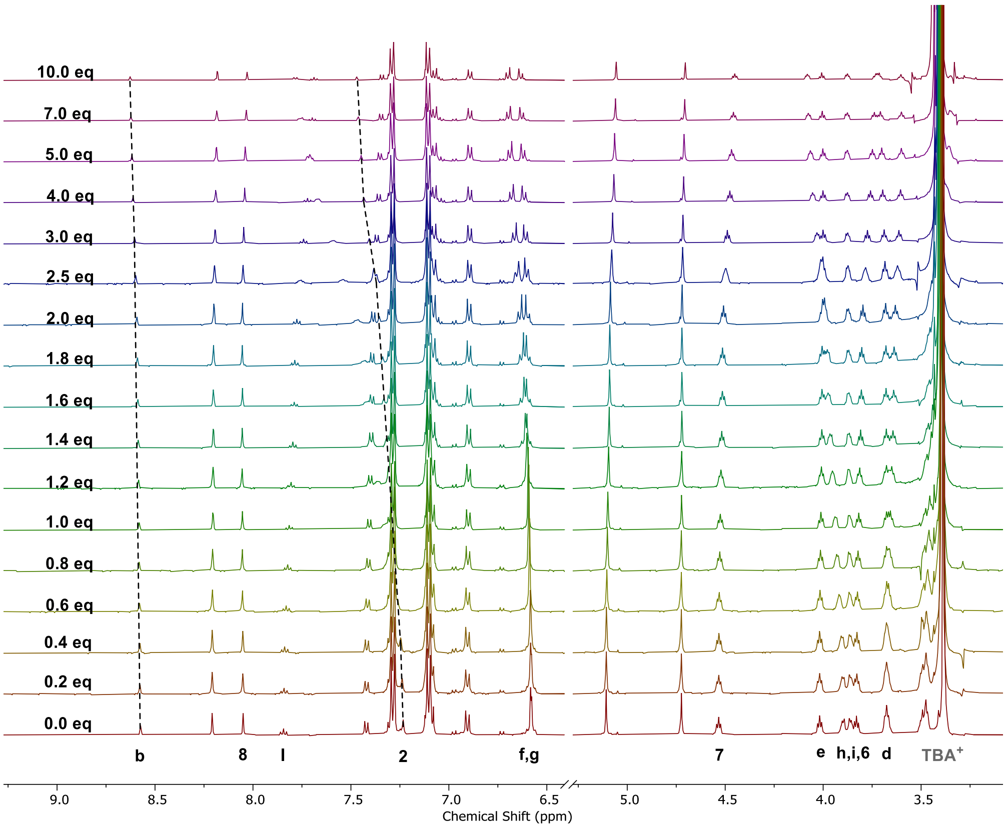

*Figure S34. Truncated ¹H NMR titration spectra of [2]rotaxane **7** upon progressive addition of 10 equivalents TBAH$_2$PO$_4$ (500 MHz, 298 K, 95:5 (CD$_3$)$_2$CO/D$_2$O, [Receptor] = 1.0 mM).*

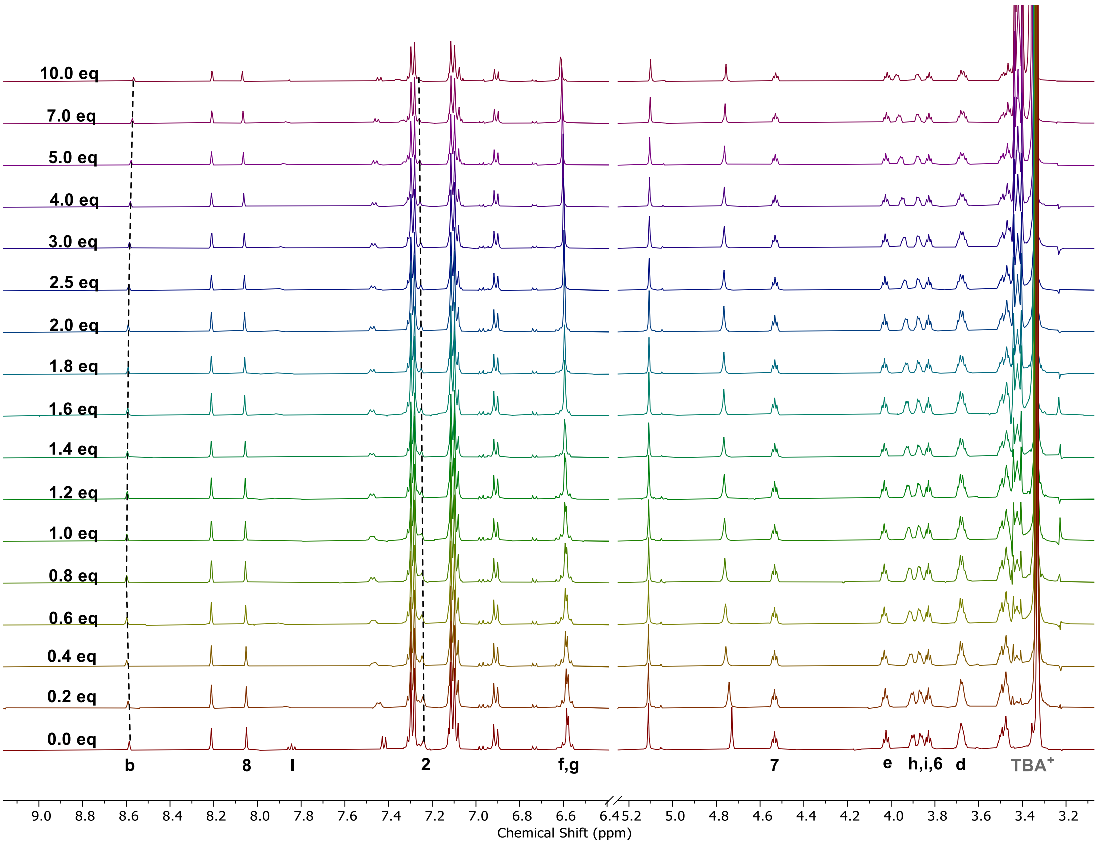

*Figure S35. Truncated ^{1}H NMR titration spectra of [2]rotaxane **7** upon progressive addition of 10 equivalents TBAHSO$_4$ (500 MHz, 298 K, 95:5 $(CD_3)_2CO/D_2O$, [Receptor] = 1.0 mM).*

^{1}H-NMR anion binding titration studies with [2]rotaxane 8

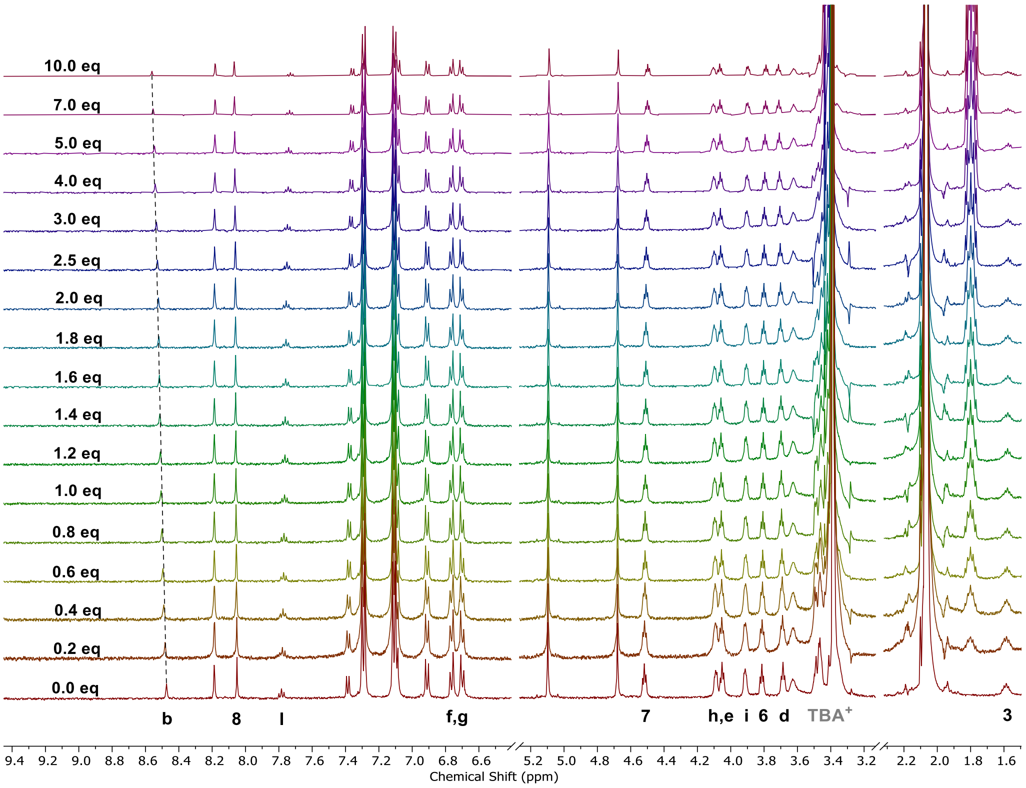

*Figure S36. Truncated ^{1}H NMR titration spectra of [2]rotaxane **8** upon progressive addition of 10 equivalents TBACl (500 MHz, 298 K, 95:5 $(CD_3)_2CO/D_2O$, [Receptor] = 1.0 mM).*

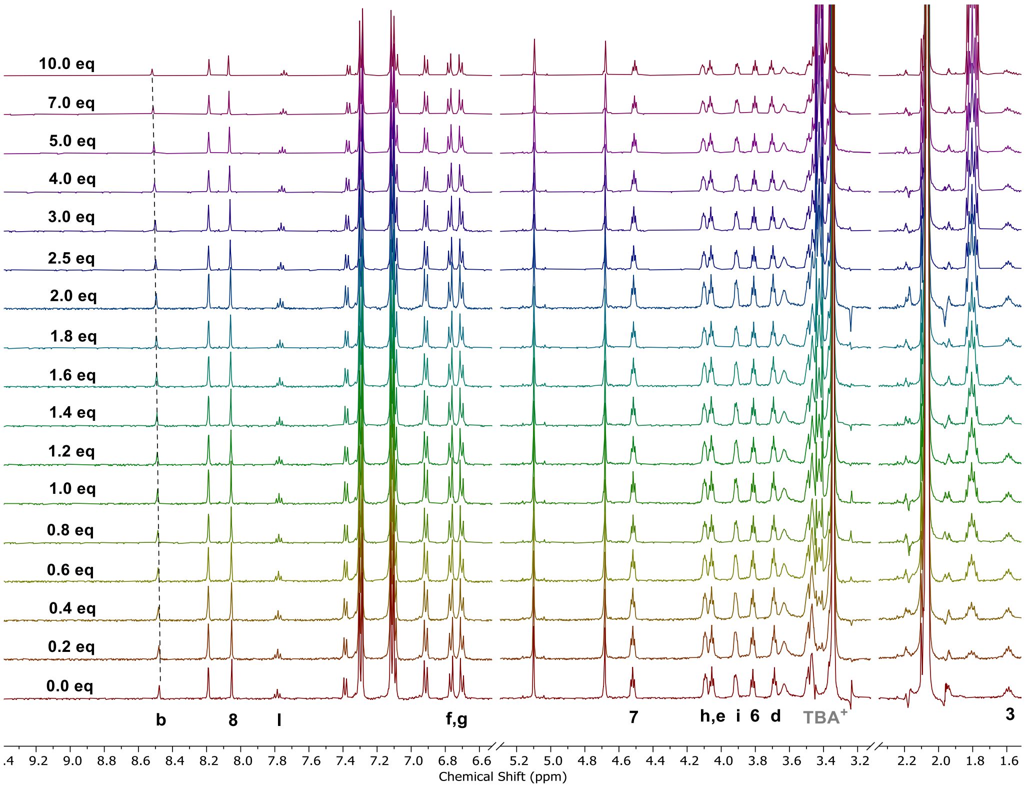

*Figure S37. Truncated ^{1}H NMR titration spectra of [2]rotaxane **8** upon progressive addition of 10 equivalents TBABr (500 MHz, 298 K, 95:5 $(CD_3)_2CO/D_2O$, [Receptor] = 1.0 mM).*

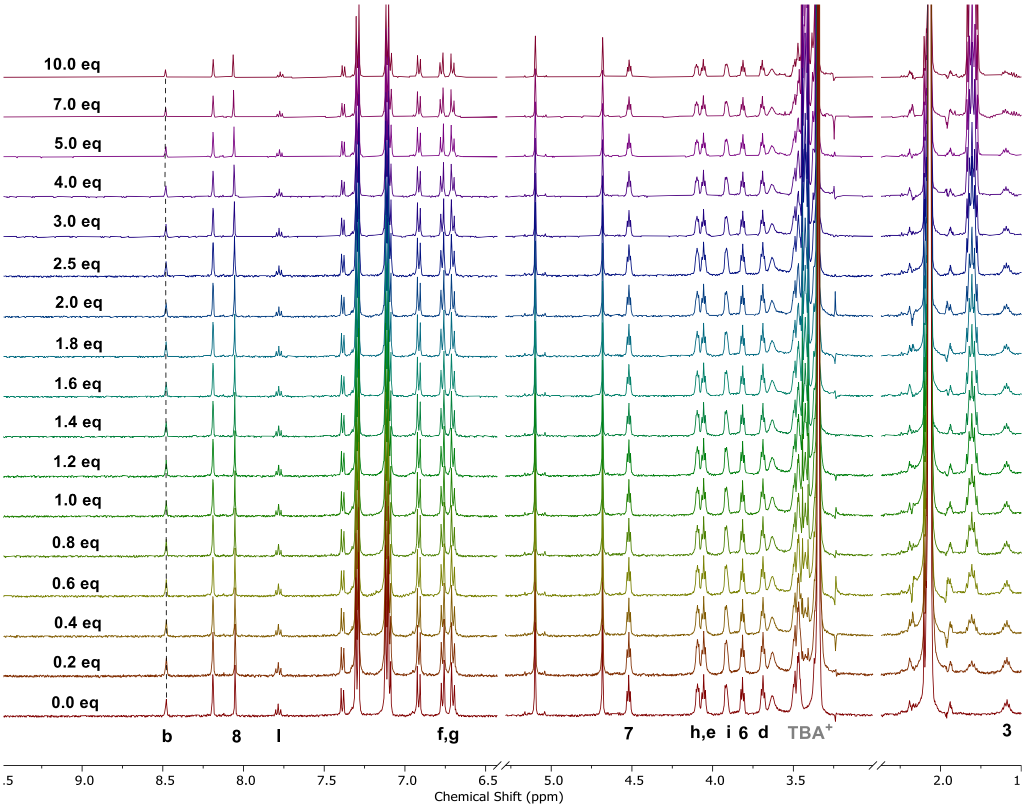

*Figure S38. Truncated ^{1}H NMR titration spectra of [2]rotaxane **8** upon progressive addition of 10 equivalents TBAI (500 MHz, 298 K, 95:5 $(CD_3)_2CO/D_2O$, [Receptor] = 1.0 mM).*

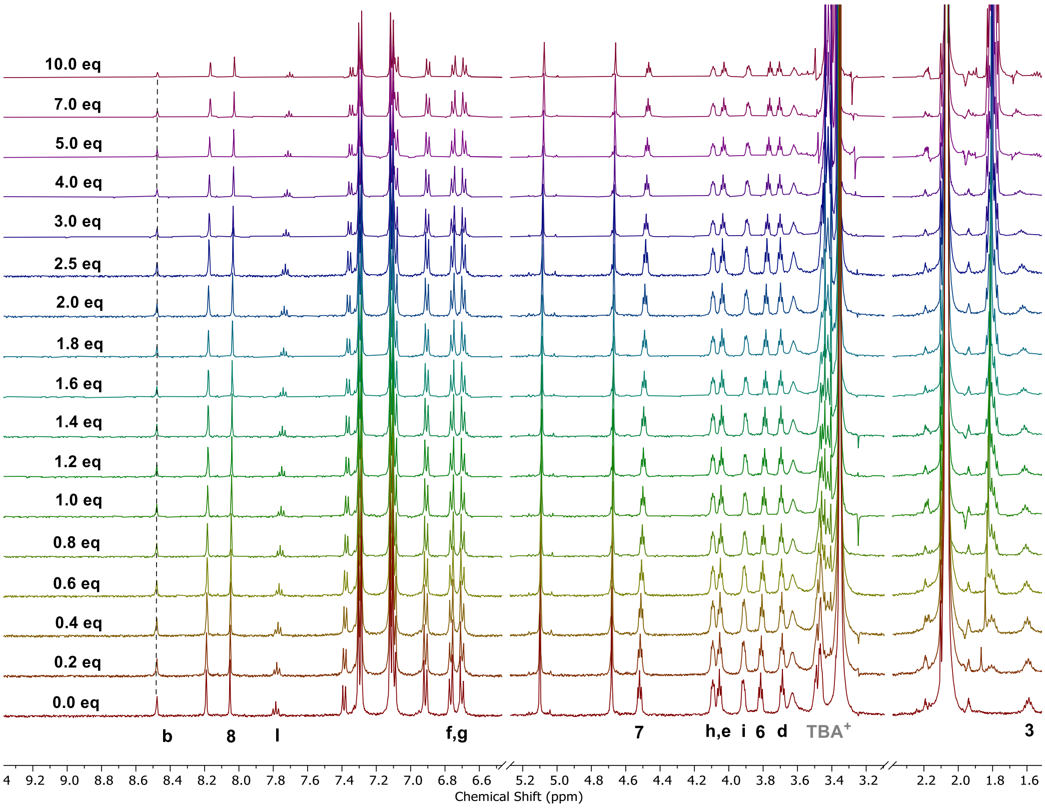

*Figure S39. Truncated ^{1}H NMR titration spectra of [2]rotaxane **8** upon progressive addition of 10 equivalents TBAOAc (500 MHz, 298 K, 95:5 $(CD_3)_2CO/D_2O$, [Receptor] = 1.0 mM).*

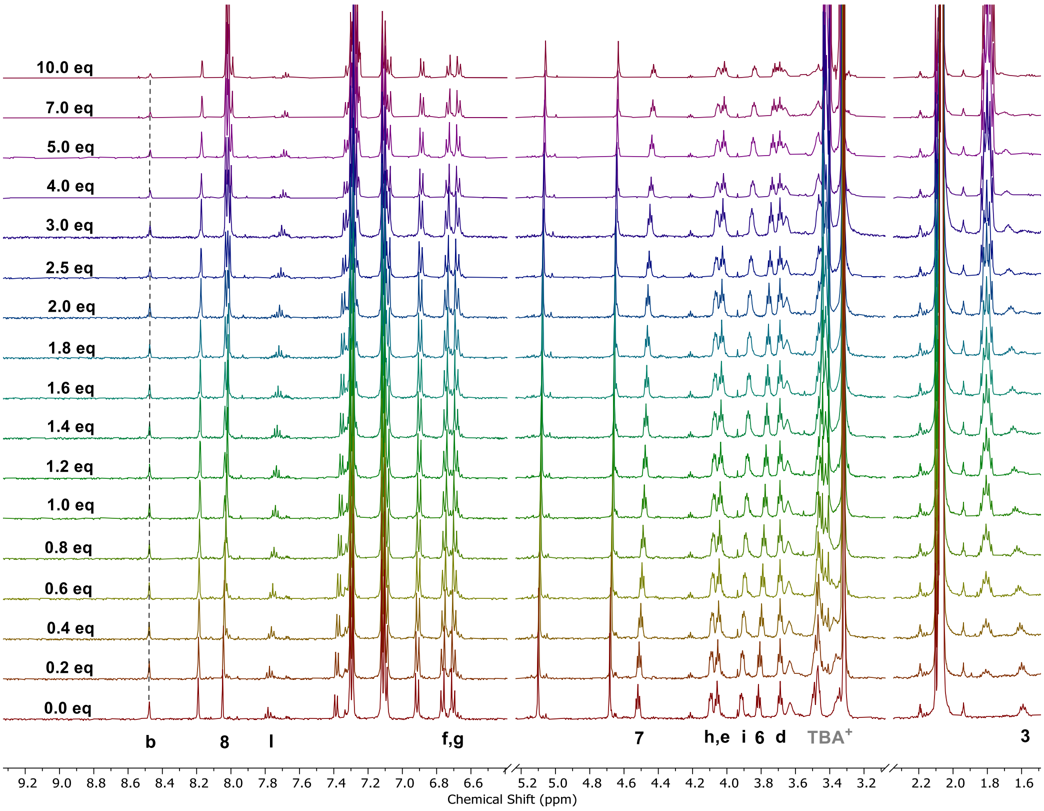

*Figure S40. Truncated ^{1}H NMR titration spectra of [2]rotaxane **8** upon progressive addition of 10 equivalents TBAOBz (500 MHz, 298 K, 95:5 $(CD_3)_2CO/D_2O$, [Receptor] = 1.0 mM).*

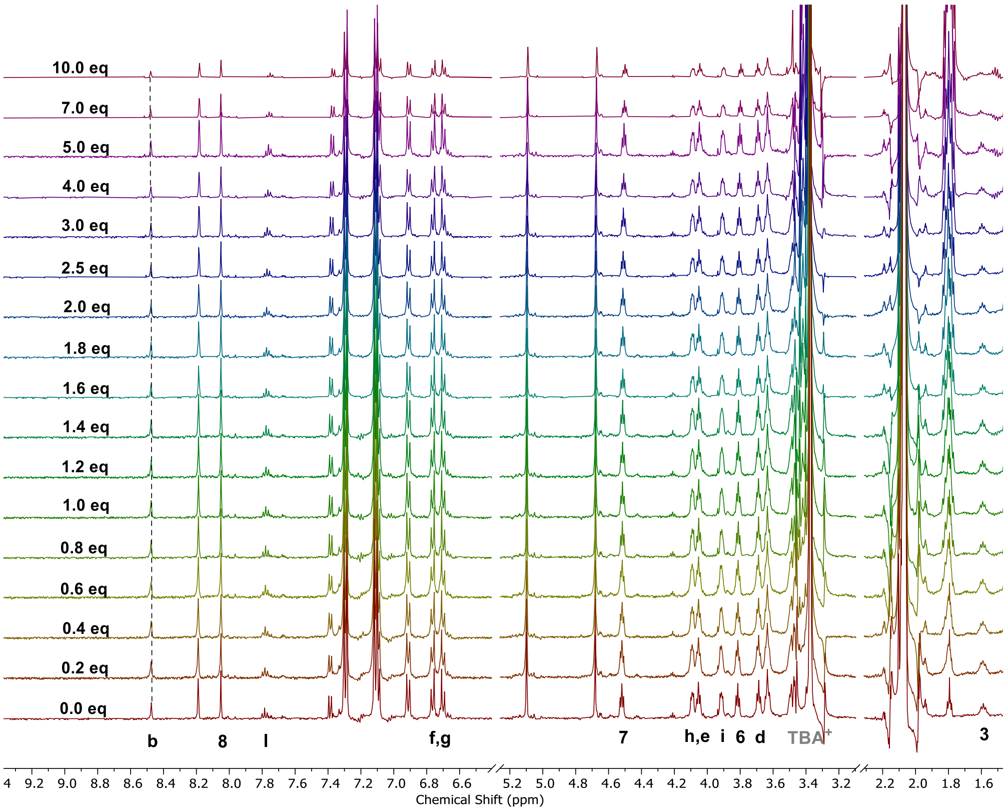

*Figure S41. Truncated ^{1}H NMR titration spectra of [2]rotaxane **8** upon progressive addition of 10 equivalents $TBANO_3$ (500 MHz, 298 K, 95:5 $(CD_3)_2CO/D_2O$, [Receptor] = 1.0 mM).*

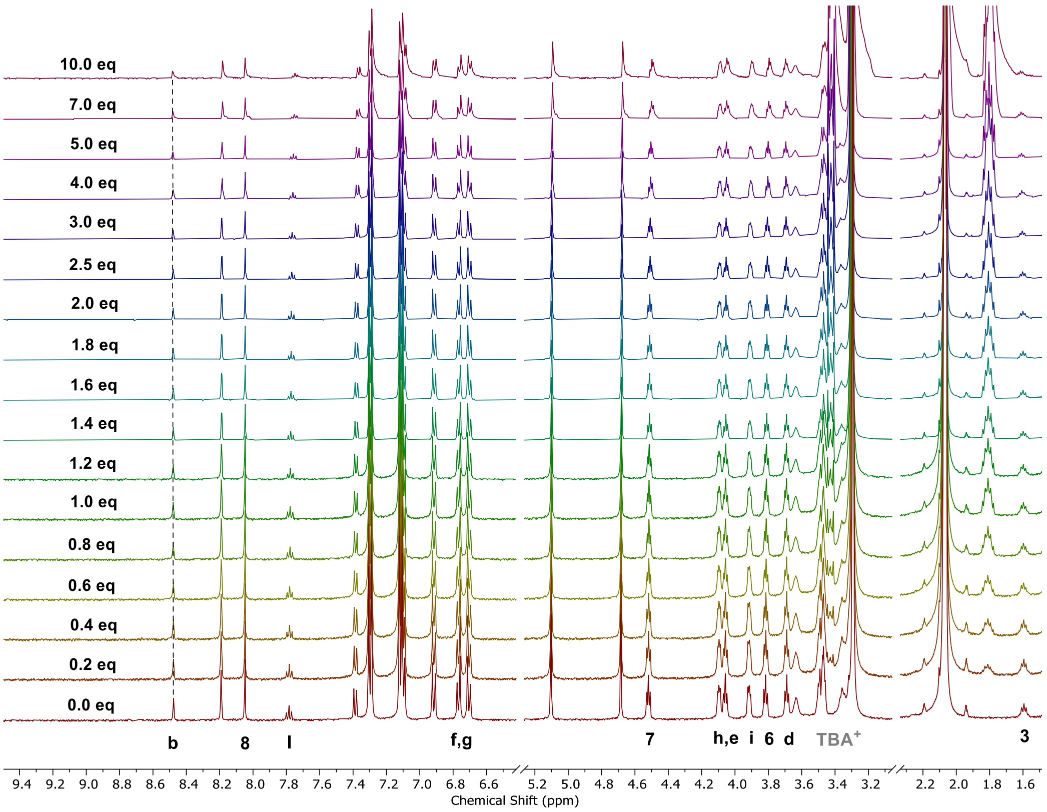

*Figure S42. Truncated ^{1}H NMR titration spectra of [2]rotaxane **8** upon progressive addition of 10 equivalents TBANO$_2$ (500 MHz, 298 K, 95:5 (CD$_3$)$_2$CO/D$_2$O, [Receptor] = 1.0 mM).*

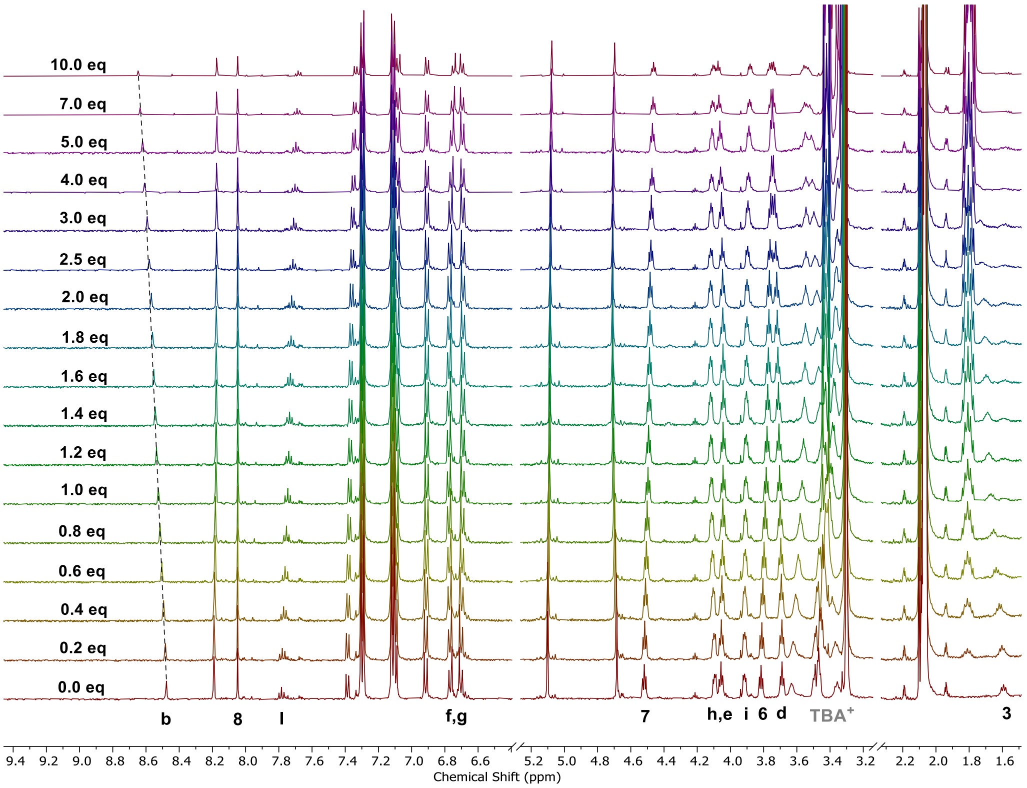

*Figure S43. Truncated ^{1}H NMR titration spectra of [2]rotaxane **8** upon progressive addition of 10 equivalents TBAH$_2$PO$_4$ (500 MHz, 298 K, 95:5 (CD$_3$)$_2$CO/D$_2$O, [Receptor] = 1.0 mM).*

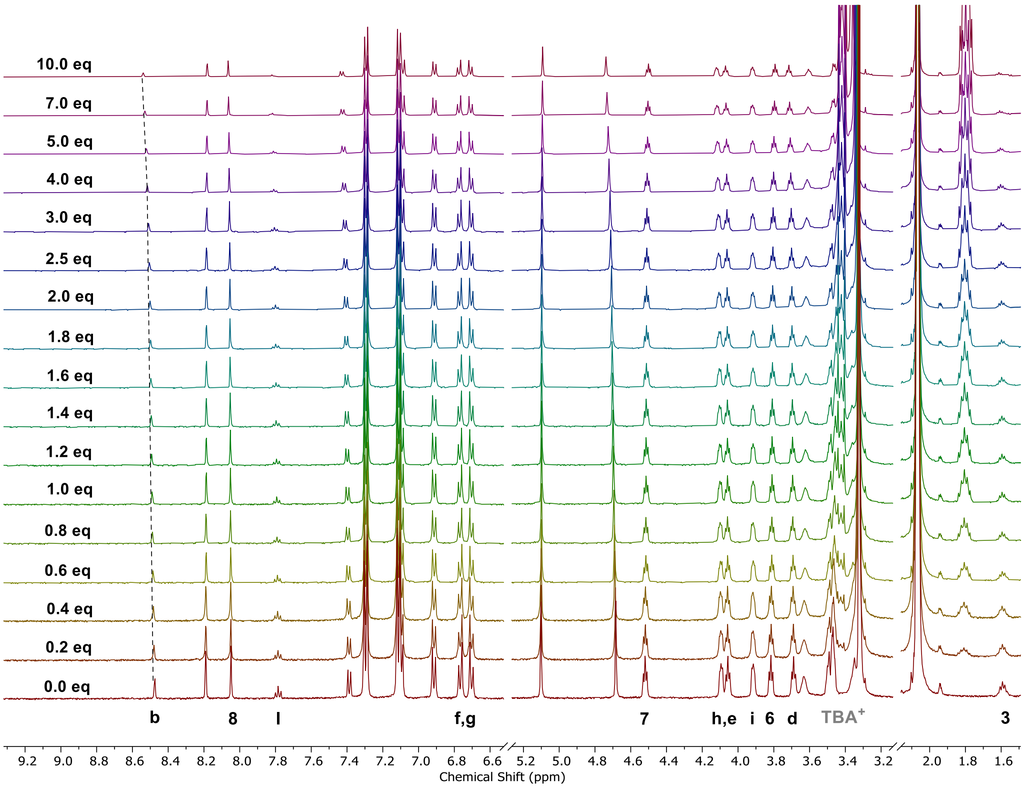

*Figure S44. Truncated ^{1}H NMR titration spectra of [2]rotaxane **8** upon progressive addition of 10 equivalents TBAHSO$_4$ (500 MHz, 298 K, 95:5 $(CD_3)_2CO/D_2O$, [Receptor] = 1.0 mM).*

^{1}H-NMR anion binding titration studies with [2]rotaxane 9

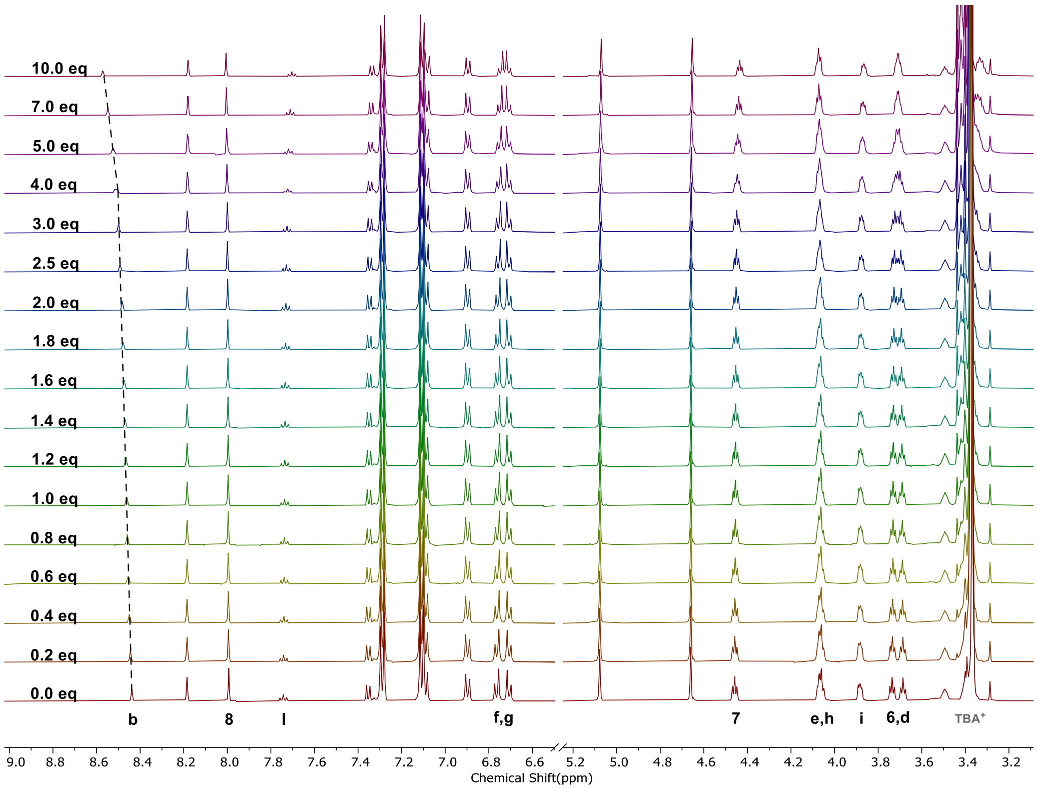

*Figure S45. Truncated ^{1}H NMR titration spectra of [2]rotaxane **9** upon progressive addition of 10 equivalents TBACl (500 MHz, 298 K, 95:5 $(CD_3)_2CO/D_2O$, [Receptor] = 1.0 mM).*

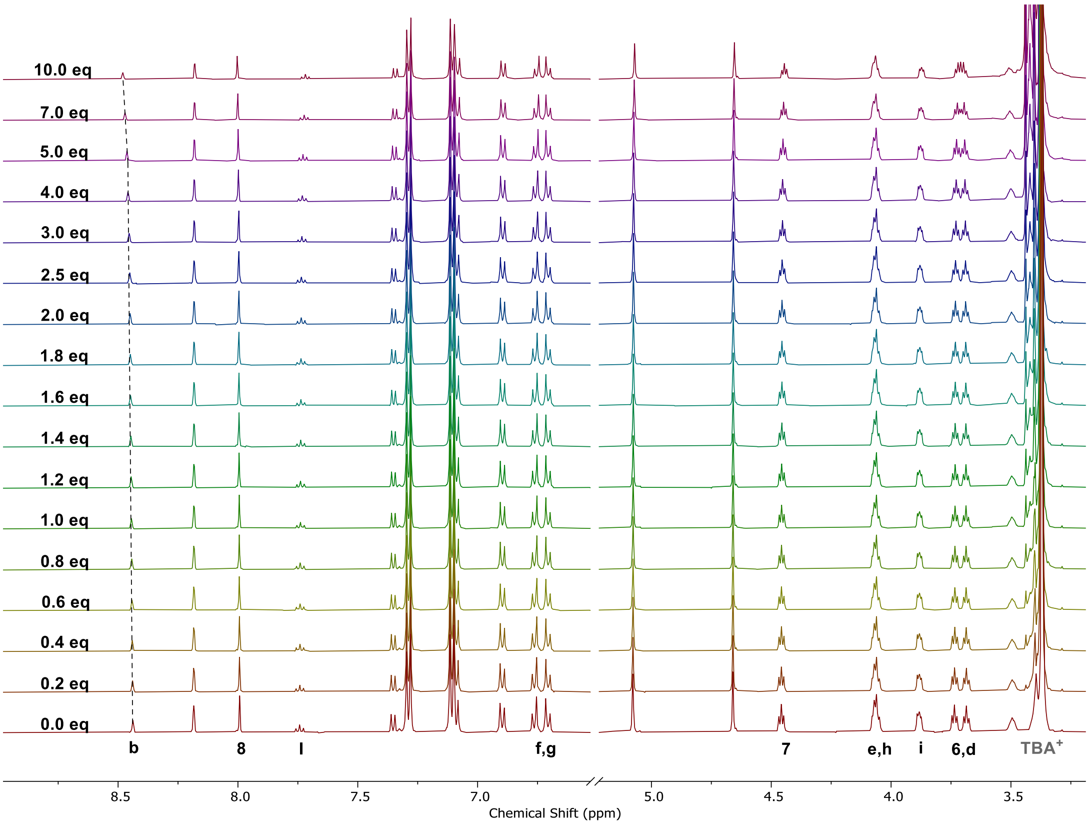

*Figure S46. Truncated ^{1}H NMR titration spectra of [2]rotaxane **9** upon progressive addition of 10 equivalents TBABr (500 MHz, 298 K, 95:5 $(CD_3)_2CO/D_2O$, [Receptor] = 1.0 mM).*

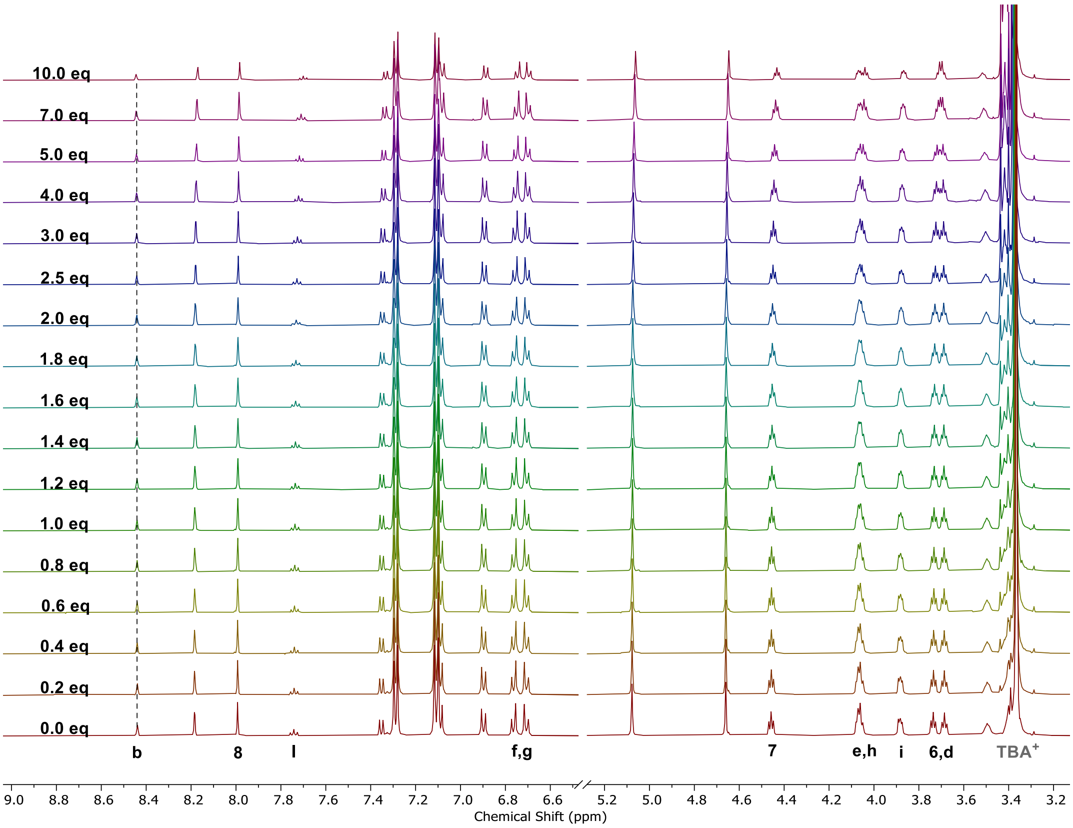

*Figure S47. Truncated ^{1}H NMR titration spectra of [2]rotaxane **9** upon progressive addition of 10 equivalents TBAOAc (500 MHz, 298 K, 95:5 $(CD_3)_2CO/D_2O$, [Receptor] = 1.0 mM).*

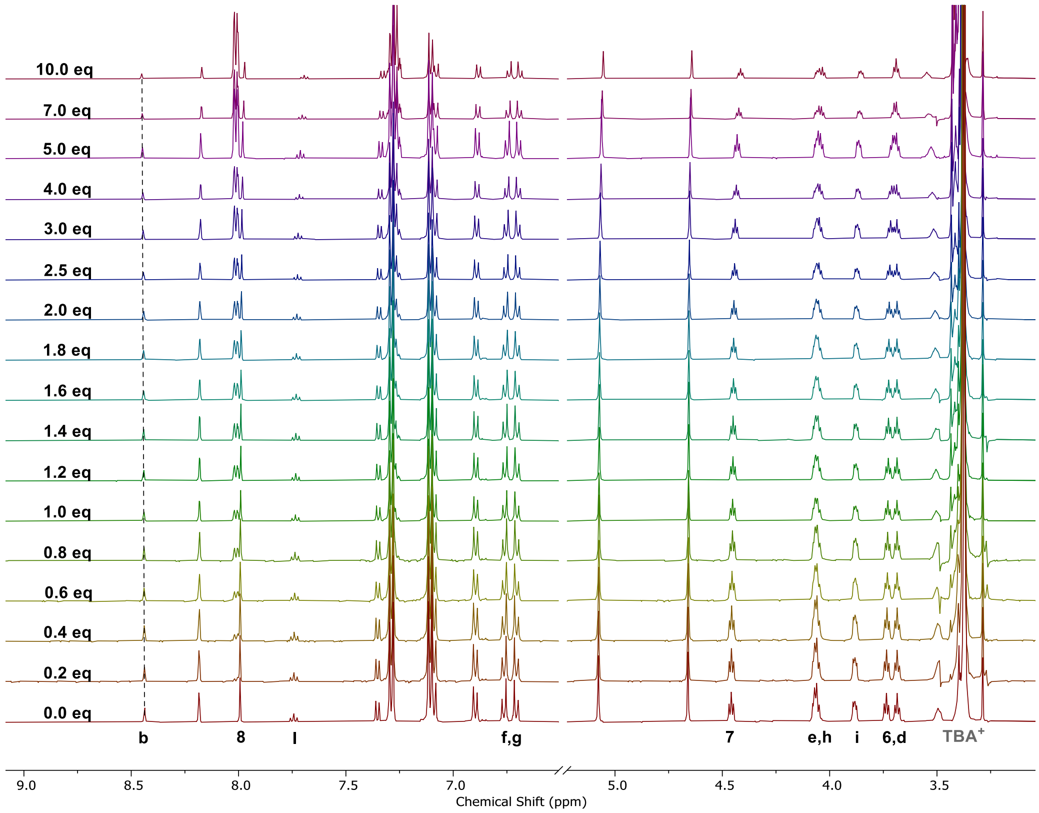

*Figure S48. Truncated ^{1}H NMR titration spectra of [2]rotaxane **9** upon progressive addition of 10 equivalents TBAOBz (500 MHz, 298 K, 95:5 $(CD_3)_2CO/D_2O$, [Receptor] = 1.0 mM).*

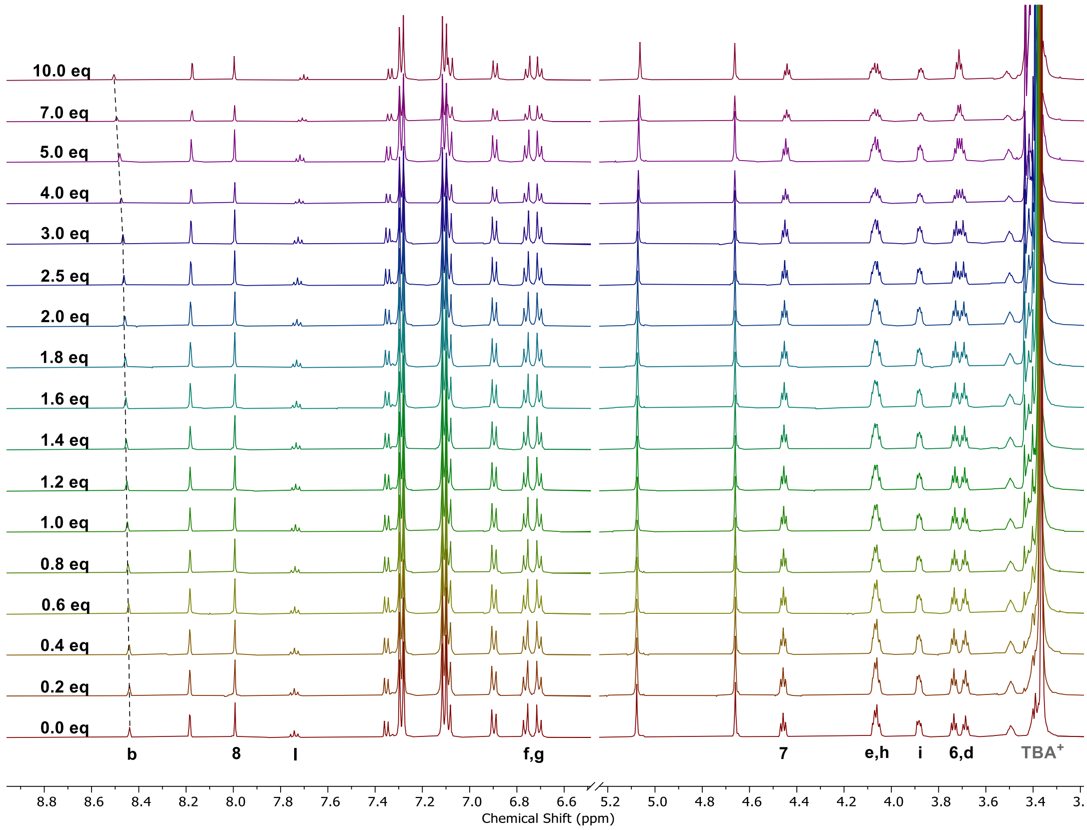

*Figure S49. Truncated ^{1}H NMR titration spectra of [2]rotaxane **9** upon progressive addition of 10 equivalents $TBAH_2PO_4$ (500 MHz, 298 K, 95:5 $(CD_3)_2CO/D_2O$, [Receptor] = 1.0 mM).*

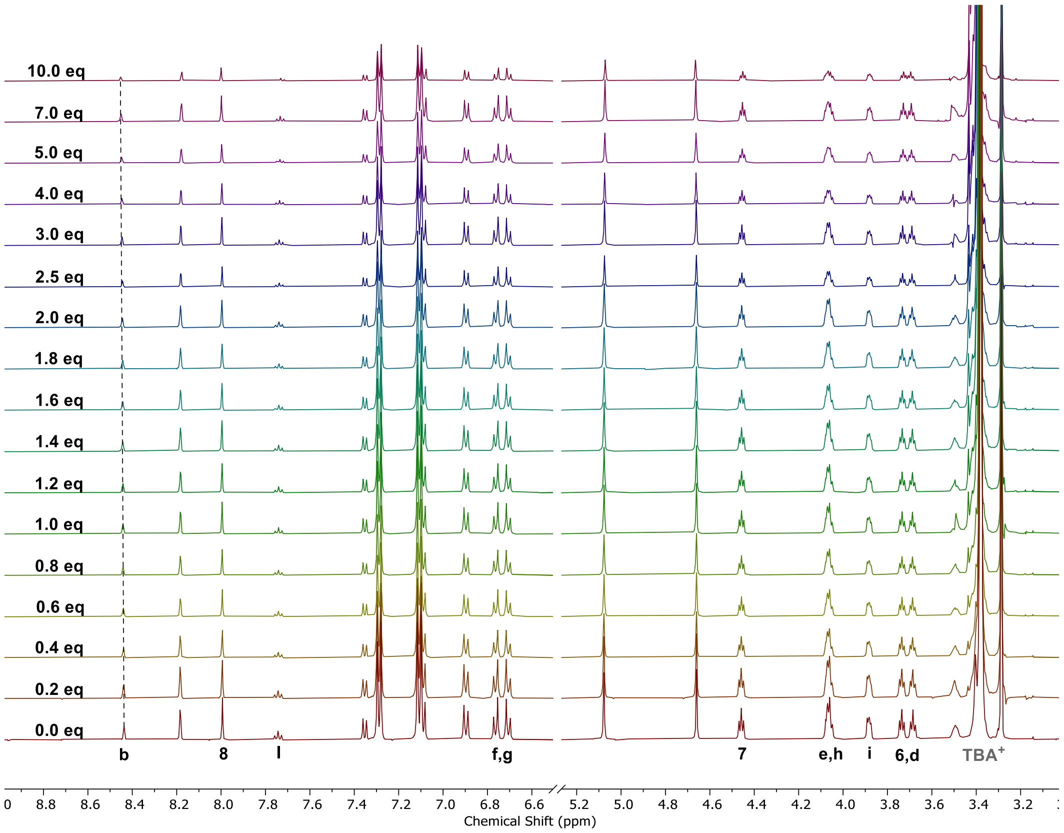

*Figure S50. Truncated ^{1}H NMR titration spectra of [2]rotaxane **9** upon progressive addition of 10 equivalents TBAHSO$_4$ (500 MHz, 298 K, 95:5 $(CD_3)_2CO/D_2O$, [Receptor] = 1.0 mM).*

Anion binding isotherms

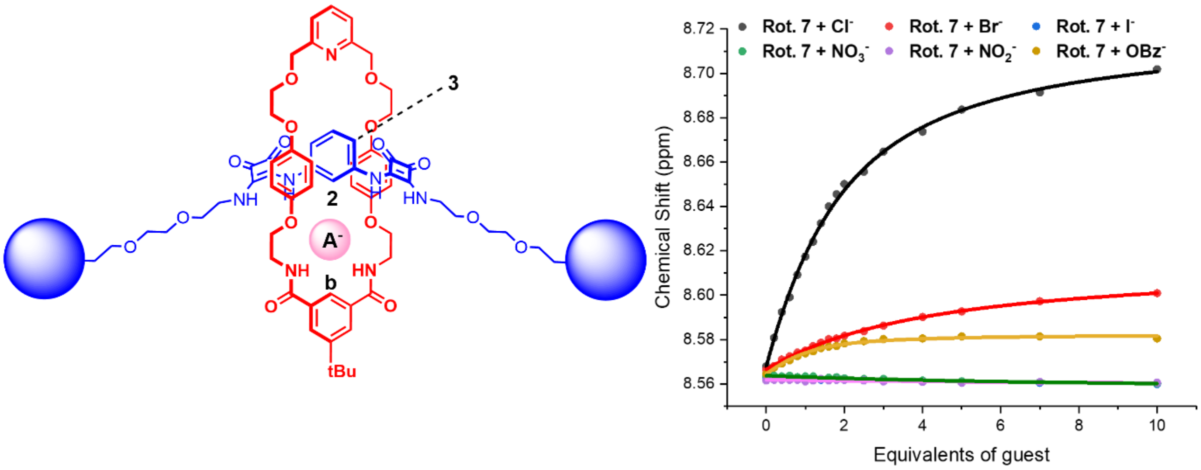

*Figure S51. Binding isotherms of [2]rotaxane **7** showing changes in chemical shift of internal benzene proton b upon increasing equivalents of various anions. (500 MHz, 298 K, 95:5-d6-acetone-D_2O).*

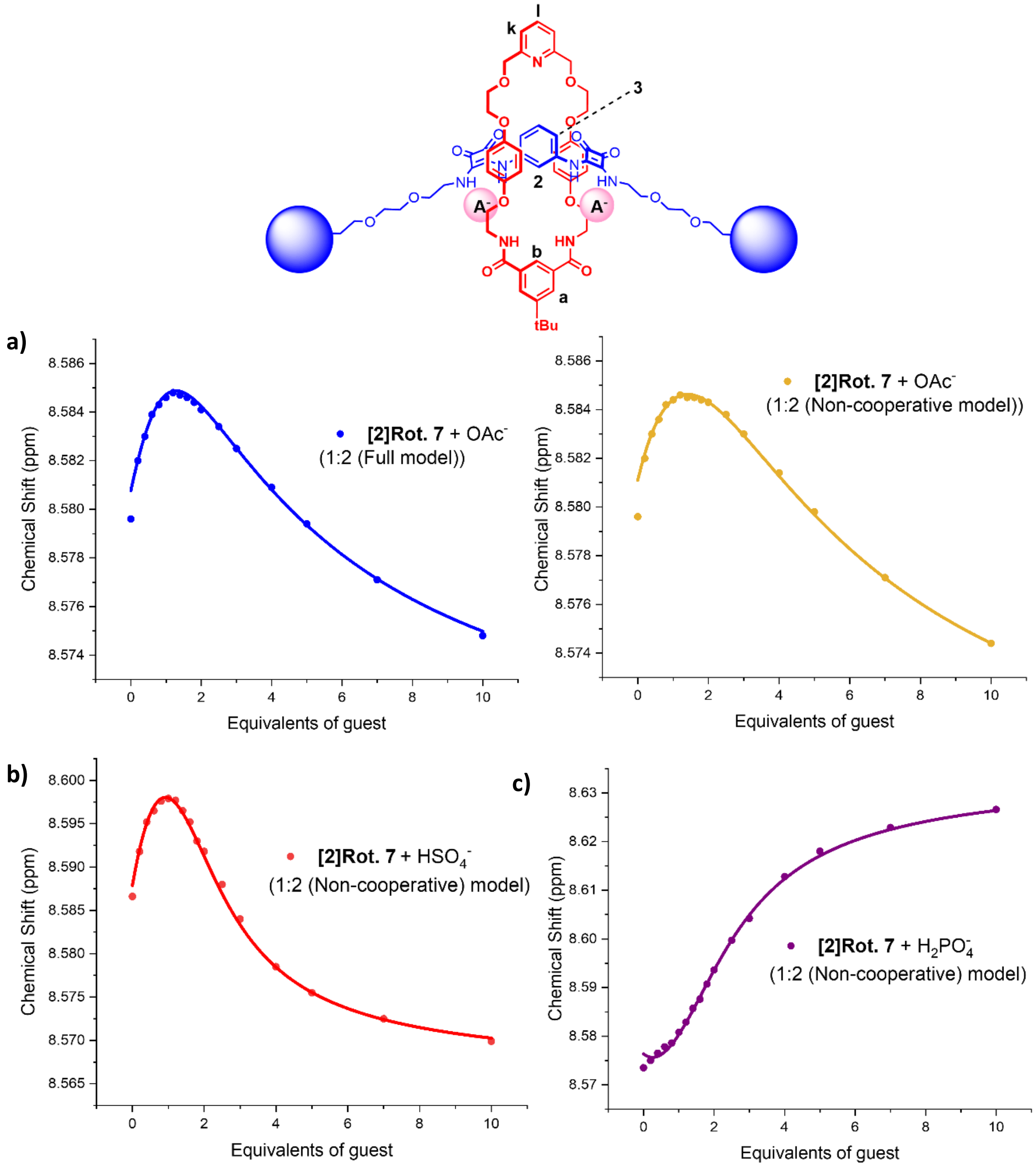

*Figure S52. Binding isotherms of rotaxane **7** fitted to most suitable 1:2 models, showing changes in chemical shift of internal benzene proton b upon increasing equivalents of a) acetate, b) hydrogen sulfate and c) dihydrogen phosphate anions.*

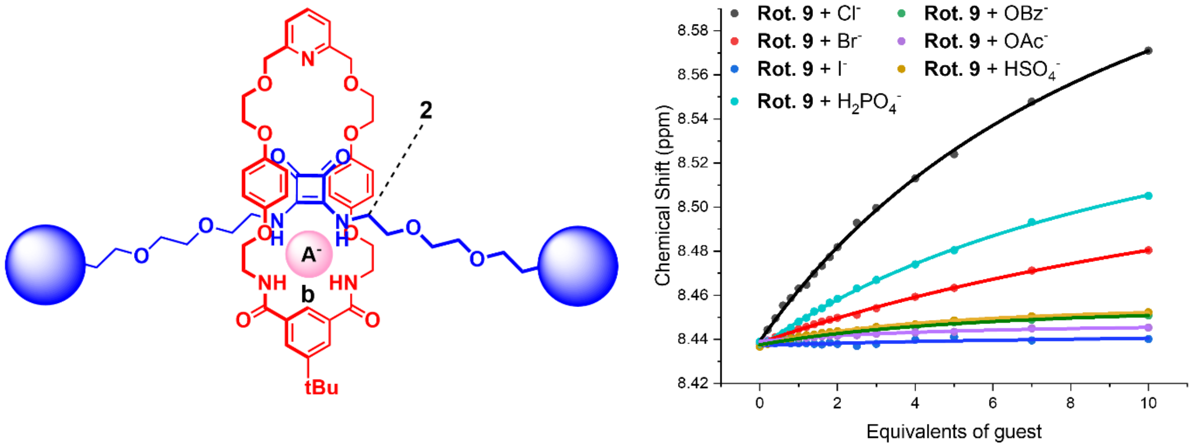

*Figure S53. Binding isotherms of [2]rotaxane **9** showing changes in chemical shift of internal benzene proton b upon increasing equivalents of various anions. (500 MHz, 298 K, 95:5-d6-acetone-D_2O).*

Determination of binding models in anion titrations

Sequential addition of most anions to the aryl-linked [2]rotaxane **7** resulted in downfield shifts in the proton signals corresponding to the chemical environments in the vicinity of the NH HB donors, namely macrocycle proton H_b and axle spacer proton H_2. However, upon addition of oxoanions such as OAc^-, HSO_4^- and $H_2PO_4^-$, the proton peaks proximal to the anion binding site initially moved downfield until 0.8-1 equivalents before shifting upfield upon subsequent anion addition up to 10 equivalents, alluding to the existence of complex equilibria in the system. This complex behaviour necessitated a more detailed study into the binding stoichiometries adopted by the interlocked host upon anion association.

To this end, five possible stoichiometric binding models including 1:1 and four variants of 1:2 host-guest binding models were considered for every anion titration, wherein the suitability of each binding model was determined through statistical analysis as detailed by Thordarson and co-workers.[42] Table **S1** outlines the four complex 1:2 binding models based on the correlations between the step-wise binding constants, K_1 and K_2 and the induced chemical shifts upon formation of 1:1 and 1:2 complexes, δ_1 and δ_2 respectively.

***Table S1:** Possible stoichiometric anion binding models for [2]rotaxanes **7** and **8** and the difference in their respective relationships between K_1, K_2, δ_1 and δ_2.[a]*

Binding models (Host: Guest)	Relationship between K_1 and K_2	Relationship between δ_1 and δ_2
1:1	Not applicable[b]	Not applicable[b]
1:2 (Full)	$K_1 \neq 4K_2$	$\delta_1 \neq 2\delta_2$
1:2 (Non-cooperative)	$K_1 = 4K_2$	$\delta_1 \neq 2\delta_2$
1:2 (Additive)	$K_1 \neq 4K_2$	$\delta_1 = 2\delta_2$
1:2 (Statistical)	$K_1 = 4K_2$	$\delta_1 = 2\delta_2$

[a] K_1 and K_2 represent the step-wise binding constants and δ_1 and δ_2 represent the chemical shifts induced by the formation of 1:1 and 1:2 stoichiometric host-guest complexes respectively. [b] Only K_1 and δ_1 are involved in 1:1 binding.

Accordingly, the covariance fit values of each model to the measured isotherms (Cov_{fit}) were obtained using Bindfit[41] and the corresponding covariance fit factors (Cov_{fit} factor) were determined. Wherein Cov_{fit} factor was defined as the ratio of the Cov_{fit} value acquired for a simple 1:1 binding model to the Cov_{fit} value generated for the various 1:2 binding models. As per Thordarson and co-workers, the lower the value of Cov_{fit}, the higher the corresponding Cov_{fit} factor value, and better the quality of the fit. This analysis however requires certain considerations to be taken in order to judge the suitability of each model including the number of fitted parameters employed, practical feasibility of the obtained binding constants and their associated errors etc. Therefore, for a complex binding model to be applicable, its Cov_{fit} value would have to be significantly lower than that of the simple 1:1 binding model (i.e., Cov_{fit} factor > 10). The binding models and corresponding K_1 and K_2 values obtained from this analysis for both bis-squaramide-based rotaxanes **7** and **8** are shown in Tables **S2** and **S3** respectively.

Consistent with the trends observed during spectral analysis, whilst the majority of the anions bound to [2]rotaxane **7** *via* a simple 1:1 stoichiometry, the binding isotherms of oxoanions- OAc^-, HSO_4^- and $H_2PO_4^-$ fit best to a 1:2 binding model. Interestingly, in the case of acetate anion the Cov_{fit} values of the 1:2 (Full) and 1:2 (Non-cooperative) binding models were 17- and 12-times lower than the Cov_{fit} value of the 1:1 model, indicating that both models could be considered appropriate descriptors of the binding data. It is important to note here however, that between the two likely models, although the 1:2 Full model with its higher Covfit factor value suggests that it fits the data better, the possibility that the elevated value might just be an artifact of the model incorporating a higher number of fitted parameters makes it difficult to conclusively ascertain the exact binding mode adopted by the receptor. Therefore, both 1:2 (Full) and 1:2 (Non-cooperative) models were considered while describing the binding of acetate. Alternatively, both oxoanions HSO_4^- and $H_2PO_4^-$, showed a preference for the 1:2 (Non-cooperative) binding model, rationalised by the unsuitable association constants attained with the other models.

Table S2: *Anion association constants (K_a/M^{-1}) for [2]rotaxane **7** [1.0 mM] in 95:5-d_6-acetone-D_2O at 298 K.*[a]

Anion	Binding Model (Host: Guest)	No. of fitted parameters	K_1 (M^{-1})	K_2 (M^{-1})	Cov_{fit} (x 10^{-3})	Cov_{fit} factor [b]
Cl^-	**1:1**	**3**	**556**	**-**	**0.204**	**1**
	1:2 (Full)	5	2653	345	0.141	1.4
	1:2 (Non-cooperative)	4	1078	270	0.161	1.3
	1:2 (Additive)	4	2278	333	0.150	1.3
	1:2 (Statistical)	3	2193	-	0.384	0.5
Br^-	**1:1**	**3**	**226**	**-**	**0.315**	**1**
	1:2 (Full)	5	4718(11)	190	0.184	1.7
	1:2 (Non-cooperative)	4	615	154	0.204	1.5
	1:2 (Additive)	4	621	147	0.288	1.0
	1:2 (Statistical)	3	629	-	0.291	1.0
I^-	**1:1**	**3**	**35**	**-**	**6.327**	**1**
	1:2 (Full)	5	7225(120)	71	5.226	1.2
	1:2 (Non-cooperative)	4	480	120	5.363	1.2

	1:2 (Additive)	4	170(14)	172(20)	5.5	1.1
	1:2 (Statistical)	3	76	-	6.309	1.0
**NO_3^-**	**1:1**	**3**	**42**	**-**	**4.929**	**1**
	1:2 (Full)	5	-	-	-	-
	1:2 (Non-cooperative)	4	186	47	4.901	1.0
	1:2 (Additive)	4	121	43(21)	4.909	1.0
	1:2 (Statistical)	3	92	-	4.926	1.0
**NO_2^-**	**1:1**	**3**	**124**	**-**	**0.957**	**1**
	1:2 (Full)	5	740	54	0.877	1.0
	1:2 (Non-cooperative)	4	213	53	0.992	0.9
	1:2 (Additive)	4	206	25	0.914	1.0
	1:2 (Statistical)	3	303		1.024	0.9
**OBz^-**	**1:1**	**3**	**701**	**-**	**0.886**	**1**
	1:2 (Full)	5	2202	210	0.111	7.9
	1:2 (Non-cooperative)	4	1051	263	0.376	2.3
	1:2 (Additive)	4	845	10(31)	0.835	1.0
	1:2 (Statistical)	3	2997	-	2.185	0.4
**OAc^-**	1:1	3	365	-	9.25	1
	1:2 (Full)	**5**	**3279(10)**	**232**	**0.557**	**17**
	1:2 (Non-cooperative)	**4**	**996**	**249**	**0.760**	**12**
	1:2 (Additive)	4	1094(24)	175(27)	9	0.9
	1:2 (Statistical)	3	1172	-	10	0.9
**HSO_4^-**	1:1	3	-	-	-	-
	1:2 (Full)	5	5(24)	$> 10^6$ (22)	29.017	-
	1:2 (Non-cooperative)	**4**	**5533(23)**	**1383**	**34.436**	**-**
	1:2 (Additive)	4	2912(49)	<0	164.5	-
	1:2 (Statistical)	3	2($>10^7$)	-	504.4	
**$H_2PO_4^-$**	1:1	3	183	-	14	1
	1:2 (Full)	5	-	54	0.877	15
	1:2 (Non-cooperative)	**4**	**3627**	**53**	**0.971**	**14**
	1:2 (Additive)	4	5($>10^3$)	$>10^5$	1.4	9.6
	1:2 (Statistical)	3	516	-	12	1.1

[a] Step-wise K_1 and K_2 values calculated using Bindfit[41] with different binding models by monitoring squaramide proton H_b. All anions added as their TBA salts; Error percentages less than 10% unless specified. [b] Cov_{fit} factor = Cov_{fit} of 1:1 binding model divided by the Cov_{fit} of the respective model. Binding models best fitted for the titration isotherms are highlighted in red.

Table S3: *Anion association constants (K_a/M^{-1}) for [2]rotaxane **8** [1.0 mM] in 95:5-d_6-acetone-D_2O at 298 K.*[a]

Anion	Binding Model (Host: Guest)	No. of fitted parameters	K_1 (M^{-1})	K_2 (M^{-1})	Cov_{fit} (x 10^{-3})	Cov_{fit} factor [b]
Cl^-	**1:1**	**3**	**621**	**-**	**0.65**	**1.0**
	1:2 (Full)	5	1581	71	0.291	2.2
	1:2 (Non-cooperative)	4	898	225	0.972	0.7
	1:2 (Additive)	4	1417	70	0.357	1.8
	1:2 (Statistical)	3	2355	589	2.26	0.3
Br^-	**1:1**	**3**	**226**	**-**	**0.217**	**1**
	1:2 (Full)	5	1505	145	0.191	1.1
	1:2 (Non-cooperative)	4	398	100	0.21	1.0
	1:2 (Additive)	4	558	99	0.216	1.0
	1:2 (Statistical)	3	627	157	0.27	0.8
I^-	**1:1**	**3**	**58**	**-**	**3.55**	1
	1:2 (Full)	5	211596	272	1.96	1.8
	1:2 (Non-cooperative)	4	163	41	3.04	1.2
	1:2 (Additive)	4	91	12	3.52	1.0
	1:2 (Statistical)	3	130	33	3.57	1.0
NO_3^-	**1:1**	**3**	**130**	**-**	**3.84**	1
	1:2 (Full)	5	375	11(13)	3.61	1.1
	1:2 (Non-cooperative)	4	224	56	3.89	1.0
	1:2 (Additive)	4	213	24(29)	3.75	1.0
	1:2 (Statistical)	3	320	80	3.94	1.0
NO_2^-	**1:1**	**3**	**160**	**-**	**1.35**	**1**
	1:2 (Full)	5	1078(11)	170	1.07	1.3
	1:2 (Non-cooperative)	4	681	-	1.07	1.3
	1:2 (Additive)	4	458	165	1.16	1.2
	1:2 (Statistical)	3	414	-	1.24	1.1
OBz^-	**1:1**	**3**	**916**	**-**	**0.785**	**1.0**
	1:2 (Full)	5	>10^4(13)	611	0.226	3.5
	1:2 (Non-cooperative)	4	2009	502	0.363	2.2
	1:2 (Additive)	4	786	<0	0.737	1.1
	1:2 (Statistical)	3	4856	1214	1.92	0.4
OAc^-	1:1	**3**	**668**	**--**	**1.36**	**1**
	1:2 (Full)	5	>10^4(15)	529	0.178	0.1
	1:2 (Non-cooperative)	4	1837	459	0.268	5.1
	1:2 (Additive)	4	524	<0	1.26	0.9
	1:2 (Statistical)	3	2913	728	1.47	1.1
HSO_4^-	1:1	**3**	**224**	**-**	**1.71**	**1.0**
	1:2 (Full)	5	158	<0	1.45	1.2
	1:2 (Non-cooperative)	4	364	-	1.88	0.9

	1:2 (Additive)	4	396	38(10)	1.54	1.1
	1:2 (Statistical)	3	617	-	2.15	0.8
$H_2PO_4^-$	1:1	**3**	**451**	**-**	**2.43**	**1.0**
	1:2 (Full)	5	>10^6 (706)	541	0.274	8.9
	1:2 (Non-cooperative)	4	2542	636	0.533	4.6
	1:2 (Additive)	4	1568(20)	590(18)	1.61	1.5
	1:2 (Statistical)	3	1668	417	1.71	1.4

[a] Step-wise K_1 and K_2 values calculated using Bindfit[41] with different binding models by monitoring squaramide proton H_b. All anions added as their TBA salts; Error percentages less than 10% unless specified. [b] Cov_{fit} factor = Cov_{fit} of 1:1 binding model divided by the Cov_{fit} of the respective model. Binding models best fitted for the titration isotherms are highlighted in red.

References

[34] A. Arun, A. Docker, H. Min Tay, P. D. Beer, *Chem. Eur. J.* **2023**, *29*, e202301446.

[40] M. R. Sambrook, P. D. Beer, J. A. Wisner, R. L. Paul, A. R. Cowley, F. Szemes, M. G. B. Drew, *J. Am. Chem. Soc.* **2005**, *127*, 2292-2302.

[41] V. Aucagne, K. D. Hänni, D. A. Leigh, P. J. Lusby, D. B. Walker, *J. Am. Chem. Soc.* **2006**, *128*, 2186-2187.

[42] P. Thordarson, *Chem. Soc. Rev.* **2011**, *40*, 1305-1323.

[43] E. N. W. Howe, M. Bhadbhade, P. Thordarson, *J. Am. Chem. Soc.* **2014**, *136*, 7505-7516.
